# The *tin1* gene retains the function of promoting tillering in maize

**DOI:** 10.1038/s41467-019-13425-6

**Published:** 2019-12-06

**Authors:** Xuan Zhang, Zhelong Lin, Jian Wang, Hangqin Liu, Leina Zhou, Shuyang Zhong, Yan Li, Can Zhu, Jiacheng Liu, Zhongwei Lin

**Affiliations:** National Maize Improvement Center; Center for Crop Functional Genomics and Molecular Breeding; Joint Laboratory for International Cooperation in Crop Molecular Breeding, Ministry of Education; Beijing Key Laboratory of Crop Genetic Improvement, Laboratory of Crop Heterosis and Utilization, China Agricultural University, Beijing, 100193 China

**Keywords:** Agricultural genetics, Agricultural genetics, Natural variation in plants, Plant domestication

## Abstract

Sweet maize and popcorn retain tillering growth habit during maize diversification. However, the underlying molecular genetic mechanism remains unknown. Here, we show that the retention of maize tillering is controlled by a major quantitative trait locus (QTL), *tin1*, which encodes a C2H2-zinc-finger transcription factor that acts independently of *tb1*. In sweet maize, a splice-site variant from G/GT to C/GT leads to intron retention, which enhances *tin1* transcript levels and consequently increases tiller number. Comparative genomics analysis and DNA diversity analysis reveal that *tin1* is under parallel selection across different cereal species. *tin1* is involved in multiple pathways, directly represses two tiller-related genes, *gt1* and *Laba1/An-2*, and interacts with three TOPLESS proteins to regulate the outgrowth of tiller buds. Our results support that maize *tin1*, derived from a standing variation in wild progenitor teosinte population, determines tillering retention during maize diversification.

## Abstract (web summary)

Unlike the other domesticated maize, sweet maize and popcorn retain tillering growth habit, but the underlying mechanism is unknown. Here, the authors identify a transcription factor *tin1* that maintains outgrowth of tiller independent of *tb1* and show its conservation in foxtail millet and rice.

## Introduction

**Fig. 1:**
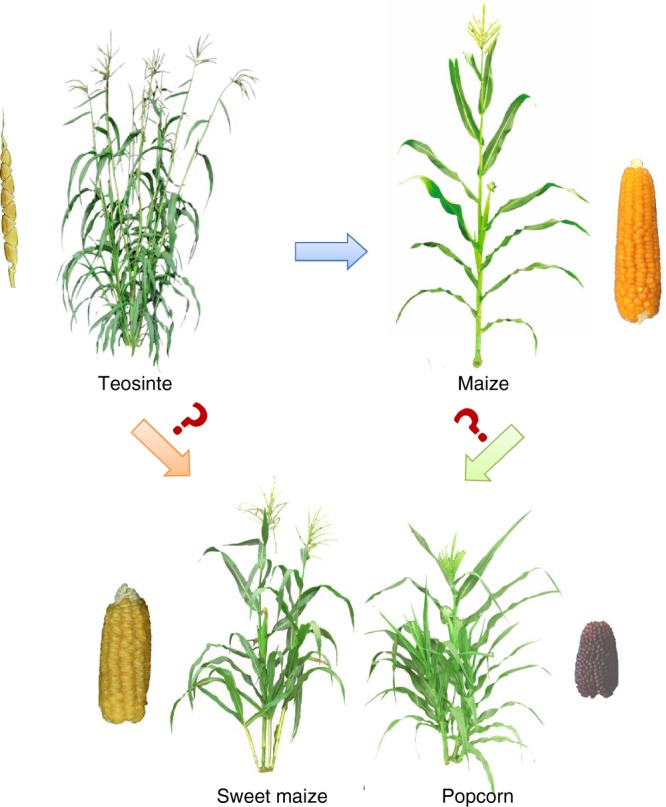
Sweet maize and popcorn retained a tillering habit. Maize wild progenitor, teosinte (left), has multiple tillers and two-rowed ears, while domesticated maize (right) generally has a single strong stalk bearing a large ear. However, some maize types, including sweet corn (left) and popcorn (right), retain a tillering habit like that of teosinte. How and why sweet corn and popcorn retained this tillering habit remain unknown.

Maize was domesticated from its wild progenitor teosinte approximately 10,000 years ago^1^. Human selection has greatly reshaped maize during domestication. A revolutionary transition was from multiple tillers in teosinte to a single stalk in maize^2^. Teosinte generally has multiple tillers and bears small ears with a few kernels on the nodes of tillers, which occupy more space and repress the growth of other grasses and distribute seeds on the whole plant, making this a powerful survival strategy in the wild. By contrast, modern maize generally has a single thick, fibrous stalk that allows it to be cultivated at high density and then bear heavy ears with good yield potential^3^. This common domestication path, from multiple tillers to a single stalk, is generally present in maize^3^ (Fig. 1). However, the diversification of two types of maize, including sweet maize and popcorn, deviated from this common domestication path with sweet maize and popcorn partially retained the tillering habit, like teosinte^4–6^. In most cases, sweet maize and popcorn generally bear two to three tillers with ears (Fig. 1). The molecular genetic basis underlying how these types of maize retained the tillering habit remains unknown.

The development of maize tiller is controlled by a complex gene regulatory network. A critical gene, *teosinte branched1* (*tb1*), encoding a TCP protein, was responsible for the key change from the teosinte plant with multiple branches to maize with a single stalk^3^. The gene *grassy tillers1* (*gt1*) encoding a homeodomain protein can response to shade, which depends on *tb1*. The *gt1* gene functions as a tiller repressor, and high *gt1* expression suppresses the outgrowth of tiller buds^7^. *tassels replace upper ears1* (*tru1*), encoding a protein with an ankyrin-repeat domain and directly targeted by *tb1*, also plays as a tiller repressor and high *tru1* transcription suppresses the outgrowth of maize axillary bud^8^. In sweet maize, *sugary 1* changes the carbohydrate metabolism balance and may promote tiller bud outgrowth^9^. However, whether other genes contribute to maize tiller development remains little understood.

Cereal crops such as maize, wheat, rice, barley, and sorghum were domesticated thousands of years ago. Although these crops were domesticated from different wild progenitors by different ancient human groups in different geographical regions, they all underwent systemic and parallel changes during the domestication and improvement process^10,11^. Most of the genome sequences from these cereals are available^12–15^. However, whether these parallel changes, such as the transition from bush to compact plant architecture, share the same genetic basis remains largely uncharacterized.

In this study, we combine fine mapping and association mapping to narrow down a major QTL of tiller number, *tin1*, within a 3.9-kb fragment on maize chromosome 7. We show that *tin1* encodes a C2H2-zinc-finger transcription factor. A splice-site variant from G/GT to C/GT in sweet/popcorn leads to intron retention, then may enhance mRNA stability and transcription of *tin1*, and finally increases tiller number. Comparative genomics analysis reveals that large deletions occurred in foxtail millet and rice *tin1* syntenic blocks during domestication. DNA diversity analysis further reveals that *tin1* is under parallel selection in cereals. During the outgrowth of tiller buds, *tin1* is involved in multiple pathways including cellulose synthesis, photosynthesis, and hormonal responses; TIN1 directly represses two tiller-related genes, *gt1* and *Laba1/An-2*, and interacts with three TOPLESS proteins. Our results suggest that maize *tin1*, derived from a standing variation in wild progenitor teosinte population, retains tillering after the fixation of the key gene *tb1* during maize diversification.

## Results

### *tin1* is a major QTL for tiller number in maize

To study why sweet maize and popcorn can produce tillers, a typical sweet maize, P51, was crossed with a maize elite inbred line, B37, to construct a recombinant inbred line (RIL) population (Supplementary Fig. 1a). P51 generally produced two to three tillers and at least three effective ears per plant. By contrast, B37 produced a single stalk with a maximum two effective ears per plant. QTL analysis identified three QTLs for tiller number in the RIL population with 232 individuals (Supplementary Fig. 1b, c). Among which, a major QTL, accounted for 9.5% of the total phenotypic variation, was localized on the short arm of chromosome 7 (Supplementary Fig. 1c). This major QTL was then designated as *tiller number 1* (*tin1*).

**Fig. 2:**
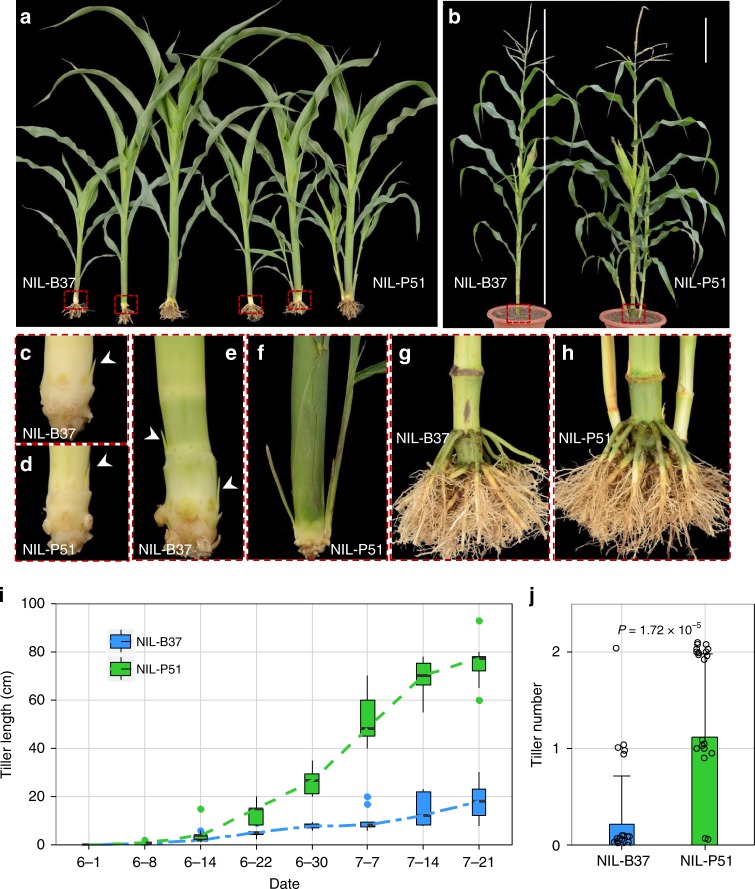
Phenotypes of maize *tin1*. **a**, **b** Phenotype comparisons between two near-isogenic lines (NILs) before the jointing stage (**a**) and at the flowering stage (**b**). **c**–**f** A close-up view of the tiller base at 25 (**c, d**) and 35 (**e**, **f**) days after planting (DAP) in NIL-B37 (**c**, **e**) and NIL-P51 (**d**, **f**). The tiller buds from NIL-B37 remained dormant all the time (**e**), while the tiller bud from NIL-P51 grew out at 35 DAP (**f**). **g**, **h** A close-up view of the tiller base at flowering stage from NIL-B37 (**g**) and NIL-P51 (**h**). **i** Growth curves of tiller bud development from NIL-B37 (blue) and NIL-P51 (green). Box edges represent quartiles, and the medians were shown with the central line in the boxes. **j** Final tiller number in NIL-B37 (blue) and NIL-P51 (green). Error bar, SD (*n* = 28, 26). *P*-values were determined by two-tailed Student’s *t*-test. Scale bar, 25 cm. The source data underlying **i**, **j** are provided as a Source Data file.

To precisely estimate the genetic effect of *tin1*, a pair of near-isogenic lines (NILs) was then generated, which were derived from a residual heterozygous line (RHL, F_6_), containing a heterozygous genomic fragment at *tin1* and homozygous genotypes at most other loci (Supplementary Fig. 1d). Both NIL-B37 and NIL-P51 plants bore small visible tiller buds in the early seedling stage (Fig. 2a–f). However, the tiller buds from NIL-B37 remained dormant in all stages (Fig. 2i). The tiller buds of NIL-P51 started to grow out at the jointing stage (6–14, Fig. 2i) and tiller length reached a maximum at the end of the grain filling stage (7–21, Fig. 2i). Finally, tiller number was significantly greater in NIL-P51 compared with NIL-B37 (Fig. 2b, g, h, j).

### High-resolution mapping of maize *tin1* gene

**Fig. 3:**
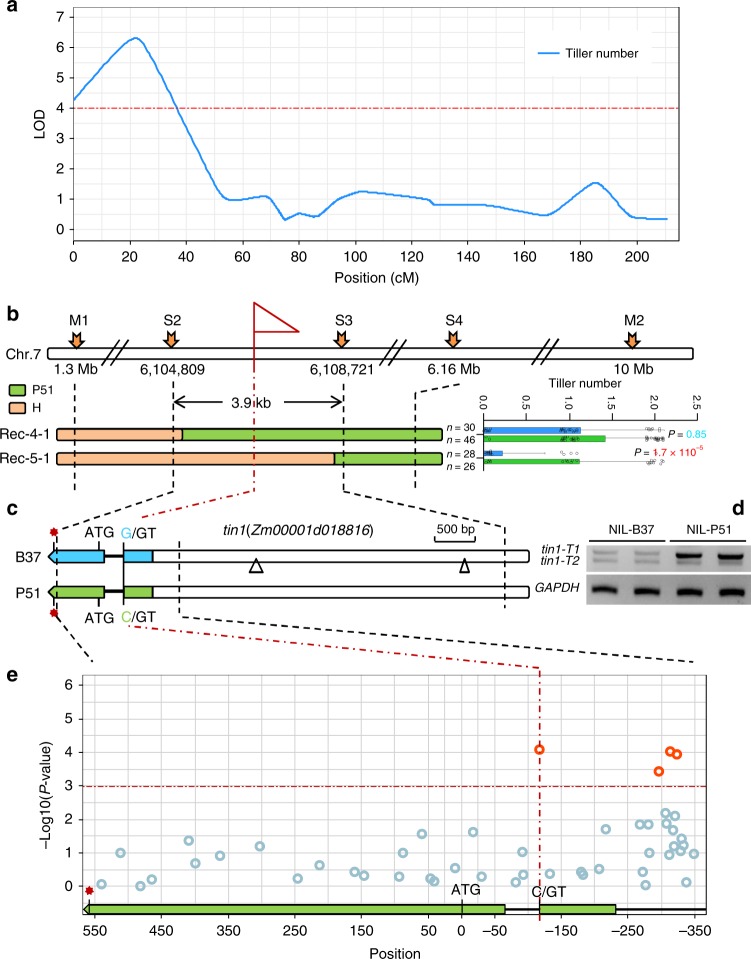
High-resolution mapping of *tin1*. **a** QTL mapping identified a major QTL, *tin1*, on chromosome 7 for tiller number in a RIL population derived from crosses between an elite inbred line B37 and a sweet maize P51. The red dashed line represents the significant logarithm of the odds (LOD) threshold (3.99) at the 0.05 level. **b** Fine mapping narrowed down *tin1* within a genomic fragment of 3.9 kb through a progeny test. Green and orange bars represent the chromosomal fragments from P51 and heterozygous plants, respectively. The *P*-values from the progeny tests were highlighted in red (significant) and blue (non-significant) using Student’s *t*-test. Error bar, SD (*n* = 30, 46; 28, 26). Green and orange bars signified the chromosomal fragments from the P51 and heterozygous plants. Scale bar, 500 bp. **c** Only one gene (*Zm00001d018816*) was placed in this fine-mapping region. Blue and green bars and black lines represented exons and introns, and blank box signified promoter region. Scale bar represents 500 bp. The splice-site variant from G/GT to C/GT was present in P51. Only two insertions over 100 bp were shown in the promoter region. Red star, stop codon. Triangle, insertion. **d** RT-PCRs for two transcripts, *tin1-T1* and *tin1-T2*. The transcript of a housekeeping gene *GAPDH* was used as the internal control. **e** Association mapping. Significant signals were highlighted in red and non-significant signals were marked in light blue. The gene structure of *tin1* was shown and the start codon was regarded as position 0. The horizontal red dashed line represents signified the 5% significance threshold with Bonferroni correction for 48 tests. The source data underlying **d** are provided as a Source Data file.

Genetic linkage analysis in the RIL population showed that *tin1* was placed between two markers M1 and M2 on short arm of chromosome 7 (Fig. 3a, b). To fine-map *tin1*, we then selected a RHL and generated a large population with 10,704 individuals (see Methods). Following a modified progeny testing strategy, one single nucleotide polymorphism (SNP) and eight newly developed simple sequence repeat (SSR) markers finally narrowed down the *tin1* within an 3.9-kb region, flanked by the markers S2 and S3 (Fig. 3b, c, Supplementary Fig. 2, and see Methods). Only one gene (*Zm00001d018816*) was annotated in this 3.9-kb fine-mapping region (Fig. 3c), according to the B73 reference genome sequence (https://maizegdb.org/, V4), and thus this gene became the candidate for *tin1*.

The gene *Zm00001d018816* contains two exons and one intron (Fig. 3c). This intron is in its 5′ untranslated region (5′ UTR) (Fig. 3c). Sequence analysis of the 3.9-kb fine-mapping region revealed eight SNPs and three insertion/deletions (Indels) in the coding sequence (CDS) and 5′ UTR, and 20 Indels and 79 SNPs in the promoter region between these two parental lines (Fig. 3c and Supplementary Data 1). A luciferase transient expression assay of the 3.1-kb promoter region revealed no significant difference in the luciferase activity present between the two parental lines B37 and P51 (Supplementary Fig. 3). A conspicuous SNP from G/GT in B37 to C/GT in P51 was found in the 5′ splice site of the intron of the gene.

We next conducted sequencing analysis of a 1-kb fragment containing the CDS, the 5′ UTR and a 197-bp promoter region in diverse maize inbred lines from around the world (see Methods). This large sequencing analysis revealed 48 SNPs in this 1-kb fragment. Association mapping with these 48 SNPs then detected strong signals associated with tiller number, present at positions −117, −297, −313, and −324 (*P* < 1.04 × 10^−3^, *F-*test) (Fig. 3e). The most significant association signal was present at the 5′ splice site of the intron (Fig. 3e) in the 5′ UTR of *tin1*. The other three variants with strong signals at positions −297, −313, and −324 were in high linkage disequilibrium (*r*^2^ > 0.95) with the 5′ splice-site variant (Supplementary Fig. 4), thus these variants were highly correlated with tiller number. This result indicated that the gene *Zm00001d018816* corresponding to *tin1* controlled tiller number in maize. The association mapping supported that the 5′ splice-site variant from G/GT to C/GT in the intron of *tin1* in P51 with tillers was the causal functional variant of *tin1*.

Although the luciferase transient expression of promoter did not detect the changed signals in leaf between the two parental lines B37 and P51 (Supplementary Fig. 3), the variants in the promoter with high LD with the splice-site variant might also contribute to the expression levels of *tin1* in tiller bud (Fig. 3e), because the effect of the promoter of *tin1* might be tissue dependent.

### Maize *tin1* splice-site variant from G/GT to C/GT

**Fig. 4:**
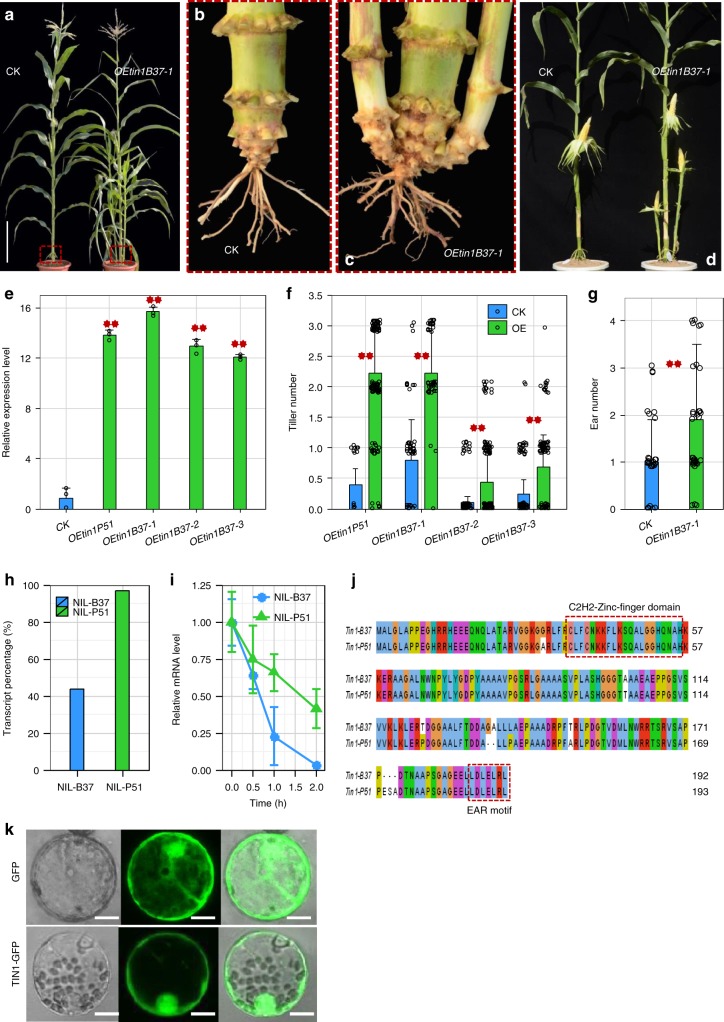
Gene function analysis of *tin1*. **a**–**d** Transformation with an overexpression construct carrying *tin1* coding sequence from B37 (*OEtin1B37*). The transgenic plants (*OEtin1B37-1*) exhibited tillering (**a**) and bore more ears than the control (CK) nontransgenic plant. Scale bar, 30 cm. **d** A close-up view of tiller bases (**b**, **c**). **e** The transgenic plants had greatly enhanced transcript levels of *tin1* compared to those of the control non-transgenic plants (CK). Error bar, SD (*n* = 3). **f**, **g** The transgenic plants bored significantly more tillers (**f**) and ears (**g**) than those of the control non-transgenic plants. Blue and green bars in (**f**) represented the control (CK) and overexpression transgenic plants. *P*-values were determined by two-tailed Student’s *t*-test. Two red asterisks represented strong significance (*P* < 0.005). Error bar, SD (*n* = 20, 128; 63, 50; 110, 137; 97, 92; 33, 44). **h** The percentages of *tin1-T1* transcript with an intron relative to the total number of transcripts from NIL-B37 and NIL-P51. **i** *tin1* mRNA stability analysis. Error bar, SD (*n* = 3). Blue and green lines, NIL-B37 and NIL-P51. **j** Protein sequence alignment of *tin1* gene from B37 and P51. The two red dashed boxes signified a C2H2-zinc-finger domain and an EAR motif. **k** The subcellular location of TIN1-GFP fusion protein in maize leaf protoplasts. Scale bar, 5 μm. The source data underlying **e**–**g**, **i** are provided as a Source Data file.

The 5′ rapid amplification of cDNA end (5′ RACE) revealed two alternatively spliced transcripts (*tin1-T1 *and *tin1-T2*) present in both B37 and P51 (Fig. 3d). The *tin1-T1* transcript contained an unspliced intron (Supplementary Fig. 5). By contrast, the *tin1-T2* transcript did not have this intron (Supplementary Fig. 5). The percentage of unspliced *tin1-T1* transcript was 96.7% and 46.8% of the total amount of transcripts in P51 and B37, based on 5′ RACE (Fig. 4h), indicating that intron retention was greatly increased in P51 *tin1* transcripts. The amount of *tin1-T1* transcript was greatly enhanced in P51 due to intron retention based on RT-PCRs (Fig. 3d).

To identify how intron retention elevates the transcript of *tin1*, we analyzed mRNA stability of *tin1* from NIL-B37 and NIL-P51. The *tin1* mRNA decayed more rapidly in NIL-B37 compared with NIL-P51 (Fig. 4i and see Methods) after incubation with a transcription inhibitor, actinomycin D. All the above results suggested that the intron retention originated from the splice-site variant of C/GT greatly enhanced the mRNA stability of *tin1*, then might elevate the transcript levels of *tin1*, to finally increase tiller number in maize.

### Overexpression of *tin1* increased tiller number in maize

To identify whether the gene *Zm00001d018816* corresponding to *tin1* is responsible for tiller number in maize, we conducted transformations using two overexpression constructs (*OEtin1P51* and *OEtin1B37*), containing the CDS of *Zm00001d018816* from both the no-tillering parent B37 and the tillering parent P51, driven by the maize *Ubiquitin* promoter (Fig. 4a–g and Supplementary Fig. 6). These two constructs were introduced into maize inbred line B73, and one and three transformation events were obtained for *OEtin1P51* and *OEtin1B37*, respectively. The plants from these four events were then backcrossed with the parental line B37 without tillers and self-pollinated for two generations. Tillers were strongly and significantly increased in the transgenic plants with overexpressed *tin1* gene compared with the respective controlled non-transgenic plants (Fig. 4a–f, *P* < 1.0 × 10^−5^, two-tailed Student’s *t*-test). This result confirmed that the gene *Zm00001d018816* corresponding to *tin1* controlled tiller number in maize.

### Maize *tin1* controls the outgrowth of the tiller bud

The *tin1* encodes a protein with 192 amino acid (aa) residues with a C2H2-type zinc-finger domain (aa 47–56) and an EAR-like active repression motif (aa 186–192) (Fig. 4j). TIN1-GFP fusion protein was localized in the nucleus and cytoplasm in both onion epidermal cells and maize protoplasts (Fig. 4k and Supplementary Fig. 7). These facts signified that *tin1* harboring a C2H2-type zinc-finger domain functions as a transcription factor.

Maize tillers originate from axillary meristems in leaf axils, which form tiller buds that elongate and develop into tillers. Phenotypic comparisons between NIL-B37 and NIL-P51 indicated that *tin1* controls tiller bud outgrowth but does not influence tiller bud formation (Fig. 2c–f, i). RT-qPCR showed that *tin1* was mainly expressed in the tiller bud, leaf, shoot apical meristem (SAM), and root, and weakly expressed in the inflorescence meristem (IM), ear, tassel, and leaf auricle (Supplementary Fig. 8). The expression of *tin1* was substantially higher in NIL-P51 than in NIL-B37 in both the tiller bud and SAM. However, the expression of *tin1* was equal between NIL-P51 and NIL-B37 in leaf and root. RT-PCRs then revealed that only the *tin1-T1* transcript with the intron was present and the *tin1-T2* transcript without the intron was absent in leaf and root (Supplementary Fig. 9). This result suggested that alternative splicing is tissue dependent. Therefore the splice-site variant from G/GT to C/GT did not change the expression levels of *tin1* between the two NILs in leaf and root.

**Fig. 5:**
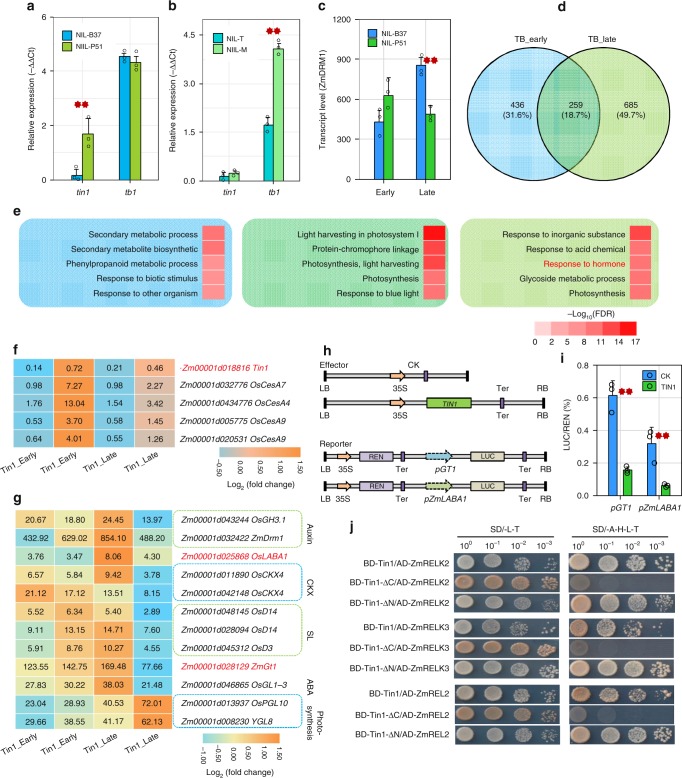
*tin1* regulates multiple pathways. **a** Significant expression change was present from *tin1* but not from *tb1* between NIL-B37 (blue bar) and NIL-P51 (green bar). **b** Significant expression difference occurred from *tb1* but not from *tin1* in NIL-T (blue bar) with a teosinte *Tb1* allele and NIL-M (green bar) with a maize *tb1* allele. **c** The marker gene *ZmDRM1* for tiller dormancy was repressed by *tin1* in the late stage of the tiller bud development based on *q*-values. Blue and green bars, NIL-B37 and NIL-P51. **d** The number of overlapping differentially expressed genes (DEGs) shared between the early (TB_early) and late (TB_late) stages of tiller bud development. **e** The top five enriched go ontology (GO) terms in the specific DEG sets from early stage plants (blue), the overlapping DEG set between the two stages (dark green) and the specific DEG set from the late stage plants (light green). These GO terms were shown in three different colors that correspond to the colors in panel D. Heat color bar, −Log_10_ (FDR). **f** *tin1* and four tiller-related cellular synthase genes were differentially expressed between the two stages. **g** 12 tiller-related DEGs were present in the late stage, which were related to auxin, cytokinin oxidase (CKX), strigolactone (SL), abscisic acid (ABA) and photosynthesis. Heat color bar, Log_2_ (fold change). **h**, **i** Dual-luciferase transient expression assays for *GT1* and *Laba1*, the transcriptions of the reporters under the controls of *GT1* and *Laba1* promoters were significantly repressed by the effector over-expressing *tin1*. **j** A Yeast two-hybrid assay showed that three TPL-related proteins directly interact with the *tin1* EAR motif in the C-terminus. *P*-values were determined by two-tailed Student’s *t*-test. Two red stars, *P* < 0.01. Error bar, SD (*n* = 3). The source data underlying  **a**, **b**, **i** are provided as a Source Data file.

Another key gene *tb1* also controls the elongation of tiller buds in maize^3^. The *tb1* gene showed similar expression levels in the NIL-B37 and NIL-P51 plants (Fig. 5a). In contrast to this, the expression of *tin1* did not appear significantly different in the NILs with teosinte *Tb1* and maize *tb1* alleles, respectively (Fig. 5b). This result suggested that maize *tin1* might work independently of *tb1* to control the outgrowth of tiller buds.

### Maize *tin1* regulates multiple tiller-related genes

To identify how *tin1* control tiller outgrowth, we conducted RNA-seq analysis using tiller buds from the first leaf axil of NIL-B37 and NIL-P51 at 25 days after planting (DAP) and 30 DAP (denoted as early and late stages, respectively). Tiller buds form and remain dormant in both NIL-B37 and NIL-P51 before 25 DAP (Fig. 2i). Tiller buds remain dormant after 25 DAP in NIL-B37, but they start to grow out steadily in NIL-P51 at this time. The expression of *ZmDRM1*^16,17^, a marker gene of bud dormancy, was consistent with this change in tiller development (Fig. 5c). Low expression levels of *ZmDRM1* were present in both NIL-B37 and NIL-P51 at 25 DAP, then approximately two-fold higher expression levels were observed in the NIL-B37 plants relative to the NIL-P51 plants at 30 DAP (Fig. 5c).

RNA-seq detected 695 and 944 differentially expressed genes (DEGs) between the two NILs at early and late stages, respectively (Fig. 5d, Supplementary Fig. 10 and Supplementary Data 2). Only 259 DEGs were shared between the two stages (Fig. 5d). A gene ontology (GO) analysis next revealed that early stage specific DEGs were most enriched in secondary metabolic process and late stage specific DEGs were significantly enriched in hormonal response (Fig. 5e and Supplementary Data 3–5). Interestingly, the top four significantly enriched GO terms, shared between the early and late stages, relating to biological process, molecular function and cellular component, were generally connected to photosynthesis, including light harvesting in photosystem I, pigment binding and photosystem I (Fig. 5e and Supplementary Data 5). The strongly enriched GO terms related to photosynthesis suggested that the DEGs influencing photosynthesis play an important role in both tiller bud formation and outgrowth in maize.

To identify possible direct transcriptional targets of *tin1*, we then collected 75 reported rice and three reported maize genes (*tb1*^3^, *gt1*^7^ and *tru1*^8^) related to tiller development (Supplementary Data 6). We found that maize homologs of 15 of the 75 tiller-related genes in rice showed differential expression between the pair of NILs in at least one stage, including four cellulose synthase genes, *pgl10*^18^ and *ygl8*^19^ for leaf chlorophyll content, and nine genes for hormone responses (Fig. 5f, g and Supplementary Data 2). Among the three tiller-related genes from maize, only *gt1* showed significant downregulation in the tiller buds of NIL-P51 at the late stage (Fig. 5g). The transcription patterns of these 16 genes observed by RNA-seq were further confirmed by real-time RT-qPCR (Supplementary Fig. 11). We next selected eight of these genes, and performed dual-luciferase transient expression assays in maize protoplasts to test the effect of *tin1* on their expression (Fig. 5h and Supplementary Fig. 12). In rice, *Laba1*^20^ (also named as *An-2*^21^) controls awn, tiller number and yield. Overexpression of *tin1* (effector) significantly suppressed the activity of the luciferase (reporter) activity driven by the *gt1* and *Laba1/An-2* promoters (Fig. 5i), indicating that the TIN1 protein directly targeted the promoters of *gt1* and *Laba1/An-2* and then suppressed transcriptions during maize tiller bud outgrowth.

TOPLESS^22^ (TPL) and TPL-related proteins (TPRs)^23^ have been reported to function as co-repressors in various plant hormone signaling and tiller developmental pathway. The EAR motif is a hallmark of the TPL and TPR interacting proteins^22,23^. There are four TPL/TPR/REL genes expressed in maize tiller bud, namely *RELK1* (Zm00001d040279), *RELK2* (Zm00001d028481), *RELK3* (Zm00001d047897) and *REL2* (*RAMOSA1 ENHANCER LOCUS2*^24^, Zm00001d024523)^24^ (Supplementary Fig. 13). Only *RELK1* showed low expression level in maize tiller bud (Supplementary Fig. 13). In rice, the homolog of *REL2* (also known as *OsASP1*, *OsREL2* or *OsLIS-L1*) functions in tiller and spikelet development^25^. We performed a yeast two-hybrid assay to detect the interactions between TIN1 and the three highly expressed maize TPL/TPR/REL proteins (RELK2, RELK3 and REL2). All the truncated N-terminal TIN1 protein interacted with the TPL/TPR/REL proteins, while all the truncated C-terminal TIN1 protein without the EAR motif did not interact with the TPL/TPR/REL proteins (Fig. 5j, see Methods). This result suggested that TIN1 directly interacts with these three TPL/TPR/REL proteins through the C-terminal EAR motif during maize tiller development.

### Parallel selection of *tin1* in cereals

**Fig. 6:**
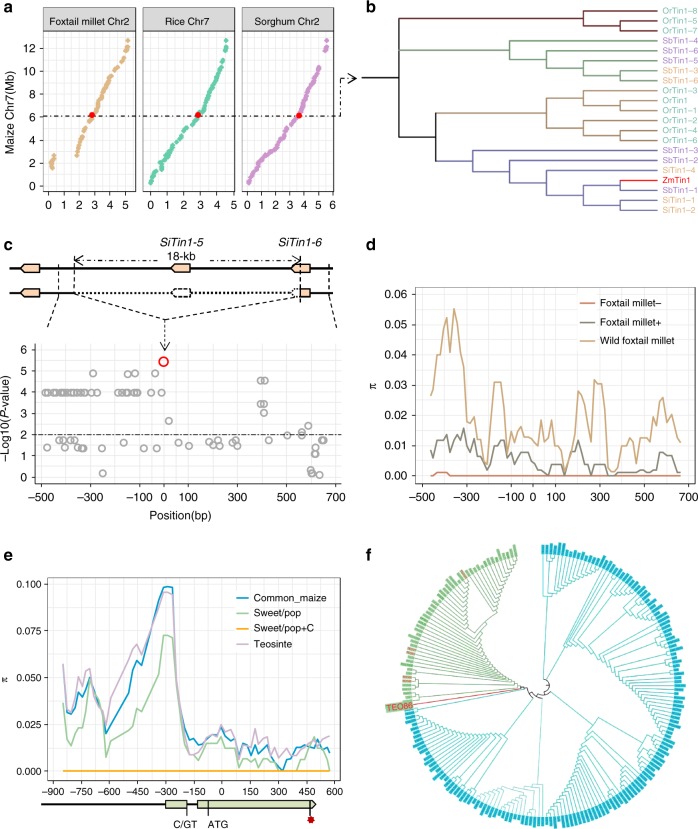
Parallel selection of *tin 1* among different species. **a**, Comparative genomic analysis of the *tin1* locus among maize, rice, foxtail millet, and sorghum. Red dots represented the *tin1* locus. **b** Phylogenetic tree for *tin1* orthologs among different cereal species. Maize *tin1* was marked in red. **c** Association mapping test between foxtail millet *tin1* and tiller number. An 18-kb deletion containing one and a half *tin1* copies was present in domesticated foxtail millet. The red dot signified the causal variant for tiller number. The horizontal red dashed line represents signified the 5% significance threshold with Bonferroni correction for 76 tests. **d** DNA diversity analysis for foxtail millet *tin1*. The deletion break point was regarded as position 0. +  and −, with and without the 18-kb sequence, respectively. **e** DNA diversity analysis for maize *tin1*. The gene structure of *tin1* was shown on the horizontal axis, the start codon was set as position 0. +C, with the splice-site variant of C/GT. Red star, stop codon. **f** Phylogenetic tree analysis for maize *tin1* in a global natural maize population. The teosinte lines with the splice-site variant of *tin1* were highlighted in red.

To ask whether *tin1* has an evolutionarily conserved function in controlling the outgrowth of tiller buds among different cereals, we conducted comparative mapping across maize, rice, foxtail millet, and sorghum, for which the entire genomes are well sequenced. Comparative mapping identified a syntenic block corresponding to the *tin1*, containing four highly conserved chromosomal segments on maize chromosome 7, foxtail millet chromosome 2, rice chromosome 7, and sorghum chromosome 2, respectively (Fig. 6a). Interestingly, rice orthologs of *tin1* turned out to be a reported gene cluster, which contained eight copies of *tin1* (*prog1*), responsible for tiller number and the key transition of plant architecture from the prostrate tiller of typical wild rice to the erect tiller of cultivated rice during domestication^26,27^. Similarly, there are five copies of the *tin1* ortholog in cultivated foxtail millet and six copies in cultivated sorghum, respectively. Phylogenetic analysis revealed a high level of similarity among maize, rice, foxtail millet, and sorghum TIN1 proteins. Most of these proteins contained two highly conserved zinc-finger and EAR-like domains (Fig. 6b and Supplementary Fig. 14), signifying that the *tin1* genes might have a conserved function in the regulation of tiller development.

Sequence comparison of *tin1* between domesticated and wild foxtail millet revealed that an 18-kb fragment was absent in some domesticated foxtail millet lines (Fig. 6c, Supplementary Data 7 and Supplementary Fig. 15). The 18-kb fragment contained an entire copy of *tin1* (*SiTin1-5*) and a partial 81-bp CDS of *tin1* (*SiTin1-6*). We next performed a large DNA sequence analysis in the breakpoint of the large deletion of 18 kb including 695-bp upstream and 489-bp downstream sequences, in 24 wild and 65 domesticated diverse accessions from different geographical locations across the world (see Methods). Large sequence analysis identified 76 variants in the 1.2-kb region flanking the large deletion (Fig. 6c). Association testing with these variants detected the strongest signal (*P* = 3.81 × 10^−6^, *F*-test) present at the site of the large 18-kb deletion, associated with tiller number in foxtail millet (Fig. 6c). The deletion of one and a half copies of *tin1* resulted in the reduction of an average 4.75 tillers per plant in domesticated foxtail millet compared to wild foxtail millet. In sorghum, QTL mapping identified a major QTL for tiller number in this *tin1* syntenic block between wild and domesticated sorghums, which accounted for 6.5% of the total phenotypic variation (Supplementary Fig. 16). These results suggested that the function of *tin1* remains conserved among these cereals.

To investigate whether *tin1* was under selection in different species, we performed DNA diversity analysis. In foxtail millet, wild foxtail millet showed apparent higher DNA diversity (*π* = 0.01887) than domesticated foxtail millet with or without the large deletion (Fig. 6d). The diversity of domesticated foxtail millet with the large deletion was extremely low (*π* = 0.00005). Tajima’s *D* test^28^ significantly (*P* < 0.01, *D* test, *F*st = 0.34 between wild and domesticated foxtail millet) rejected the neutral null model for the 1.2-kb region flanking the large deletion of 18 kb in foxtail millet (Fig. 6d). The sequences of sweet maize and popcorn with the C/GT allele were completely the same in the promoter, 5′ UTR and CDS of the *tin1* gene (Fig. 6e). However, DNA diversity of this fragment in all these sweet/popcorn with G/GT and C/GT was still high, in comparison to those in teosinte and common maize excluding sweet/popcorn (Fig. 6e). Genome-wide composite likelihood ratio (XP-CLR) analysis^29^ did not detect any selection signals in maize *tin1* (see Methods and Supplementary Fig. 17) between sweet/popcorn and teosinte or common maize. In rice, *tin1* locus has been reported to have undergone strong selection during domestication^26^. In addition, we identified a major QTL for tiller number linked with sorghum *tin1* in a RIL population between a wild and a domesticated sorghum lines (Supplementary Fig. 16). The selection of *tin1* resulted in the decreased tiller number during rice, foxtail millet, and sorghum domestications. These results supported that *tin1* was under parallel selection across different cereals including foxtail millet, sorghum, and rice.

### Maize *tin1* splice-site variant originated from teosinte

In this study, we identified a splice-site variant from G/GT to C/GT in the intron of the 5′ UTR of *tin1*. The splice-site variant enhanced intron retention and might increase transcript of *tin1*, resulting in more tillers. Our large sequence analysis of maize *tin1* identified 46 maize inbred lines and seven teosinte lines containing the splice-site variant C/GT. Phylogenetic analysis revealed that all maize lines and teosinte lines with the splice-site variant C/GT were clustered into the same group (Fig. 6f and Supplementary Fig. 18). To our surprise, all the maize inbred lines with the splice-site variant C/GT were grouped together with one teosinte line (TEO86). This result signified that the splice-site variant C/GT in maize *tin1* was derived from a standing variation in the wild progenitor, teosinte.

## Discussion

**Fig. 7:**
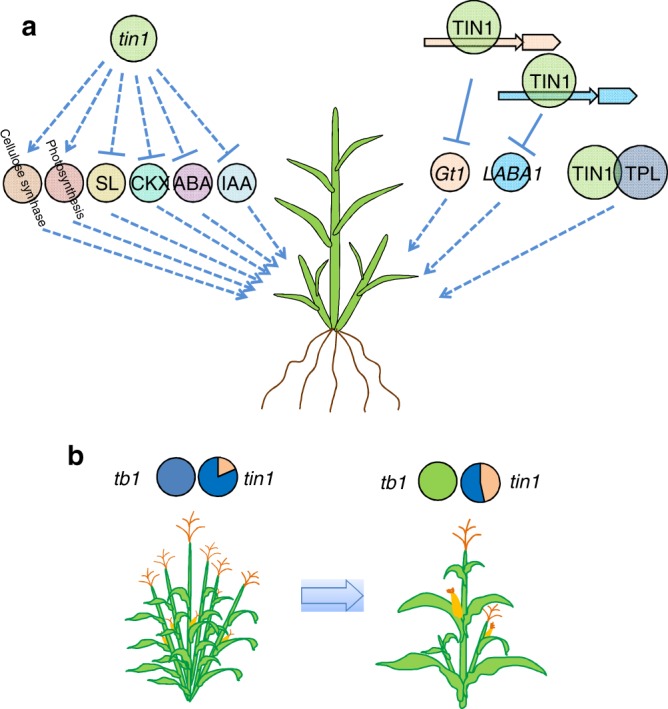
Maize *tin1* potential network and the path to tillering retention. **a** potential regulatory network mediated by *tin1*. Dashed arrows represented indirect promotion of genes. Solid and dashed T bars signified direct and indirect repression of genes. **b** The path to maize tillering retention. Sweet maize and popcorn retained tillering by picking up the standing splice-site variant C/GT in *tin1*, which originated from the wild progenitor, teosinte, after the key gene *tb1* became fixed in maize. The pie charts showed the percentage of causal variants in maize subgroups from two key genes, *tin1* and *tb1*.

The *tin1* functions as a transcription factor independently of the key gene, *tb1*, and controls tiller bud outgrowth. In this study, both DEGs from RNA-seq at the early and late stages of tiller bud development were most significantly enriched in response to light (Supplementary Data 5). In transient assays, a key shade-avoidance response gene *gt1* was directly targeted by *tin1* (Fig. 5h, i), indicating that *tin1* might be involved in light stimulus during tiller development. Maize *tin1* might respond to light stimuli and repress the expression of the marker gene of bud dormancy, *ZmDRM1* (Fig. 5c). The elongation of the tiller bud was then activated. Maize *tin1* regulated at least 10 tiller number-related genes during tiller bud outgrowth that are involved in multiple types of plant hormones, including D14^30^ and D3^31^ for the strigolactone receptors, two genes for auxin, three genes for cytokinin oxidase (CKX), one gene for ABA (Fig. 5g). *tin1* stimulates photosynthesis to accelerate tiller growth in both stages (Fig. 5g), and the expressions of two genes that increase leaf chlorophyll content and tiller number in rice, *pgl10*, and *ygl8*, were elevated (Fig. 5g). Similarly, the transcripts of four cellulose synthase genes^32–34^ are also upregulated (Fig. 5f), which is consistent with their function in promoting tiller development in rice. Meanwhile, maize TIN1 interacted with three TOPLESS related proteins and then controlled the downstream targets (Fig. 5j). Maize *tin1* regulates this complex regulatory network to maintain tiller outgrowth and to finally determine tiller number in maize (Fig. 7a).

The key gene, *tb1*, mainly contributed to maize single stalk during domestication. The expression of *tb1* was evaluated to repress the outgrowth of tiller bud in the domesticated maize. And *tb1* positively regulates the transcriptions of *gt1* and *tru1*^7,8^. In sweet corn, tiller bud can generally grow out under high expression of *tb1* (Fig. 5a). Transcript analysis revealed that *tin1* might work independently of *tb1* (Fig. 5a, b). RNA-seq analysis showed that *tin1* repressed the expression of *gt1* and did not change the expression of *tru1*. Further gene network revealed that *tin1* was involved in multiple pathways including cellulose synthesis, photosynthesis, and hormone responses. Although some pathways including *gt1* might be shared between *tin1* and *tb1*, other specific pathways downstream the *tin1* gene might overcome the effect derived from *tb1* and control tiller buds to grow out despite high expression of *tb1*. Our QTL analysis showed that a QTL for tiller number was placed in the end of the long arm of chromosome 4. While *sugary 1* was located on the short arm on chromosome 4, which is far away from this tiller number QTL (Supplementary Fig. 1d, e). This result suggested that *sugary 1* was not involved in the development of tiller. All these results signified that *tin1* is an important gene for plant architecture in maize.

Comparative genomic analysis of *tin1* revealed that a syntenic block harboring four chromosomal fragments on maize chromosome 7, rice chromosome 7, foxtail millet chromosome 2 and sorghum chromosome 2. The *tin1* locus contained eight, six and six *tin1* duplicated copies in rice, foxtail millet, and sorghum, respectively. In maize, only one *tin1* copy was identified based on the domesticated maize genome. Whether wild progenitor of maize, teosinte, has multiple *tin1* duplicated copies similar to other grass still needs to be explored.

A parallel transition during domestication is from the loose plant architecture with more tiller number in wild progenitors to the compact plant architecture with less tiller number in domesticated cereals. In this study, we combined QTL mapping, association mapping and comparative mapping to identify that *tin1* is responsible for this parallel phenotypic change during domestication. Our result provided a good case that parallel phenotypic changes across different cereals during domestication or diversification might share similar genetic basis. A large deletion with one and a half *tin1* copies was present during domestication in foxtail millet. Such large deletion was also present in rice *tin1* locus.

Some types of maize, including sweet corn and popcorn retained the tillering habit during maize diversification. The reason for this is most likely due to changes in plant architecture, in which a plant with a main stalk plus two or three tillers will bear more ears per plant, with potential yield increases. Previous studies showed that removal of tiller decreased the production of sweet maize^35,36^. In this study, we overexpressed *tin1* in B37, a no-tillering maize, and both tiller number and ear number were significantly increased in the transgenic plants compared with nontransgenic plants (Fig. 4a–g). As sweet corn is a typically consumed fresh corn, the ears on the main stalk and tillers are generally well developed. Thus additional ears on the tillers will generally increase the total yield of a sweet maize plant. For popcorn, generally bearing smaller ears, more tillers will likely also enhance ear number and yield.

The key gene, *tb1*, mainly contributes to the transition from multiple tillers in teosinte to a single stalk in maize. A transposon insertion in the regulatory region of the *tb1* promoter leads to a loss of tillering habit in most domesticated maize^3^. The *tin1* functions independently of *tb1* in the regulation of maize tillering. All the domesticated maize lines studied here, including sweet corn and popcorn, contained the causal variant of transposon in the promoter of *tb1* (Fig. 7b, Supplementary Data 8 and 9). The frequency of the causal splice-site variant of *tin1* was 18.4, 16.3, and 47.1% in teosinte, domesticated maize excluding sweet corn and popcorn, and sweet corn and popcorn, respectively (Fig. 7b, Supplementary Data 8 and 9). The high allele frequency of this causal variant in sweet/popcorn might be due to random genetic drift because no human selection was present in maize *tin1*. Phylogenetic tree based on genome-wide SNPs revealed that sweet maize and popcorn seemed to be early domesticated types (Supplementary Fig. 19). These results indicated that the splice-site variant of *tin1* might be retained after *tb1* became fixed in maize during diversification.

## Methods

### Plant materials

A RIL (F_5_) population of 232 lines was constructed between a maize elite inbred line B37 and a typical sweet maize line P51 (Supplementary Fig. 1). Each line of F_5_ populations of 12 plants was grown in a 50 × 300-cm plot at the experimental stations of the China Agricultural University in Beijing. A global inbred maize population (Supplementary Data 8) of 263 lines^37^ was grown in a randomized block design with three replicates in Beijing in 2017 for phenotyping. All foxtail millet accessions from different regions of the world were obtained from the Germplasm Resources Information Network (http://www.ars-grin.gov/) and the Chinese Crop Germplasm Resources Information System (http://www.cgris.net) (Supplementary Data 10). All plant materials for fine mapping in this study were grown in Beijing or Hainan between 2015 and 2018.

### QTL mapping

We genotyped the RIL population with 232 lines by 212 SSR markers evenly distributed on the 10 maize chromosomes. Then a genetic map was constructed, which spanned 2221.28 centimorgan (cM), and contained an average genetic distance of 10.48 cM between pairs of neighboring SNPs.

The phenotypic data and genetic map were input into R/qtl^38^ to detect QTLs, using multiple-QTL mapping method. Initial simple interval mapping was performed with the R/qtl function scanone using the Haley–Knott regression method, and a significance threshold at the *P* = 0.05 level for each trait was determined through 1000 permutations. Next, the positions of the QTLs with logarithm of the odds (LOD) scores above threshold were refined using the R/qtl function refineqtl. Subsequently, additional QTLs were scanned with the function addqtl based on the refined QTLs. Once an additional significant QTL with LOD score above threshold was detected, the new QTL was added into the model and the positions of all the QTLs were refined again. All these above steps would be circulated until no significant QTL was added. When all the QTL positions had been refined, the genetic effect and the significance of each QTL were detected with drop-one-QTL analysis in the full model.

The two NILs were derived from the self-pollination of a RHL (F_6_), harboring a heterozygous genomic fragment at *tin1* and homozygous genotypes at most other loci (Supplementary Fig. 1d, e).

### Fine mapping of *tin1*

A large population (F_7_) of over 10,704 individuals (Supplementary Fig. 2) was constructed to fine map *tin1*, derived from one RHL (F_5_) with a heterozygous genomic fragment at *tin1*. Marker screening using 10 SSRs and one SNP identified 13 representative recombination types (Supplementary Fig. 2) in this population. The progeny populations derived from these recombinant plants, all harboring heterozygous/homozygous fragments in the *tin1* target region, were used to test the correlation between genotypes and tiller number. The correlations between genotypes and tiller number were determined with a linear regression model, to identify whether the target QTL is present or absent in the heterozygous fragment of the *tin1* target region in the recombination plants^39^. A significant *P*-value (*F*-test) placed the target QTL in the heterozygous fragments. If the *P*-value was not significant, the target QTL was then mapped within the homozygous fragments. On the basis of this modified progeny test^39^, *tin1* was finally placed between two markers: S2 and S3 (Supplementary Fig. 2). All primers used in the fine-mapping process are listed in Supplementary Data 11.

### Plant transformation

Two overexpression constructs consisting of maize *tin1* from both no-tillering parent B37 and the tillering parent P51, under the control of the ubiquitin promoter, were introduced into B73 with a protocol conducted in HiII^40^. Three and one overexpression (T_0_) events were obtained for *tin1-B37* and *tin1-P51*, respectively. All four T_0_ transgenic plants were backcrossed to the no-tillering parent B37 and self-pollinated for two generations, then phenotyped in Beijing. The BAR gene was amplified to distinguish the transgenic and nontransgenic plants.

### Protoplast transient expression assay

Transient assays in maize leaf protoplasts were used to test the effects on gene expression of all the variations in the promoter region of *tin1*. Briefly, a B37 3.1-kb promoter sequence, which contains the whole fine-mapping interval from marker S3 to the 5′ UTR, (B37-Pro::LUC) and the corresponding P51 2.8-kb promoter (P51–Pro::LUC) were inserted into the LUC vector (pGreenII 0800-LUC), which contained a Renilla reniformis reporter gene (REN) controlled by the cauliflower mosaic virus (CaMV) 35S promoter and a firefly luciferase reporter gene (LUC) controlled by a custom promoter. These two constructs were then introduced into seedling-stage etiolated maize B73 mesophyll protoplasts. 20 μg reporter construct was next mixed with the newly isolated protoplasts in PEG transfer solution for 18 min on ice. The transformed protoplasts were incubated for 18 h at 25 °C and then harvested. After harvest, the protoplasts were lysed with Passive Lysis Buffer (PLB, Promega) and assayed with the Dual-Luciferase Reporter Assay System (Promega). Six biological replicates were conducted for each construct and all experiments were repeated three times.

To examine the impact of TIN1 on the expression of eight candidate target genes, we performed a dual-luciferase transient expression assay in maize NIL-P51 leaf protoplasts. The coding sequence of *tin1* was cloned into the pGreenII 62-SK vector under the control of the 35S promoter to generate the effector construct. For the reporter construct, a 2.0-kb fragment of the eight gene promoters from NIL-B37 or NIL-P51 was cloned into pGreenII 0800-LUC to drive the firefly luciferase (LUC) gene. The reporter construct and the *tin1* effector were co-transformed into maize leaf protoplasts. The corresponding reporter and the empty effector construct were used as a control.

### 5′ and 3′ RACE

Total RNA was extracted from the tiller buds of the *Tin1/tin1* NIL plants at 25 DAP, treated with the RNase-free DNase I (Takara) and purified using the RNAclean kit (Tiangen). 5′ and 3′ RACE were carried out using the SMART RACE cDNA Amplification Kit (Clontech) following the manufacturer’s instructions.

### Association mapping

To perform association mapping tests for maize *tin1*, the variants in a 1-kb fragment from position 6,104,752 to 6,105,752 on chromosome 7 (V4) were extracted from the whole-genome sequencing data of the maize 282 association panel (Supplementary Data 8)^41^. The 1-kb fragment consisted of an ~197-bp promoter fragment, a 221-bp 5′UTR, and the 582-bp open reading frame (ORF) region and 48 SNPs with a frequency of over 0.05 were identified. A mixed linear model was applied to conduct association mapping testing in TASSEL5^42^. The significance threshold was corrected for multiple testing through Bonferroni correction based on the following equation: α′ ≈ *α*/*n* = 0.001, where *α* is the nominal significance threshold (*α* = 0.05) and *n* is the number of variants (*n* = 48).

To test the *tin1* gene function in foxtail millet, the association test was performed in TASSEL5 using a simple linear model in a global foxtail millet population (Supplementary Data 10) with 24 wild and 65 domesticated foxtail millet accessions from around the world. The significance threshold was corrected for multiple testing through Bonferroni correction based on the following equation: α′ ≈ *α*/*n* = 6.58 × 10^−4^, where α is the nominal significance threshold (*α* *=* 0.05) and n is the number of variants (*n* = 76).

### Subcellular localization

The *tin1* CDS was fused with GFP to construct the *Ubi*:: TIN1-GFP vector under the control of the maize *Ubiquitin* promoter. The *Ubi*::TIN1-GFP construct was first transformed into maize B73 leaf protoplasts and onion epidermal cells, and then the subcellular localization of GFP signals was investigated with a Nikon C1 confocal laser microscope.

### Real-time RT-PCR

Total RNAs from the tissues including tassel, ear, leaf, auricle, root, IM, SAM and tiller bud (2 mm and 5 cm) from the NIL plants were prepared from 3–5 plants using an RNA Extraction Kit (TianGen Biotech). First-strand cDNA was synthesized from 1 μg total RNA with TransScript-Uni cDNA Synthesis SuperMix (TransGen Biotech). Using an internal control of the house-keeping *GADPH1* gene, qPCR with three technical replicates and three biological replicates was conducted on an ABI7500 thermocycler. The relative expression levels were calculated with the *ΔΔ*CT (DDCT) relative quantification method^43^.

### RNA-seq analysis

RNA samples with three biological replicates were collected from the tiller buds of the *tin1/Tin1* NIL plants at 25 DAP and 30 DAP. The 12 DNA-free RNA samples were then sequenced with a HiSeq-2500 and 80 Gb of raw sequencing data were obtained. These raw RNA-seq reads were then analyzed with a common RNA-seq pipeline^44^. Finally, genes that were differentially expressed (DE) between these NILs were identified based on their corrected *P*-values.

### Yeast two-hybrid assay

Full-length coding sequences of *tin1*, and N-terminal (1–84 aa) and C-terminal (84–192 aa) deletions of *tin1* were cloned into pGBKT7 (bait vector), namely BD-TIN1, BD-TIN1-∆N and BD-TIN1-∆C. Full-length coding sequences of ZmTPL1, ZmTPL2, ZmTPL3 were cloned into pGADT7 (prey vector), namely AD-ZmTPL1, AD-ZmTPL2 and AD-ZmTPL3. The bait and prey vectors were then transformed into the yeast strain AH109 following the instructions for the MatchmakerTM GAL4 Two-Hybrid System 3 and Libraries (Clontech, USA). Protein interactions were assayed on selective medium lacking Leu, Trp, His, and Ade.

### Comparative mapping

Pairwise genomic comparison was conducted with SYNMAP in CoGe (http://genomevolution.org/CoGe/). The syntenic map was generated based on these data sets in CoGe, including maize (B73, id333), rice (Nipponbare, id3), foxtail millet (Yugu1, id32546) and sorghum (Tx623, id331).

### DNA diversity analysis

To analysis DNA diversity, maize 1.5-kb *tin1* sequences including a 702-bp promoter and a 798-bp 5′-UTR and CDS fragment in another global maize population with 124 inbred lines (from 282 association panel), 45 landrace, and 37 teosinte accessions (Supplementary Data 9) were used. In foxtail millet, a 1184-bp genomic segment, flanking the 18-kb deletion break point and containing a forward 695-bp and a downward 489-bp sequences, was sequenced in the 89 foxtail millet accessions (Supplementary Data 10). The resulting PCR products were cleaned using the QIAquick PCR Purification Kit (Qiagen), then sequenced using ABI 3730.

*Tin1* sequences were imported into ClustalW to construct a nucleotide alignment matrix, which was further used for nucleotide diversity (*π*) analysis by DnaSPV5.1^45^. Tajima’s *D* tests were performed by DnaSPV5.1. Protein sequences of *tin1* orthologs from rice, foxtail millet, and sorghum (Supplementary Fig. 14) were sourced from the National Center for Biotechnology Information (http://www.ncbi.nlm.nih.gov/) and Phytozome (http://www.phytozome.net) databases. The protein alignment and phylogenetic tree was analyzed using MEGA7^46^.

Maize *tin1* nucleotide alignment matrix based on 1.5-kb *tin1* sequences was also imported into MEGA7 to generate a phylogenetic tree using a statistical method of maximum likelihood under the Tamura-Nei model.

For genome-wide selection tests for maize *tin1*, SNP data from maize haplotype version 3 (HapMap 3)^41^ was used. The SNPs with a missing data ratio of >10% were filtered. The maize contained a subset of maize accessions for HapMap 2^47^, which included 60 common maize, 15 sweet/popcorn and 19 teosinte accessions. Genetic diversity was firstly compared between teosinte and sweet/popcorn, and common maize and sweet/popcorn^48,49^. Briefly, *F*st and *π* values were calculated with a 10-kb adjacent nonoverlapping window through a 500-bp step using VCFtools^50^. XP-CLR^29^ analyses for selective tests between teosinte and sweet/popcorn, and common maize and sweet/popcorn, were then calculated using a nonoverlapping window size of 10 kb with a 500-bp step. The average genetic distances for Chromosomes 1 to 10 were estimated to be 0.69, 0.68, 0.70, 0.63, 0.72, 0.66, 0.78, 0.78, 0.84, and 0.75 cM/Mb, respectively, using the information from previous studies^51,52^. For each analysis, the regions with *π* ratio, *F*st and XP-CLR values in the top 5% genome-wide distribution were determined as selective sweeps.

### Trait comparisons in near isogenic lines

Plants from the F_6_ family that was heterozygous at the *tin1* locus were screened, using a *tin1* flanking SSR marker P4 and S3, to identify plants that harbored either homozygous *tin1/tin1* (NIL-B37) or homozygous *Tin1/Tin1* (NIL-P51) alleles. It was possible to use homozygous plants with either *tin1/tin1* or *Tin1/Tin1* alleles as near isogenic lines (NILs), as most genomic regions were homozygous in the F_6_ generation. Using the Student’s *t*-test (*n* > 30), trait comparisons between the pair of NILs were performed for11 traits, including tiller angle, plant height, ear height, tassel length, tassel branch number, leaf length, leaf width, leaf angle, leaf number above ear, kernel row number and ear number on main stalk (Supplementary Fig. 20).

### mRNA stability measurements

Briefly, the tiller buds (1–2 mm) of the NIL-B37 and NIL-P51 at 25 DAP were harvested and incubated in actinomycin D (50 μg ml^−1^) for 0, 0.5, 1, or 2 h at room temperature. The solution was changed every 0.5 h. All samples were collected and immediately frozen in liquid nitrogen. The tissues were stored at −80 °C or subjected to total RNA extraction. Samples were performed in three biological replicates with three technical replicates.

### Reporting summary

Further information on research design is available in the Nature Research Reporting Summary linked to this article.

## Supplementary information

Supplementary Information

Peer Review File

Reporting Summary

Description of Additional Supplementary Files

Supplementary Data 1

Supplementary Data 2

Supplementary Data 3

Supplementary Data 4

Supplementary Data 5

Supplementary Data 6

Supplementary Data 7

Supplementary Data 8

Supplementary Data 9

Supplementary Data 10

Supplementary Data 11

## Data Availability

Data supporting the findings of this work are available within the paper and its Supplementary Information files. A reporting summary for this Article is available as a Supplementary Information file. The datasets generated and analyzed during the current study are available from the corresponding author upon request. RNA-seq data was deposited in in the National Center for Biotechnology Information (NCBI) under the SRA accession number PRJNA549063. The source data underlying Figs. 2i, j, 3d, 4e–g, i, 5a, b, and i, as well as Supplementary Figs. 3, 8, 9, 11, 12, 13, and 20 are provided as a Source Data file.

## Source Data

Source Data
