# Supplementary material for: The tin1 gene retains the function of promoting tillering in maize
Source: Nat Commun. 2019 Dec 6;10:5608. doi: 10.1038/s41467-019-13425-6 (PMC6898233; doi:10.1038/s41467-019-13425-6)
Supplement: Supplementary file 1 — Supplementary Information [file 41467_2019_13425_MOESM1_ESM.pdf]

The *tin1* gene retains the function of promoting tillering in maize

Zhang *et al.*

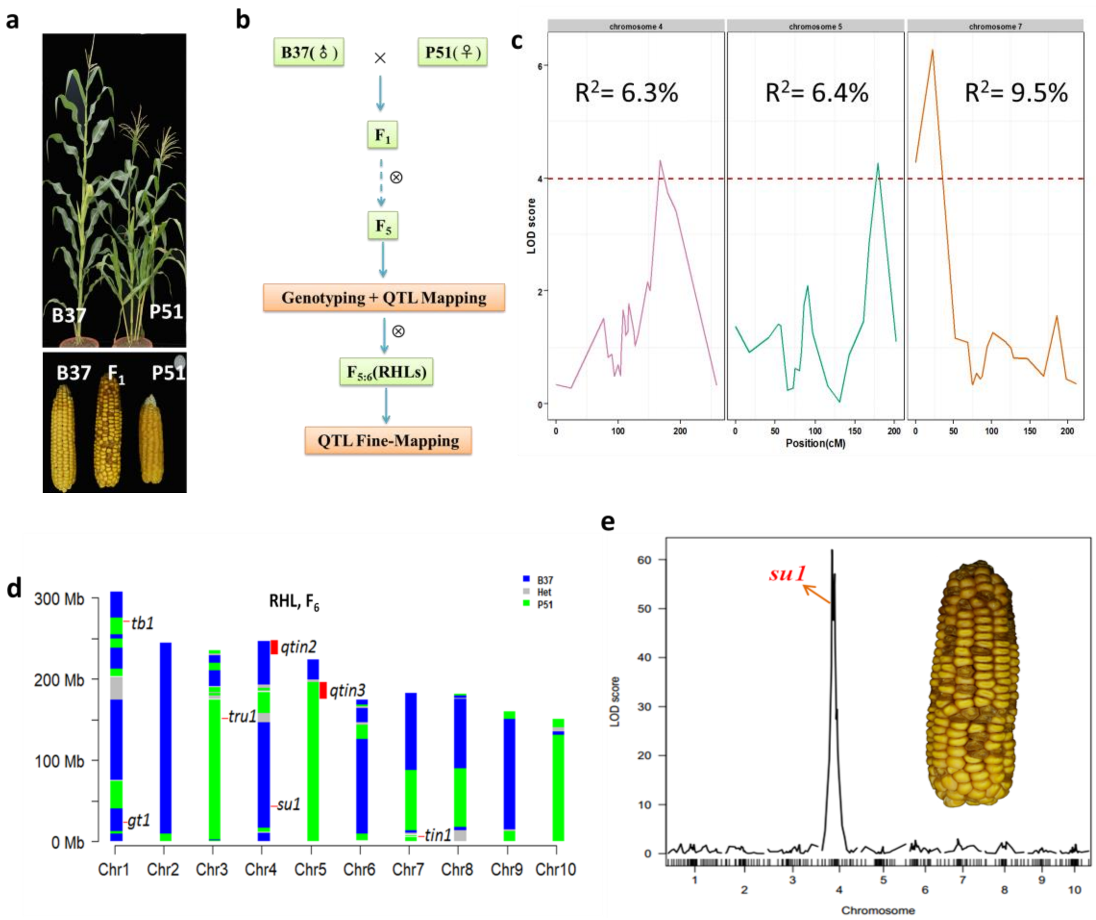

Supplementary Figure 1. QTL mapping in the RIL populations derived from the crosses between B37 and P51 and a residual heterozygous line to generate NILs.

(**a**) A typical sweet maize P51 with multiple tillers was crossed with an elite inbred line B37 with a single stalk. (**b**) A RIL population (F_5) was constructed and residual heterozygous lines (RHLs) were selected in the generation of $F_{5:6}$ for QTL fine mapping. (**c**) QTL mapping identified three QTLs for tiller number on chromosome 4, 5 and 7. (**d**) Genotype of a RHL to generate NILs based on SNPs from RNA-seq. All the seven loci were homozygous except for tin1. (**e**) QTL mapping revealed that *sugary1* was located in the short arm of chromosome 4.

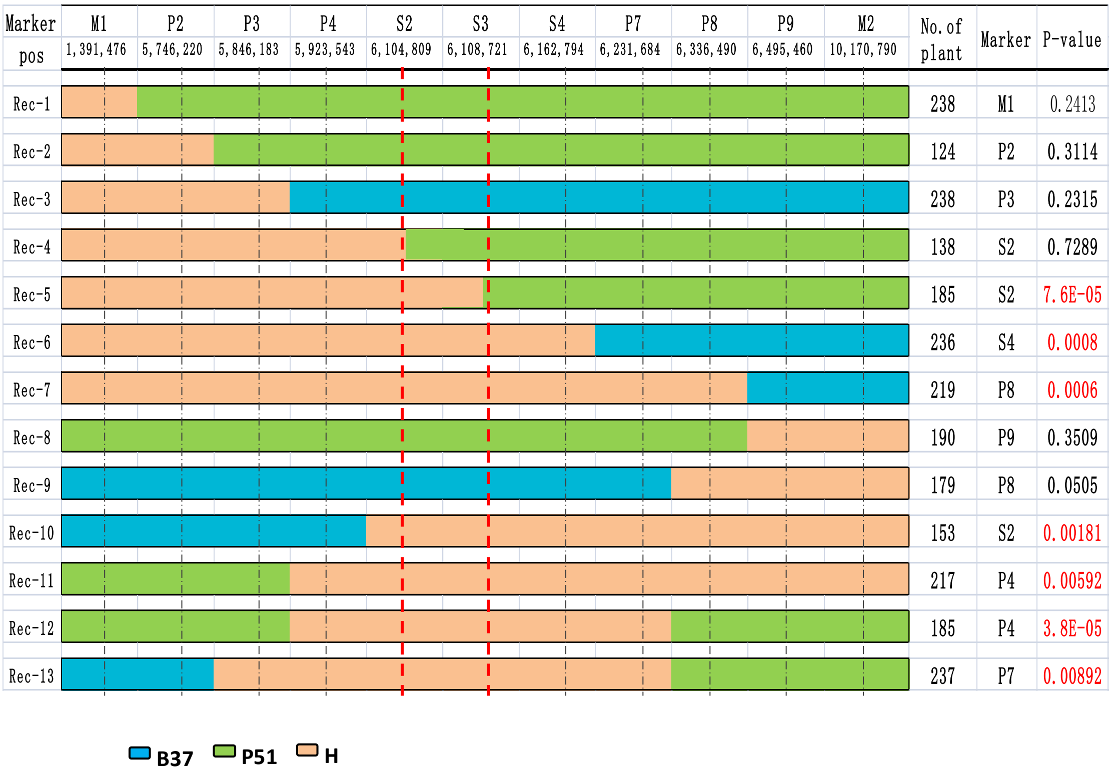

Supplementary Figure 2. Fine mapping of *tin1*.

11 molecular markers were developed to fine-map maize *tin1* in a large population with 10,704 individuals. In the descendent population with 138 individuals (F_8) derived from the recombination plant Rec-4, the marker S2 was non-significantly correlated with tiller number (P=0.7289), indicating that *tin1* was placed on the right side of S2 (in the homozygous fragment). The marker S3 was significantly (P=7.6×10^{-5}) correlated with tiller number in the progeny population (F_8) with 185 plants derived from the recombination plant Rec-5, signifying that *tin1* was placed on the left side of S3 (in the heterozygous region). This fine-mapping region was next confirmed in the F_9 generation applying progeny tests with Student' s t-test (Figure 3 in the main text). The *tin1* was then finally narrowed down to a region of 3.9 kb, flanked by S2 and S3. Pos, position. Blue, green and orange bars represented homozygous chromosomal fragments from B37 and P51 and heterozygous chromosomal segments, respectively. Significant signals were highlighted in red.

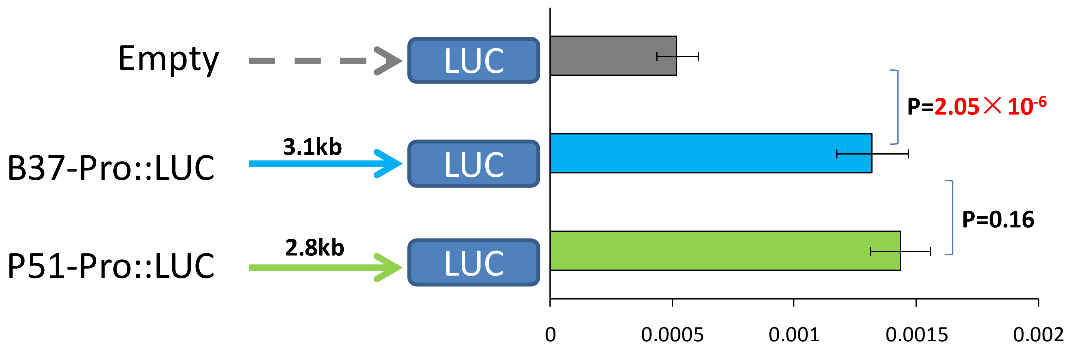

Supplementary Figure 3. Luciferase transient assays.
The promoter fragments from two parental lines B37 and P51 in the fine-mapping region (*B37-Pro::LUC* and *P51-Pro::LUC*) were introduced into the Luciferase vector. Relative expression was quantified based on the LUC/REN ratio, and empty vector was used as a control. Both *B37-Pro::LUC* and *P51-Pro::LUC* showed significantly higher expressions than that of the control with empty vector. By contrast, no significantly changed expressions were present between *B37-Pro::LUC* and *P51-Pro::LUC*. Error bar, SD (n=6). Source data are provided as a Source Data file.

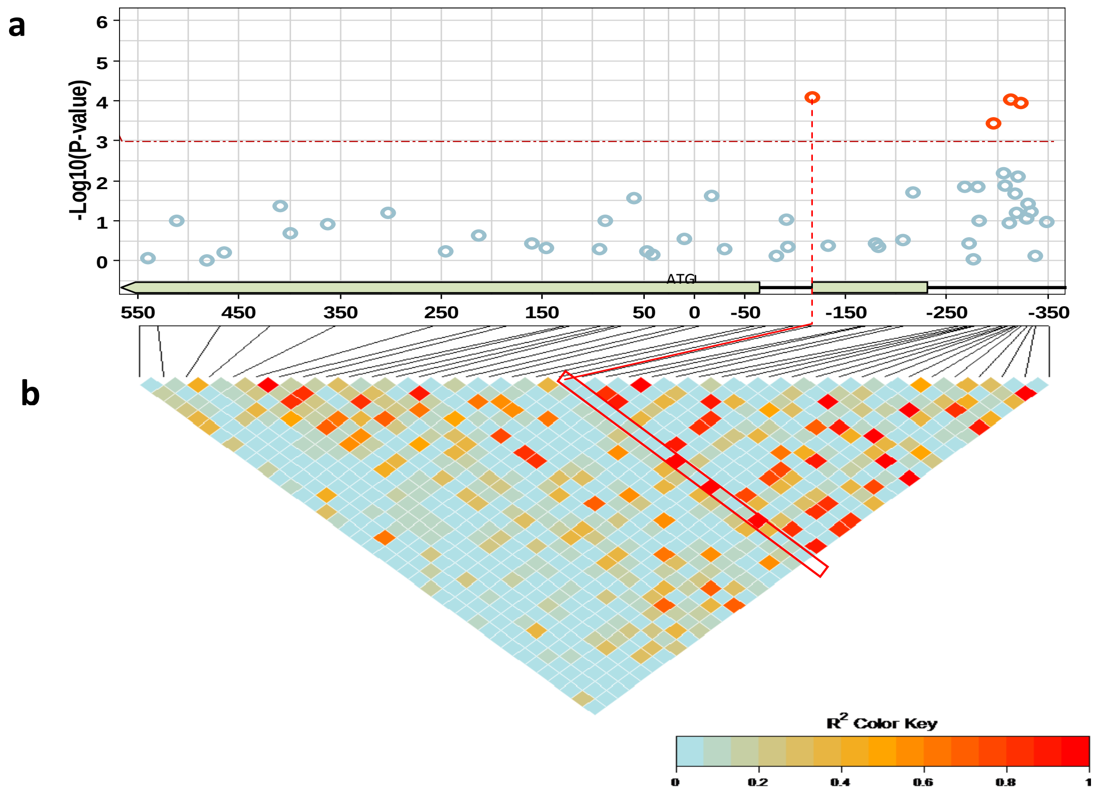

Supplementary Figure 4. Association mapping and linkage disequilibrium analysis.

(**a**)Association mapping revealed that four variants (in red) were highly associated with tiller number in a natural maize population. The red dashed line signified the 5 % significance threshold with Bonferroni correction for 48 tests. The gene structure of *tin1* was shown on the x-axis. The start codon was regarded as position "0". (**b**) Linkage disequilibrium (LD) heat map. The LD between the splicing variant from G/GT to C/GT and the other three variants with strong association signals were highlighted in the red box. The LD scores between the splicing variant and the other three variants were over 0.95. The R^2 color key was shown at the bottom.

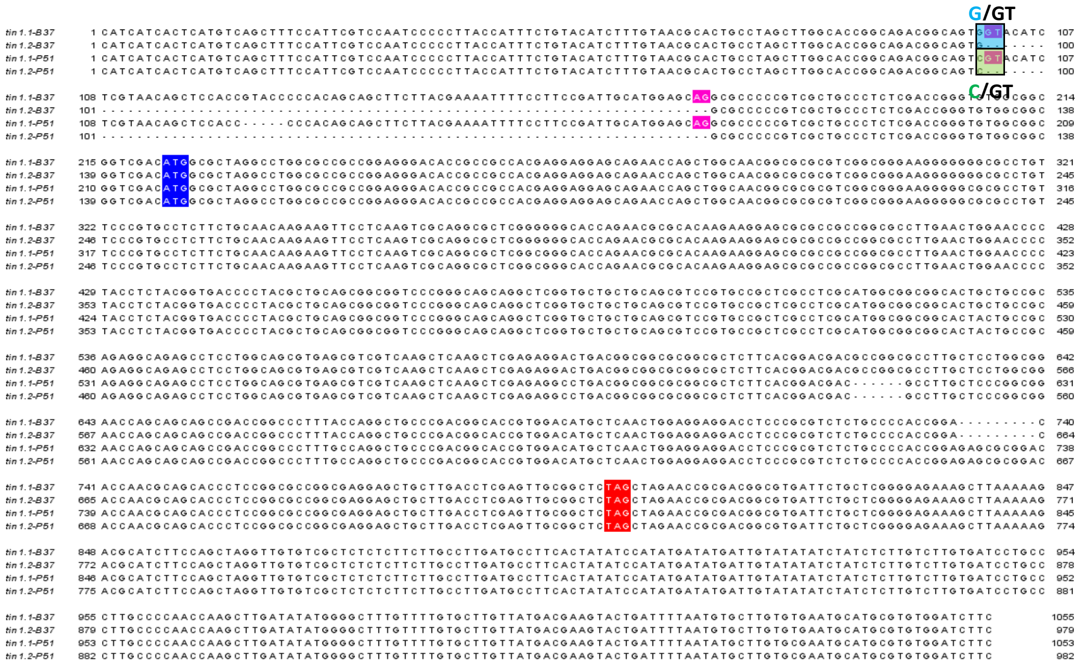

Supplementary Figure 5. 5’RACE and 3’ RACE analysis. The splice-site variant from G/GT to C/GT and the start and stop codons of *tin1* were shown.

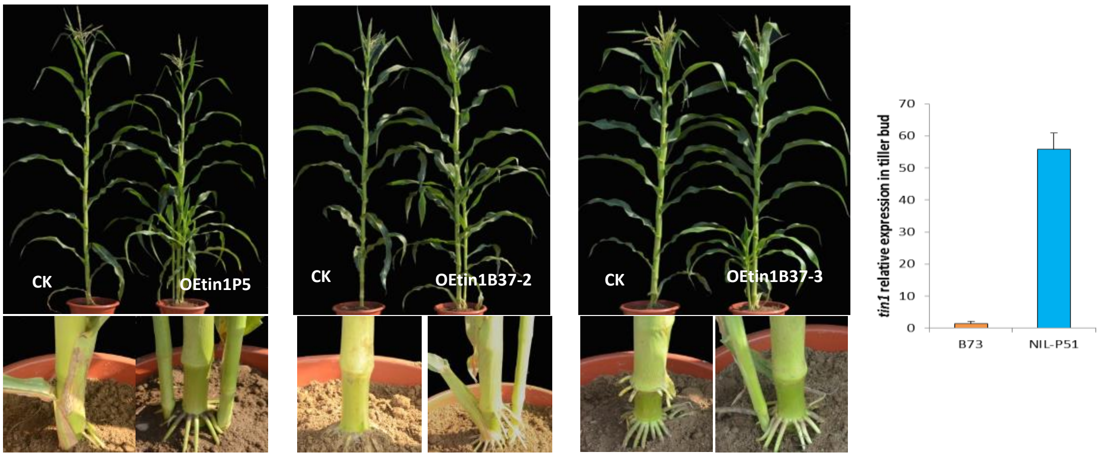

Supplementary Figure 6. Transformation analysis.
Overexpression of *tin1* genes from the two parental lines P51 and B37 (*OEtin1P51*, *OEtin1B37-2* and *OEtin1B37-3*) significantly increased tiller number compared with the control plants (CKs). The whole plants were shown at the top, and a close-up view of the tiller base was shown at the bottom. The expressions of *tin1* were also shown for maize inbred lines B73 and P51 based on real-time qPCRs. Error bar, SD (n=3).

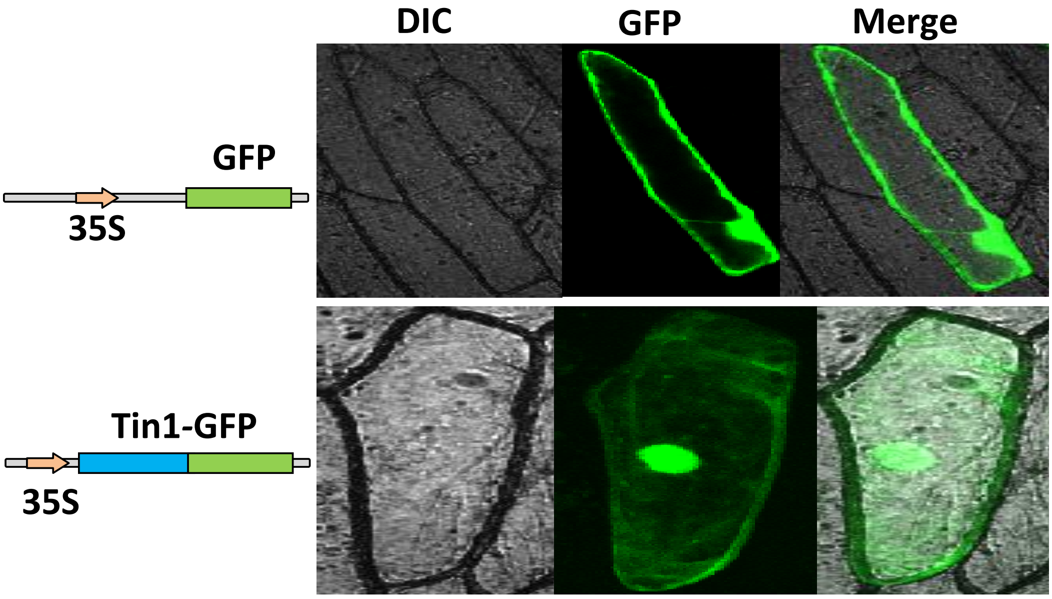

Supplementary Figure 7. TIN1-GFP fusion protein was expressed in onion epidermal cells.
The TIN1-GFP fusion protein was mainly expressed in the nucleus.

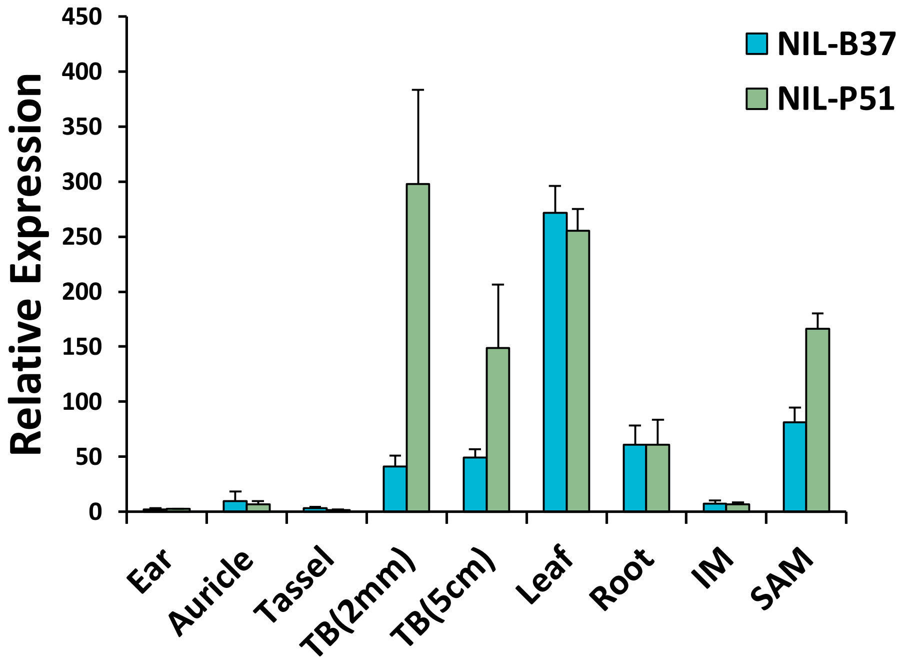

Supplementary Figure 8. Transcription analysis of *tin1* based on real time qPCRs.

Maize *tin1* was expressed in all the tissues tested. Blue and green bars represented NIL-B37 and NIL-P51. TB, tiller bud. Error bar, SD (n=5). Source data are provided as a Source Data file.

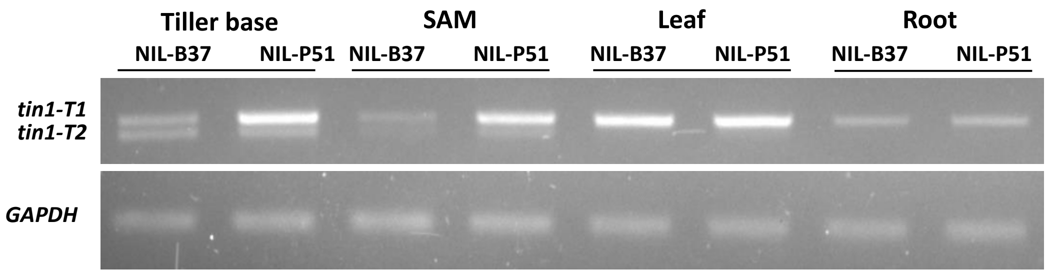

Supplementary Figure 9. RT-PCRs for the *tin1* gene in different tissues.

Source data are provided as a Source Data file.

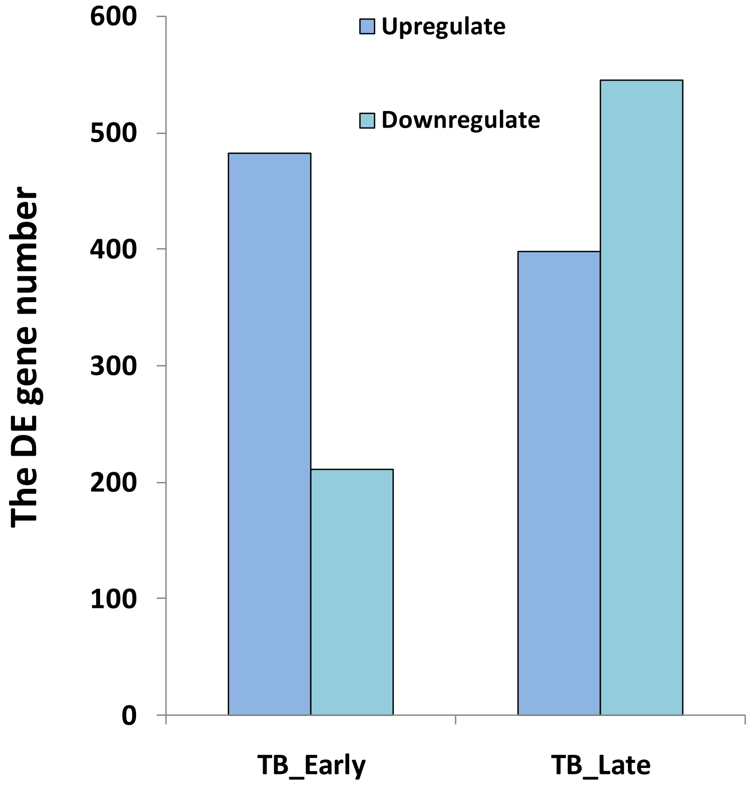

Supplementary Figure 10. The differentially expressed gene number based on RNA-seq.

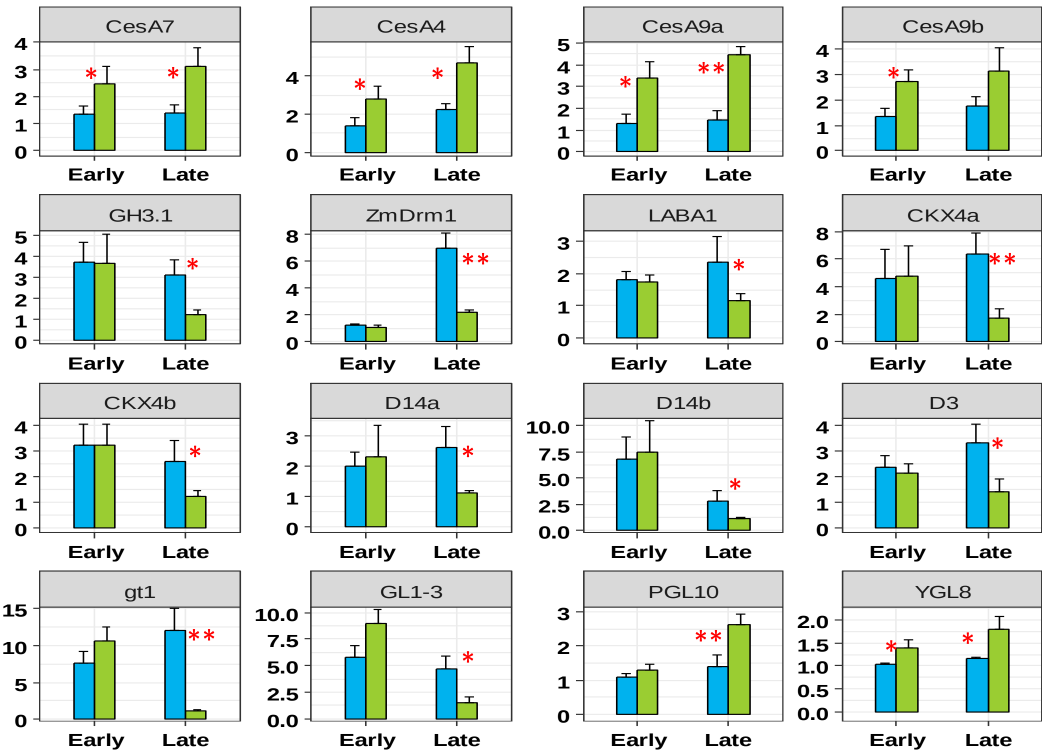

Supplementary Figure 11. Real-time qRT-PCRs for 16 differential expressed genes.

The transcript patterns for the 16 genes based on Real-time qRT-PCR are consistent with those based on RNA-seq. Single red and double red stars represented the significances with $P < 0.01$ and 0.001 (two-tailed Student's t-test). Error bar, SD (n=3). Source data are provided as a Source Data file.

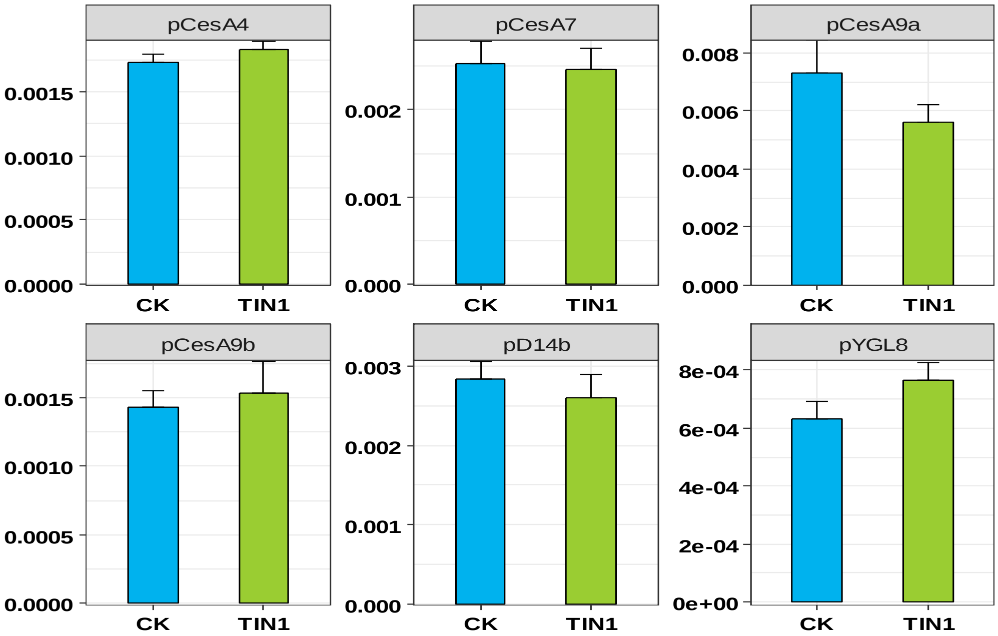

Supplementary Figure 12. Dual-luciferase transient expression assays for six tiller-related genes.

Overexpression of *tin1* (effector) did not significantly change the transcriptions of the target genes (reporters) in comparison to the control (CK) based on Student's t-tst. Error bar, SD (n=3). Source data are provided as a Source Data file.

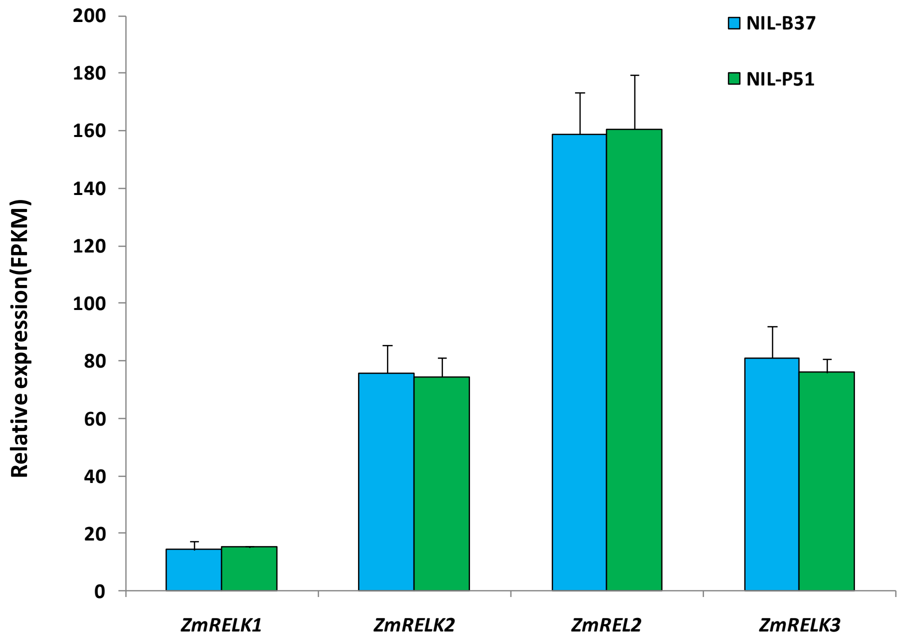

Supplementary Figure 13. Transcription patterns for the four TPL genes in maize tiller buds based on RNA-seq.
Error bar, SD (n=3). Source data are provided as a Source Data file.

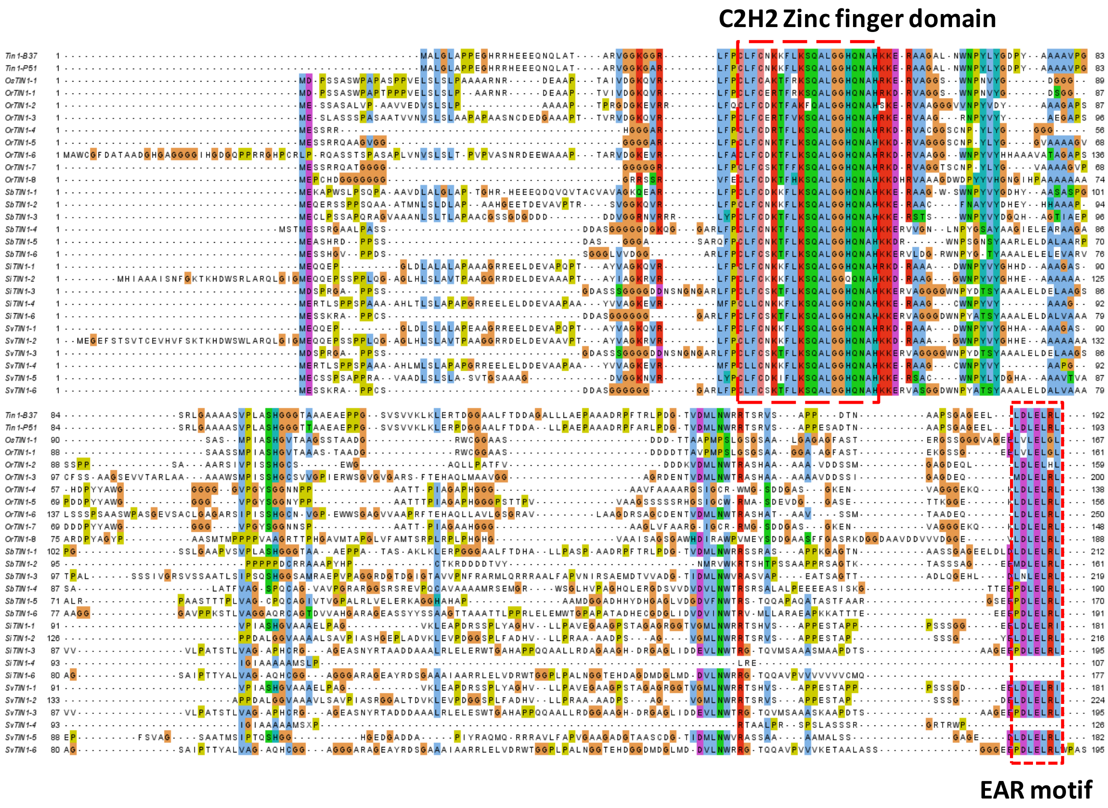

Supplementary Figure 14. TIN1 protein alignment among different species.

Alignment of 28 TIN1 proteins from maize, rice, foxtail millet and sorghum. All these TIN1 proteins were highly conserved. The C2H2 zinc finger domain and EAR motif in N- and C-termini were highlighted in red dashed line boxes.

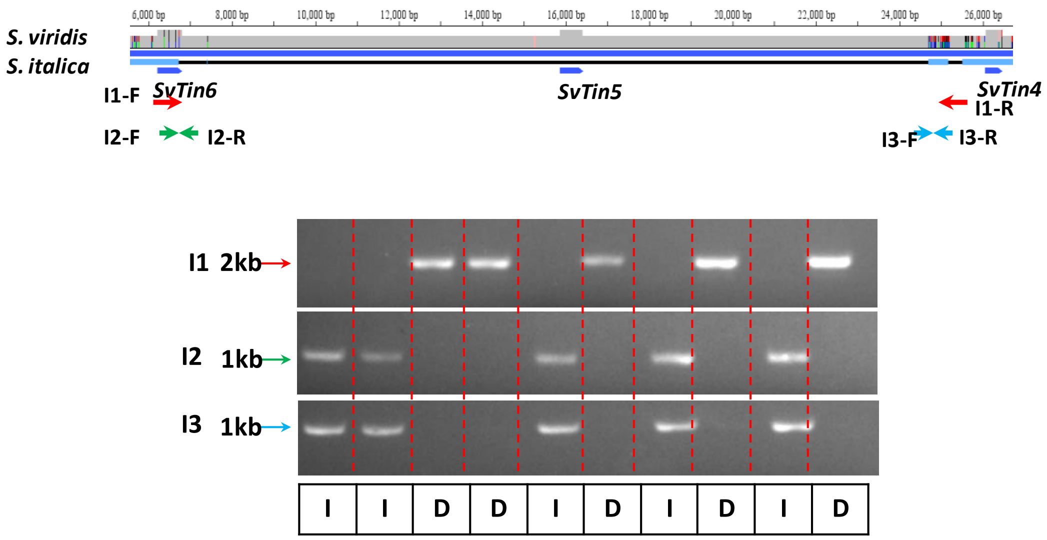

I: Homozygous genotype with the 18-kb insertion
D: Homozygous genotype without the 18-kb insertion

Supplementary Figure 15. Three pairs of primers were applied to differentiate the 18-kb insertion/deletion in foxtail millet. Three pairs of primers I1, I2 and I3 were used to amplify three fragments of 2 kb, 1 kb and 1kb. A homozygous line with the 18-kb fragment can only produce two 1-kb I2 and I3 fragments. While a homozygous line without the 18-kb sequences can only produce the 2-kb I1 segment. Three *tin1* copies were shown above.

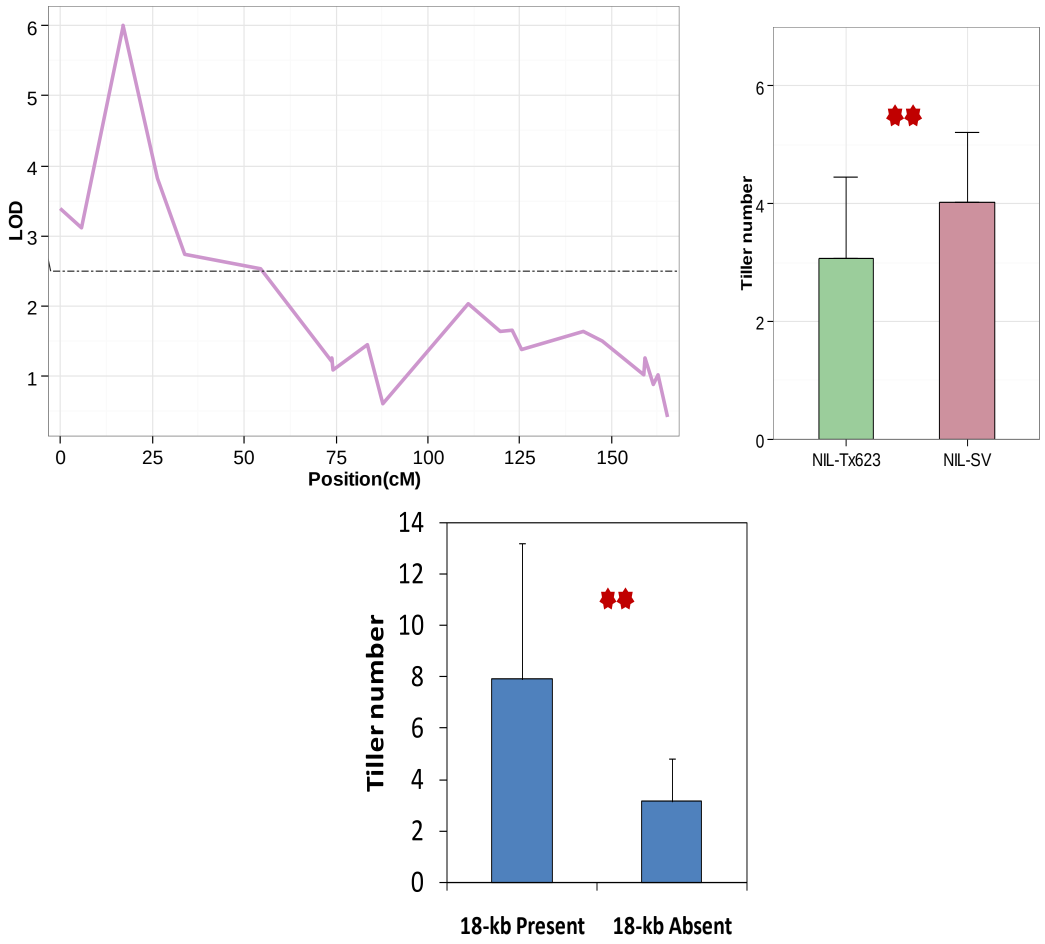

Supplementary Figure 16. QTL mapping identified a major QTL for tiller number in sorghum *tin1* region and genetic effect for *tin1* in foxtail millet.

QTL mapping in a recombinant inbred line (RIL) population, derived from a cross between a wild *Sorghum virgatum* (*SV*) and a domesticated sorghum Tx623, identified a major QTL of tiller number in sorghum *tin1* genomic region. This major QTL accounted for 6.5% of total phenotypic variation and the genetic effect reached one tiller per plant, estimated between two NIL-SV and NIL-tx623 in sorghum. The genetic effect of foxtail millet *tin1* reached 4.75 tillers in association mapping. Double red stars, strong significance (P<0.001, two-tailed Student's t-test). Error bar, SD (n=30).

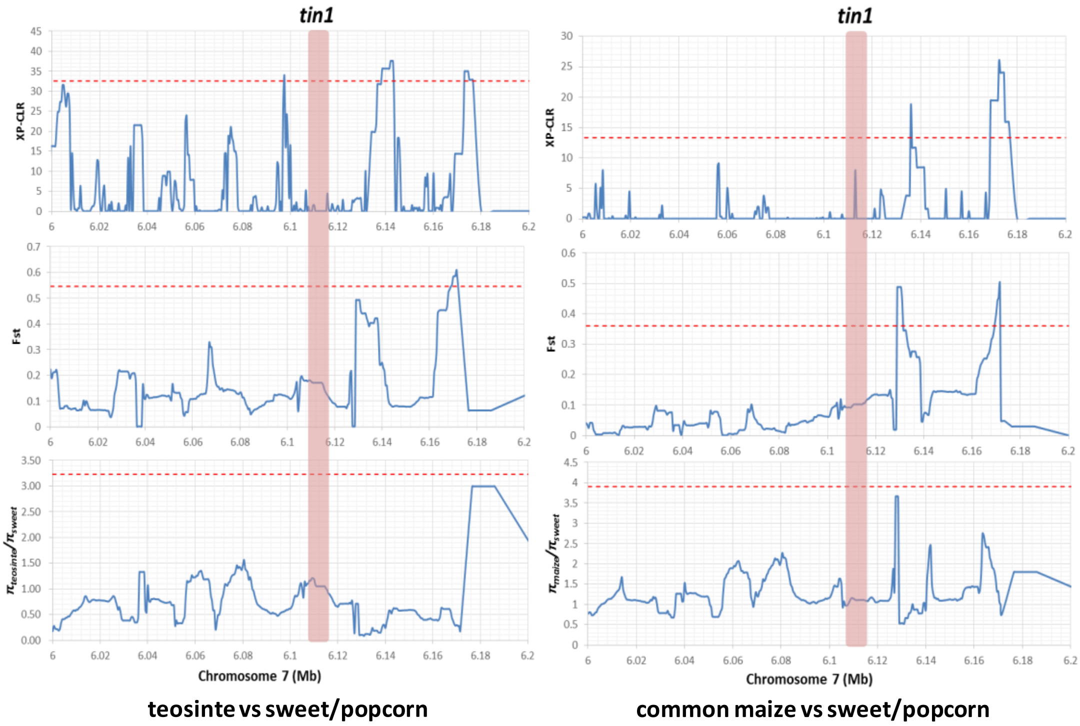

Supplementary Figure 17. Genome-wide selection analysis for maize *tin1.*

Genome-wide selection analysis between sweet/popcorn and common corn, and sweet/popcorn and teosinte. Red dashed line, 5% genome-wide threshold; pink box, the region of maize *tin1*.

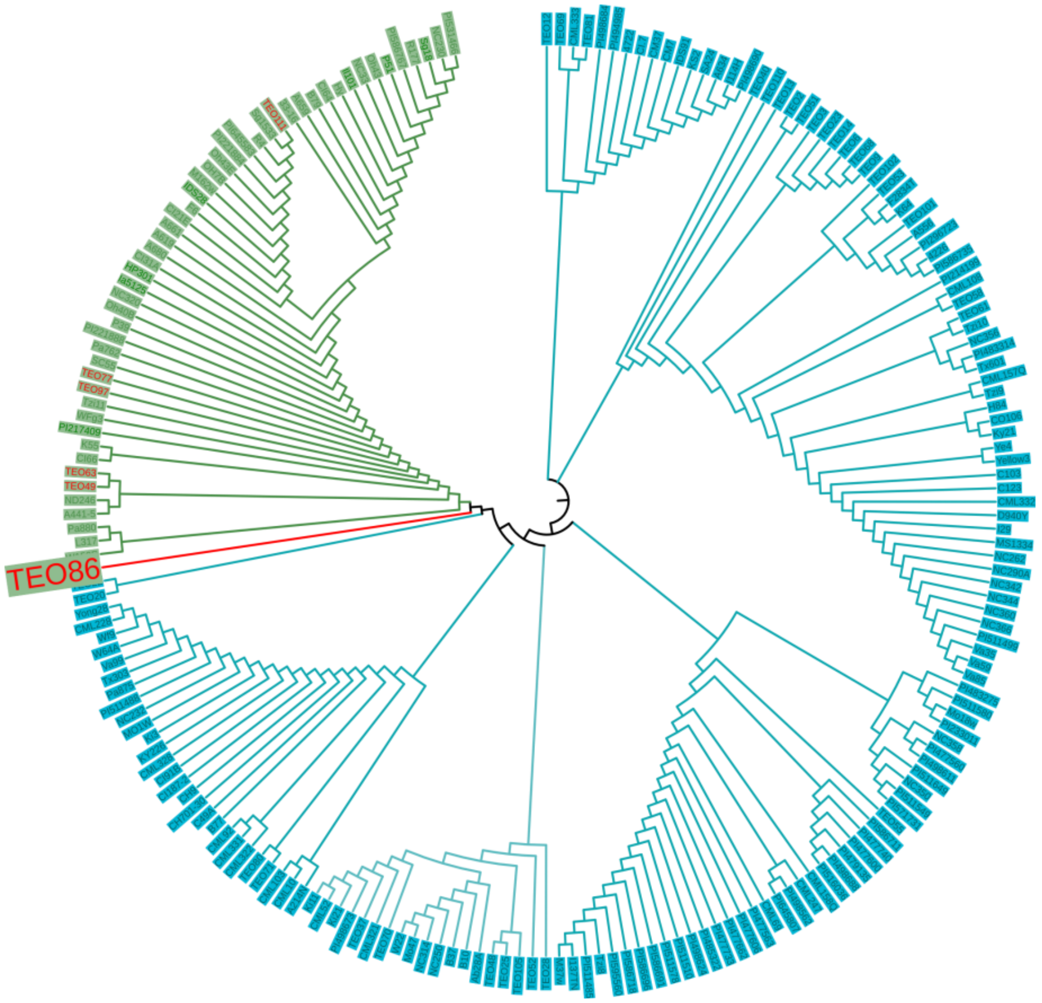

Supplementary Figure 18. Phylogenetic trees based on the *tin1* gene.

Phylogenetic tree analysis revealed that all the lines with the splice variant of "C/GT" were grouped into a single clade (green). The teosinte lines with the splice variant of *tin1* were highlighted in red.

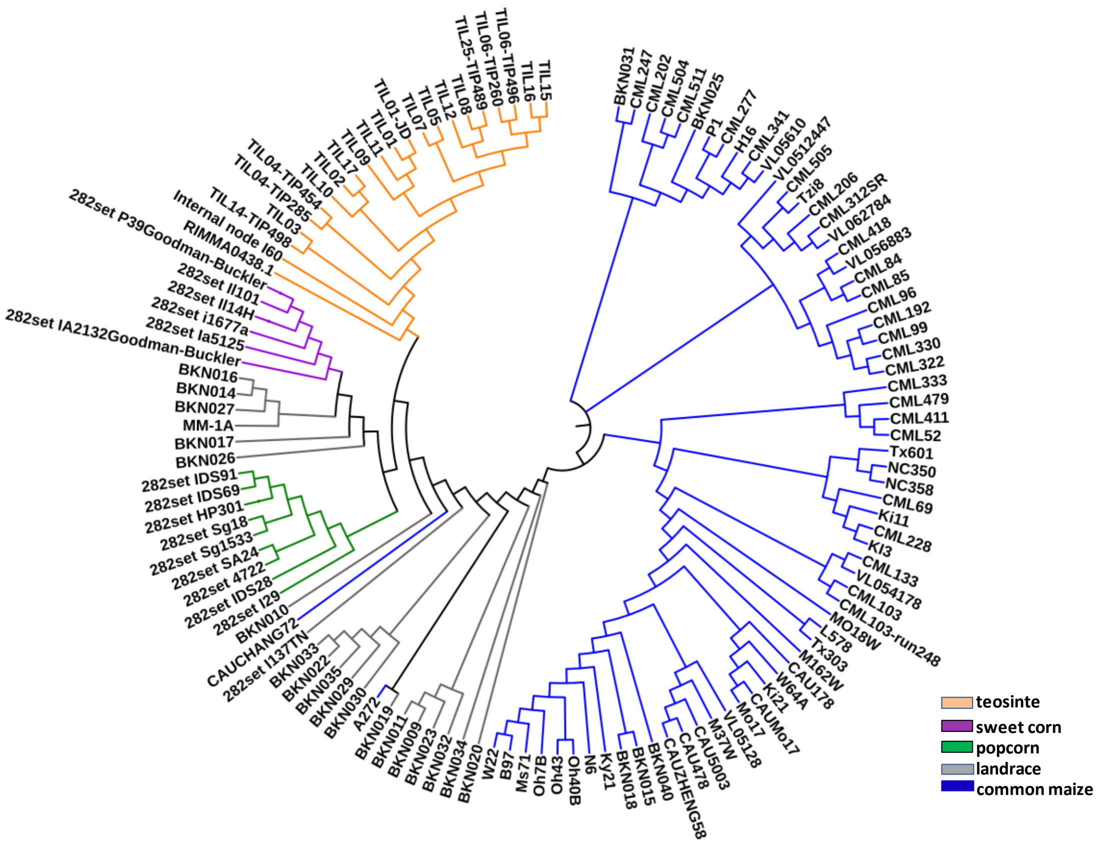

Supplementary Figure 19. Phylogenetic trees based on genome-wide SNPs.

Phylogenetic tree based on genome-wide SNPs (HapMap III).

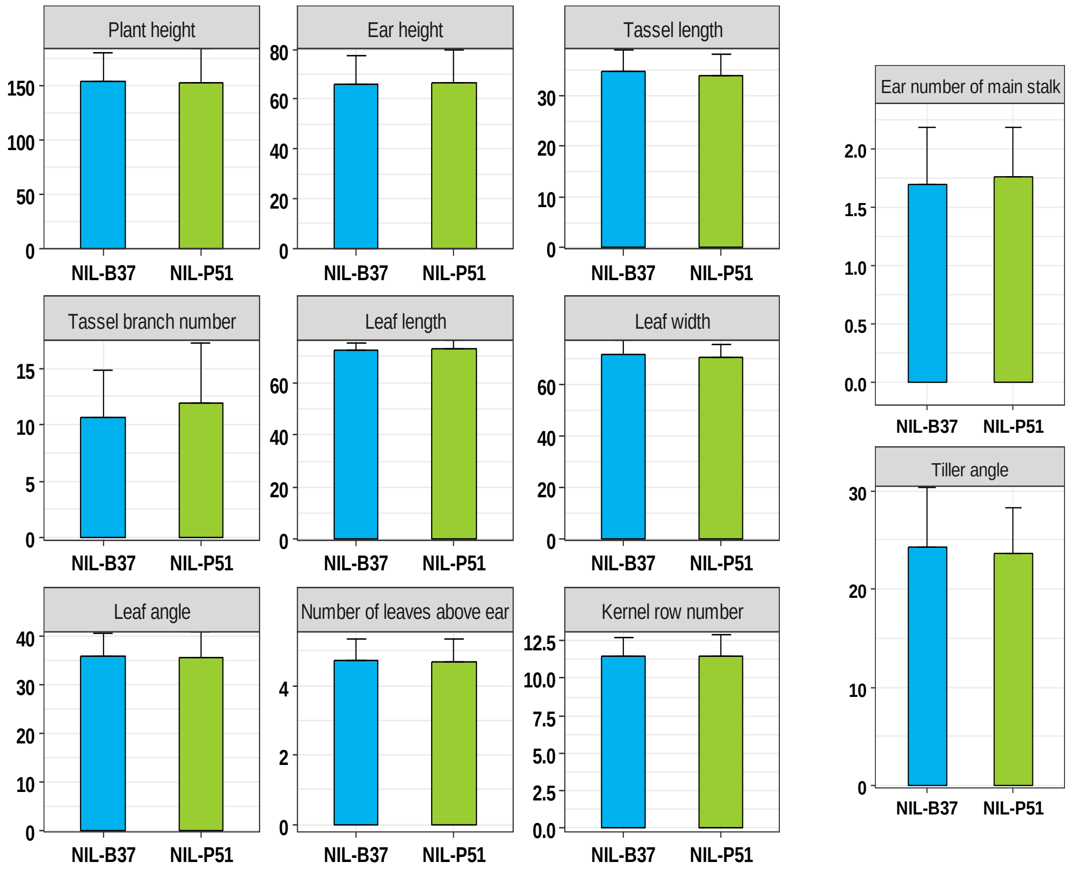

Supplementary Figure 20. Phenotypic comparisons between NIL-B37 and NIL-P51.

Phenotypic comparisons for 11 traits including tiller angle, plant height, ear height, tassel length, tassel branch number, leaf length, leaf width, leaf angle, leaf number above ear, kernel row number and ear number on main stalk between these two NILs. None of these traits showed significant differences between these two NILs based on two tailed Student's t-test. Error bar, SD (n=30). Source data are provided as a Source Data file.
