# Supplementary material for: The tin1 gene retains the function of promoting tillering in maize
Source: Nat Commun. 2019 Dec 6;10:5608. doi: 10.1038/s41467-019-13425-6 (PMC6898233; doi:10.1038/s41467-019-13425-6)
Supplement: Supplementary file 4 — Description of Additional Supplementary Files [file 41467_2019_13425_MOESM4_ESM.docx]

**Descriptions of Additional Supplementary Files**

File name: Supplementary Data 1

Description: Sequence alignment in the *tin1* gene between B37 and P51

File name: Supplementary Data 2

Description: Differentially expressed (DE) genes between the pair of NILs in early and late stages of tiller bud development based on RNA-seq (FPKM)

File name: Supplementary Data 3

Description: GO term enrichment analysis of the 436 specific DE genes from early stage

File name: Supplementary Data 4

Description: GO term enrichment analysis of The 685 specific DE genes from late stage

File name: Supplementary Data 5

Description: GO term enrichment analysis of the 259 overlapped differentially expressed (DE) genes shared between early and late stages

File name: Supplementary Data 6

Description: 75 tiller candidate genes from rice

File name: Supplementary Data 7

Description: *tin1* sequence comparison between wild and domesticated foxtail millets

File name: Supplementary Data 8

Description: Maize inbred lines for association mapping (282set)

File name: Supplementary Data 9

Description: Maize accessions for phylogenetic tree analysis and DNA diversity analysis

File name: Supplementary Data 10

Description: Foxtail millet population for association mapping and DNA Diversity Analysis

File name: Supplementary Data 11

Description: Primer list
