# Supplementary material for: The tin1 gene retains the function of promoting tillering in maize
Source: Nat Commun. 2019 Dec 6;10:5608. doi: 10.1038/s41467-019-13425-6 (PMC6898233; doi:10.1038/s41467-019-13425-6)
Supplement: Supplementary file 5 — Supplementary Data 1 [file 41467_2019_13425_MOESM5_ESM.pdf]

Supplementary Data 1

Sequence alignment in the *tin1* gene between B37 and P51

```
tin1-B37  1    TTAATAAACGGTGGTCCAGGGAAAATGAGGAACTTGTAAAAAAAGTTTTTATATTAGTGTTA 62
tin1-P51  1    ATAATAAACGGTGGTCCAGGGAAAATGAGGAACTTGTAAAAAACGTTTTTATATTAGTGTTA 62
                S3

tin1-B37  63   CAGTGACGTTGTTAGGGTTTATGGCAGTTAGCAGCATGGGATGAATGCAAATGACACATTGC 124
tin1-P51  63   CAGTGACGTTGTTAGGGTTTATGGCAGTTAGCAGCATGGGATGAATGCAAATGACACATTGC 124

tin1-B37  125  TATTACCTGCATTAG----GGCCATACAGAAGCGGATTCCTCCTTACAACCCTAGCTAGTGA 182
tin1-P51  125  TATTACCTGCATTAGCTAGGGCCATACAGAAGCGGATTCCTCCTTAGAACCCTAGCTAGTGA 186

tin1-B37  183  GCTCGCTTCTGTTCATCGAGAAGGGATCGCGATAGTGAGGAAGACCTAACGTTAATGAACAA 244
tin1-P51  187  GCTCGCTTCTGTTCATCGAGAAGGGATCGCGATAGTGAGGAAGACCTAACGTTAATGAACAA 248

tin1-B37  245  AGGACTACTAGCTCGCTTTCGACTCTGTAAAAAATCGCTAAATCCTTAGGGTGTATTTGGTT 306
tin1-P51  249  AGGACTACTAGCTCGCTTTCGACTCTGTAAAAAATCGCTAAATCCTTAGGGTGTATTTGGTT 310

tin1-B37  307  TGGATTTTTATTATAGCTTTTGCCCTCAATAAC-AAAAAAACAAATTAAGGGCTAGTTTGGG 367
tin1-P51  311  TGGATTTTCGTTATAGCTTTTGCCCTCAATAACCAAAAAAACAAATTAA------------- 359

tin1-B37  368  AACCATATTTTTCCAAGAGTTTTTCATTTTCCCAAGGGAAATTAGTTCATTTTTCCTTGGGA 429
tin1-P51  360  -------------------------------------------------------------- 359

tin1-B37  430  AATTGAAAATCCTATGAAAAAATGTGGTTGACAAACTAGCTCTAAAAGGCTAAATCCAGCCA 491
tin1-P51  360  ---------------------------------------------AAGGCTAAATCCAGCCA 376

tin1-B37  492  GCAATTTTCCAAAAACCGGCTTTTTCATAGCGCAAAATAAAAGCATATATAAACATGCTTTT 553
tin1-P51  377  GCAATTTTCCAAAAACCGGCTTTTTCATAGCGCAAAATAAAAACACATATAAACATGCTTTT 438

tin1-B37  554  AGCCGTTTCCAATTGAAGAACTTTGCAAATATACGGAACAACTTTTAGTGAATTT----TAG 611
tin1-P51  439  AGCTGTTTCCAATTGAAGAACTTTGCAAATATATGGAATAACTTTTAGTGAATTTAATTTAG 500

tin1-B37  612  CTTTTTTACAACTCATAGCATATAGTACCTTTTTTCAATGCTCACAGCTCATAATAGTTTTT 673
tin1-P51  501  CTTTTTTACAACTCATAGCCTATAGTACCTTTTTTCAATGCTCACAGCTCATAATAGTTTTT 562

tin1-B37  674  TTCACAACCACAATCTAACCAAACAATCCTATAATAAAGGATATAGATGGTCAAGTGAGTCA 735
tin1-P51  563  T-CACAACCACAACCTAACCAAACAATCCTATAATAAAGGATATAGATGGTCAAGTGAGTCA 623

tin1-B37  736  TGCTAAATGGTCGACACTAACCAATGGTGTTTCGGCACAGCACAACATGATCCATATATGGG 797
tin1-P51  624  TGCTAAATGGTCGACACTGACCAATGGTGTTTCG-CACAGCACAACATGATCCATATATGGG 684

tin1-B37  798  CCTGTTTGGTTCACTGCCTAATTTACCACATTTTGCCACACTTTTATGTCTAAGGCTAGTTC 859
tin1-P51  685  CCTGTTTGGTTCGCTACCTAATTTACCACATTTTGCCACACTTTTGTGCCTAAGGTTAGTTC 746

tin1-B37  860  TTCAATTCGAACGACTAACCTTAGGCAAAGTGTGGCATAGTTAGCCACAAACCAAACATGTA 921
tin1-P51  747  TTTAATTCGAACGACTAACCTTAGGCAAAGTGTGGCATAGTTAGCCACAAACCAAACATGTA 808

tin1-B37  922  TAGTGTTCGTGTCGGCACGACACGTGGCCCAATGTCGTGTCTAGACCGCCACCCTGGCACAA 983
tin1-P51  809  TAGTGTTCGTGTCGGCACGGCACGTGGCCCAATGTCGTGTCTAGACCGCCACCCTGGCACAA 870

tin1-B37  984  TGGGCAGGCACGTGCATGGCAATATTAGACTGTTGGGAATAGTCTCACATTGTGTGTAGTGG 1045
tin1-P51  871  TGGGCAGGCACGTGCATGGCAATATTAGACTGTTGGGAATAGTCCCACATTGTGTGTTGTGG 932

tin1-B37  1046 GTGGGCAACATGGTTTATATGGTTGAGAGTGTAACCCCTAATGAGCTAGCTTTTTGAGGGTG 1107
tin1-P51  933  GTGGGCAACATGGTTTATATGATTGAGATTGTAACCCCTAATGAGCTAGGTTTTTGAGGGTG 994

tin1-B37  1108 AGGTGTTGGCCCAACAGACCTAAAGCCTGCTGCTATGCGTTCGGAGGCGTGTGTCGTGGCCT 1169
tin1-P51  995  AGGTGTTGGCCCAACAGACCTAAAGC-TGCTGCTATGCGTTCGGAGGCGTGTGTCGCGGCCT 1055

tin1-B37  1170 CACGGCCTAGATGGGCATGTCGTATGAGCAGACATAGAGATTGTTGGGAATAGTCCCACATT 1231
tin1-P51  1056 CACGACCTAGATGGGCGTGTCATATGAGCAGACAGAGAGATTGTTGGGAATAGTCCCACATT 1117

tin1-B37  1232 GTGTGTTCTAGTTGGACAAGCATGATTTATATGGTTGAAGATGTAACTCATAATGTGCTAGC 1293
tin1-P51  1118 GTGTGTTCTAGTTGGACAAGCATGATTTATATGGTTGAGGATGTAACCCATAATGTGCTAGC 1179

tin1-B37  1294 TTTTTAGGGAGTGTTGGCCCAATAAATCTAAAGACTGTGTTGCTATGCGTTCGGGCACAGGC 1355
tin1-P51  1180 TTTTTAGGGAGTGTTGGCCCAATAAATCTAAAGACTGT--TGCTATGCGTTCGGGCACAGGC 1239

tin1-B37  1356 ACACATGTGTCGGTGGTCCGGTTCCAATATAGACACGACTTAAACCAACTGGTGATCTATAG 1417
tin1-P51  1240 ACACATGTGTCGGTGGTCCGGTTCCAATATAGACACGACTTAAACCAACTAGTGATTTATAG 1301
```

```
tin1-B37  1418 ATGTGATTGTTATGTGCACATGTTATTGTGAATTATGATTTGTTATTGTTTTAAATATAATA 1479
tin1-P51  1302 ATGTGATTGTTATGTGCACATGTTATTGTGAATTATGATTTGTTATTGTTTTAAATATAATA 1363

tin1-B37  1480 AATCTACTCTATATTTCTATGGATGTTCATGTTTTAAAAATATATATGTAATCTTAAAGATG 1541
tin1-P51  1364 AATCTACTCTATATTGCTATGGATGTTCATGTTTTAAAAATATATATGTAATCTTAAAGATG 1425

tin1-B37  1542 ATATGGATTTTTAAGCTCTTAAAATAGTGCCTAGATTGTCACCGGTATGTACAACACAATTA 1603
tin1-P51  1426 ATATGGATTTTTAAGCTCTTAAAATAGTTCCTAGATTGTCACCGGTATGTACAACGCAATTA 1487

tin1-B37  1604 GGTGTTGTAGTGTCCTGTCTCGACTGATGGCTAGTCAGTAGTGCTCGCATGACACGGTACGA 1665
tin1-P51  1488 GGTGTTGTAGTGTCCTGTCTCGACTAATGGCTAGTCAGTAGTGCTCGCATGACACGGTACGA 1549

tin1-B37  1666 TTACTAACAGTTTCCAATAATATTGTGCGTAAAGTGCCATGCTTGTTTGGCAATCTCTAATA 1727
tin1-P51  1550 TTACTAACAGTTTCCAGTAATATTGTGCGTAAAGTGCCATGCTTGTTTGGCCATCTCTAATA 1611

tin1-B37  1728 AATCACTACTGAACTCGACTTATTTGCCTAGTGTCATAGACACTTGGCAAAGATCTTTTT-C 1788
tin1-P51  1612 AATCACTACCGAAGTCGTCTTATTTGCCTAGTGTCCTAGACACTCGGCAAAGATCTTTTTGC 1673

tin1-B37  1789 ACTCGACAAAGCCTTTATTGAGTATTACACTCGACAACGAATACTCGGTAAAAAATTTATCA 1850
tin1-P51  1674 ACTCGGCAAAGCCTTTATTGAGTATTACACTCAACAACGAATACTCGGTATAAAATTTATCA 1735

tin1-B37  1851 GCAAACCCTTTGCCGAGTGTAAAAAACACTAGACAAATAAAAGCACCCGGCAATAAAAATCG 1912
tin1-P51  1736 GCAAACCCTTTGCCGAGTGTAAAAAACACTAGGCAAATAAAAGCACCCGGCAATAAAAATCG 1797

tin1-B37  1913 CTAAAAATCCGGAAAACATAGGAAAACATTAATTAGGGGACAACCCCCAATCAACGCCATTG 1974
tin1-P51  1798 CTAAAAATCCGGAAAACATAGGAAAACATTAATTAGGGGACAACCCCCAACCAACGCCATTG 1859

tin1-B37  1975 TCCTACCCATTTCTCTACCATTTTTTGATCTGAATTCACGTGTTTCGCAGCCGGTGGGATTC 2036
tin1-P51  1860 TCCTACCCATTTCTCTACCATTTTTTGAGCTGAATTCACGTGTTTCGCAGCCGGTGGGATTC 1921

tin1-B37  2037 AATCTCGCAAATCTCTCTCGAGCATAACCTACTCTACCACTCCACTTCTACCTCACTTATGT 2098
tin1-P51  1922 AAACTCGCGAATCTCTCTCGAGCATAACCTACTCTACCACTCCACTTCTACCTCACTTATGT 1983

tin1-B37  2099 TTATATTACGATTTTCCTTCCCTAAGGGGGTGTTTGGTTACACCCCGCTAAAATTTAGCCCA 2160
tin1-P51  1984 TTATATTACGATTTTCCTTCCCTAAG------------------------------------ 2009

tin1-B37  2161 TGTCCCATCGAATGTTTGAACCTCTGTTCCGGGTATTAAATGTAGTCGGATTATAAAACTAA 2222
tin1-P51  2010 -------------------------------------------------------------- 2009

tin1-B37  2223 TTTGTCAGCCGAAGATTAAAAGACGAGACGAATCTAGTCCAGTTGGTTGGGTCTATATTTCA 2284
tin1-P51  2010 -------------------------------------------------------------- 2009

tin1-B37  2285 TACTTCTATTTAAAAGTCAAACGCTTGATGTGACCCGGGCTAAACTTTAGCAGGAGCAACCA 2346
tin1-P51  2010 -------------------------------------------------------------- 2009

tin1-B37  2347 AACACCCCCTAAGTATTATAATAATCCAAGAGTAAATTGATTGTTTAAGACACTAAATAAAT 2408
tin1-P51  2010 -------------TATTATAATAATCCAAGAGTAAATTAATTGTTTAAGACACTAAATAAAT 2058

tin1-B37  2409 TCAAATGAAAAGTTGTTAACTACAGAGTTGCATAACTCTGTGAGAGTTACAACTTTCATTTT 2470
tin1-P51  2059 TCAAATGAAAAGTTGTTAACTACAAAGTTGCATAACTCT-TGAGAGTTACAACTTTTATTTT 2119

tin1-B37  2471 AGTAATTTCTTCATCCAAGATCGTTTACAAAATTTGAATTTTCAATTTGAAAACTTCAAACT 2532
tin1-P51  2120 AGTAATTTCTTCA-CCAAGATCGTTTACAAAATTTGAATTTTCAATTTGAAAACTTCAAACT 2180

tin1-B37  2533 TACAAATCAAAAGGTTGTCAACTATACAGTTAAATAACTTTTTGAGACCTACAACTTTCATT 2594
tin1-P51  2181 TACAAATCAAAAGGTTGTCAACTATACAGTTAAATAACTTTTTGAGACCTACAACTTTCATT 2242

tin1-B37  2595 TTTGTCGTTTTTTTCATCCGAGGTCGTTTGTAAAATTCAAATTTTAAATTCAAATATAGTTT 2656
tin1-P51  2243 TTTGTCGTTTTTTTCATCCGAGGTCGTTTGTAAAATTCGAATTTTAAATTCAAATATAGTTT 2304

tin1-B37  2657 TGCATGACTAGATGATTTTAAATCAAAAAGT-TATCAACTACAAAGTTTAATAACATTTTAA 2717
tin1-P51  2305 TGCATGACTAGATGATTTCAAATCAAAAAAACTATCAATTACAAAGTTTCATAACATTTTAA 2366

tin1-B37  2718 GACCTATAACTATCATTTT-CATGGGTTTTCCATCCGAGATCGTTTCACAAATTCAAATTTG 2778
tin1-P51  2367 GACCTATAACTATCATTTTTCGTGGGTTTTCCATCCGAGATCGTTTCACAAATTCAAATTTG 2428

tin1-B37  2779 TCTAGTGTAAAAAA-TGCACTCGACAAAAAA-CTCTTTGTCTAATGTCGAAAAAAAACACTC 2838
tin1-P51  2429 TCTAGTGTAAAAAAATGCACTCGACAAAAAAACTCTTTGTCTAGTGTCGAAAAAAA-CACTC 2489
```

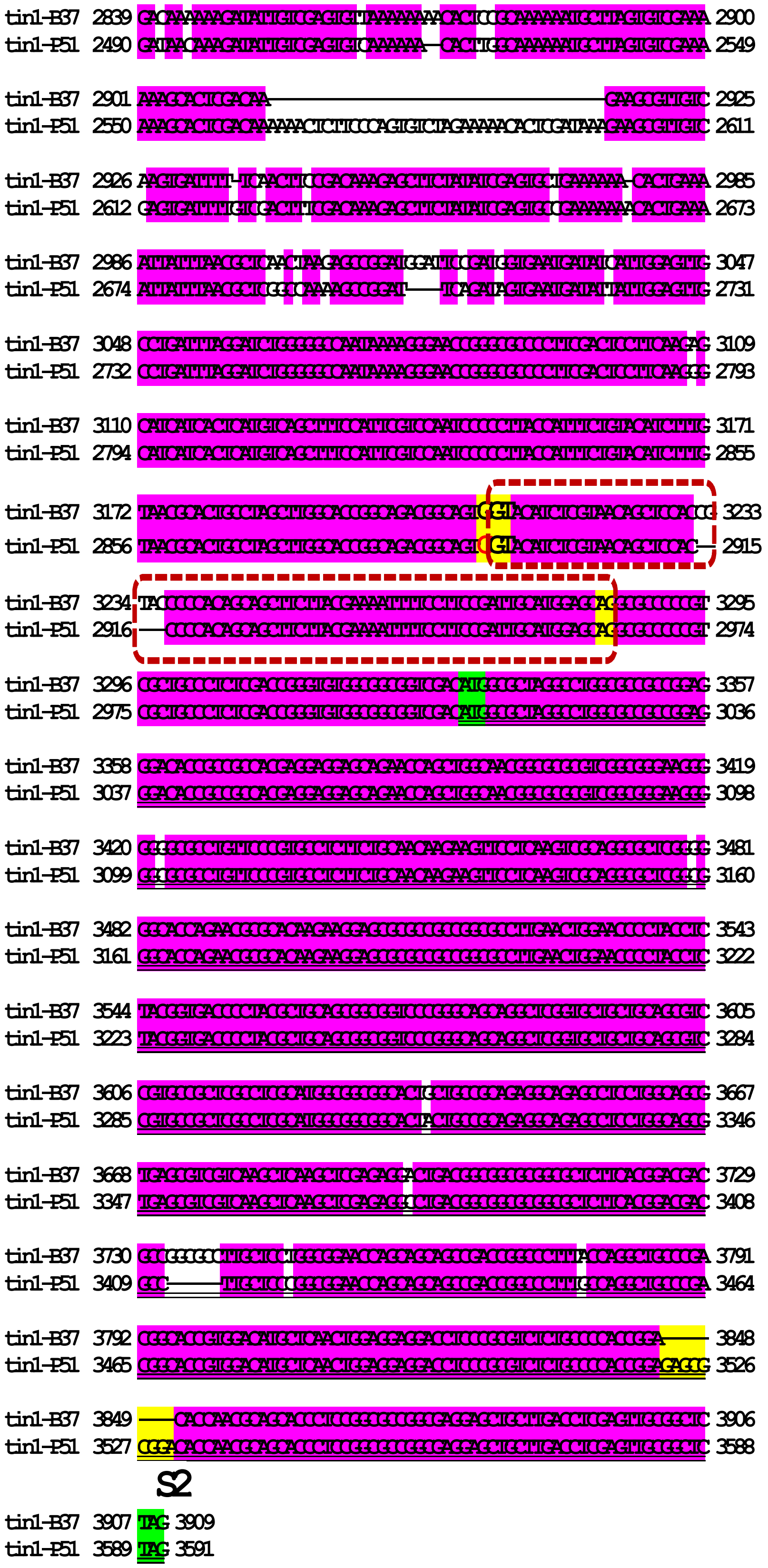
tin1-B37 2839 2900
tin1-P51 2490 2549
tin1-B37 2901 2925
tin1-P51 2550 2611
tin1-B37 2926 2985
tin1-P51 2612 2673
tin1-B37 2986 3047
tin1-P51 2674 2731
tin1-B37 3048 3109
tin1-P51 2732 2793
tin1-B37 3110 3171
tin1-P51 2794 2855
tin1-B37 3172 3233
tin1-P51 2856 2915
tin1-B37 3234 3295
tin1-P51 2916 2974
tin1-B37 3296 3357
tin1-P51 2975 3036
tin1-B37 3358 3419
tin1-P51 3037 3098
tin1-B37 3420 3481
tin1-P51 3099 3160
tin1-B37 3482 3543
tin1-P51 3161 3222
tin1-B37 3544 3605
tin1-P51 3223 3284
tin1-B37 3606 3667
tin1-P51 3285 3346
tin1-B37 3668 3729
tin1-P51 3347 3408
tin1-B37 3730 3791
tin1-P51 3409 3464
tin1-B37 3792 3848
tin1-P51 3465 3526
tin1-B37 3849 3906
tin1-P51 3527 3588
S2
tin1-B37 3907 TAG 3909
tin1-P51 3589 TAG 3591
