# Supplementary material for: The tin1 gene retains the function of promoting tillering in maize
Source: Nat Commun. 2019 Dec 6;10:5608. doi: 10.1038/s41467-019-13425-6 (PMC6898233; doi:10.1038/s41467-019-13425-6)
Supplement: Supplementary file 11 — Supplementary Data 7 [file 41467_2019_13425_MOESM11_ESM.docx]

**Supplementary Data 7 *tin1* sequence comparison between wild and domesticated foxtail millets**

**The three copies of *tin1* were marked in green. One entire and a half copies were deleted in the 18-kb deletion in domesticated foxtail millet. Si, domesticated foxtail millet; Sv, wild foxtail millet.**

************************************************* ******************************


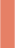

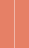

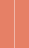

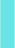

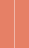

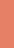

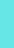

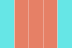

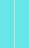

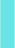

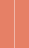

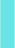

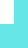

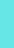

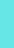

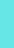

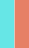

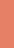
Si-tin1 TAGAAGAAGTTCGAAGTTTAGTCTTTCAAACTTCCTTTCTTAATTTCTTTCTTTCTTGTCTTTTGTGTTTCTCATTTTGA 80

Sv-tin1 TAGAAGAAGTTCGAAGTTTAGTCTTTCAAACTTCCTTTCTTAATTTCTTCCTTTCTTGTCTTTTGTGTTTCTCATTTTGA 80

******************** ***************** **** ************************* **********


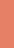

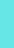

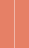

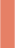

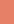

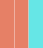

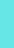

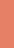

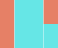

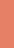

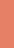

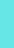

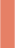

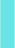

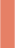

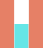

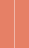

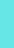
Si-tin1 GGTTTGGAGTCTAAGTATTTATGTAACTTCTGGTTATGTGACCAGATTTATTTTCTTTATGGTCGTAGATATTAATGTTC 160


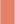
Sv-tin1 GGTTTGGAGTCTAAGTATTTGTGTAACTTCTGGTTATGAGACCCGATTTATTTTCTTTATGGTCGTAGACATTAATGTTC 160

************************* ********************************** *******************


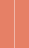

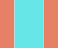

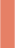

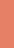

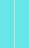

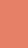

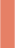

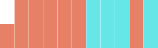

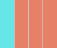

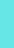

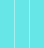

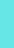

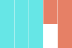

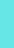

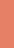

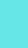

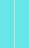

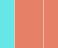
Si-tin1 AAGACCAGATATTTTCCTTAATTAGGAAAAACCCACTCAAATCTTGCCCTGGGTTCGCCCAAGGTCGATCCGCCGCAAAT 240

Sv-tin1 AAGACCAGATATTTTCCTTAATTAGAAAAAACCCACTCAAATCTTGCCCTGGGTTCGCCCTAGGTCGATCCGCCGCAAAT 240

************************************************** *****************************


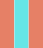

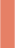

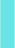

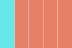

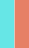

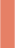

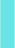

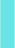

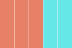

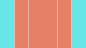

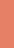

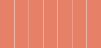

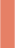

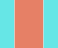
Si-tin1 GTTACAGGAGTCTTCAAAAGTTCATGAGCTTTTCGGAAACCTCAAAACGA-TTTTTTTAAAAAAATATTGCAACGGTTGT 319

Sv-tin1 GTTACAGGAGTCTTCAAAAGTTCATGAGCTTTTCGGAAACCTCAAAACGATTTTTTTTAAAAAAATATTGCAACGGTTGT 320

********************************************************************************


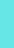

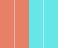

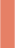

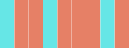

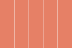

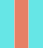

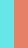

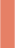

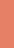

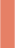

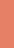

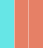

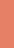

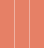

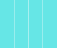

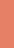

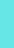

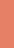
Si-tin1 CTGAACCTAGTTCAACAAACATTTAAAAATCACGCAGGATTTATTTAGGGAGCAAGTGGAGAAAGTTCCCCTGTTAGCTA 399

Sv-tin1 CTGAACCTAGTTCAACAAACATTTAAAAATCACGCAGGATTTATTTAGGGAGCAAGTGGAGAAAGTTCCCCTGTTAGCTA 400

********************************************************************************


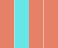

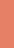

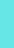

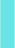

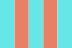

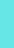

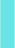

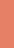

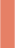

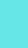

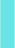

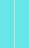

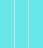
Si-tin1 ACAATGTAGCTGTCTGTCACACTGTCTTCTTGTGAGTTGTGATGCCTTCGTCCTTCCCTTCTATAAATAGTTATATAAGT 479

Sv-tin1 ACAATGTAGCTGTCTGTCACACTGTCTTCTTGTGAGTTGTGATGCCTTCGTCCTTCCCTTCTATAAATAGTTATATAAGT 480

***************************** ******** ******** ******************************

Si-tin1 GCCTCTTCCATCCACTCACAGTCACAGCTCTCCTCCTCCACTTGTGTTGATACCAGCTAGATCCGATGTTGCAATAAAAT 559

Sv-tin1 GCCTCTTCCATCCACTCACAGTCACAGCT---CTCCTCCATTTGTGTTGGTACCAGCTAGATCCGATGTTGCAATAAAAT 557

********************************************************************************

Si-tin1 ATAGCAAATCCAGAACTTGCATTTGGGGAATGCTAGTAGCTCCTTGTTAGACCTTTTATGGAGTAGCAGCTGTGGATCGA 639

Sv-tin1 ATAGCAAATCCAGAACTTGCATTTGGGGAATGCTAGTAGCTCCTTGTTAGACCTTTTATGGAGTAGCAGCTGTGGATCGA 637

********************************************************************************

Si-tin1 ATCCTGTAGAGATATATATCTCCATGGAGTCCTCCAAACGTGCTCCACCCTGTTCCGACGACGCAAGCGGTGGCGGCGGC 719

Sv-tin1 ATCCTGTAGAGATATATATCTCCATGGAGTCCTCCAAACGTGCTCCACCCTGTTCCGACGACGCAAGCGGTGGCGGCGGC 717

********************************************************************************

Si-tin1 GGCGGCGGCGCGCGGCTGTTCCCGTGCCTGTTCTGCAGCAAGACGTTCCTCAAGTCGCAGGCGCTGGGCGGGCACCAGAA 799

Sv-tin1 GGCGGCGGCGCGCGGCTGTTCCCGTGCCTGTTCTGCAGCAAGACGTTCCTCAAGTCGCAGGCGCTGGGCGGGCACCAGAA 797

************************* ******************************************************

Si-tin1 CGCGCACAAGAAGGAGCGCGTCGCCGGCGGCGGCGACTGGAACCCCTACGCCACCTCCTACGCCGCGGCGCTCGAGCTCG 879

Sv-tin1 CGCGCACAAGAAGGAGCGCGTCGCCAGCGGCGGCGACTGGAACCCCTACGCCACCTCCTACGCCGCGGCGCTCGAGCTCG 877

**************************************************** ***************************

Si-tin1 ACGCGCTCGTGGCGGCCGCCGCCGGCAGCGCGATCCCCACCACGTACGCGCTCGTGGCGGGCGCCCAGCACTGCGGCGGC 959

Sv-tin1 ACGCGCTCGTGGCGGCCGCCGCCGGCAGCGCGATCCCCACCACGTACGCGCTGGTGGCGGGCGCCCAGCACTGCGGCGGC 957

********************************************************************************

Si-tin1 GCTGGTGGCGGCGCCAGGGCTGGGGAGGCGTACAGGGACTCCGGCGCCGCCGCCATCGCGGCGCGGCGCTTGGAGCTGGT 1039

Sv-tin1 GCTGGTGGCGGCGCCAGGGCTGGGGAGGCGTACAGGGACTCCGGCGCCGCCGCCATCGCGGCGCGGCGCTTGGAGCTGGT 1037

******************************************************** ***********************

Si-tin1 GGACCGGTGGACGGGGGGCCCCTTGCCGGCCCTGAACGGCGGTACCGAGCACGACGCCGGCGACATGGACGGGTTGATGG 1119

Sv-tin1 GGACCGGTGGACGGGGGGCCCCTTGCCGGCCCTGAACGGCGGTACCGAGCACGACGGCGGCGACATGGACGGGTTGATGG 1117

*****************************************************

Si-tin1 ATGACGTGCTCAACTGGAGAAGGGGCACGCAGCAAGCCGTCCCCGTGGTGGTGG 1173

Sv-tin1 ATGACGTGCTCAACTGGAGAAGGGGCACGCAGCAAGCCGTCCCCGTGGTGGTGAAGGAGACGGCGGCGTTGGCGAGCTCC 1197

***** **

Si-tin1 ---------------------------------------------------------TGGTGGTG 1181

Sv-tin1 GGCGGCGGCGAGGAGCCCGATCTGGAGCTGAGGCTCTGGCCAGCTTCGTGACGACCTTGGTGTTGTTCTTCGAGCGCCCC 1277 Si-tin1 1181

Sv-tin1 TGCCGCTCTGTTGTCGTCTTGTGTGGTTGCCCACCGTTGTCACTACTGTTGCTTGCTCGTGTGCTGGTTGTTGGATGACA 1357

Sv-tin1 AGCCTGCATGTACCATCCTCGATGCTCTATACTCCCTCCGTTCCAAATTATAAGTCATTCCAACTTTCTTGGAGAGTCAA 1437

Si-tin1 1181

Sv-tin1 AGCATCTCAAGTTTGACCAAATTTATACAATAAAATGCTAACATTCGTGATATCAAATAGGTATCATTAGTTTCTTCATT 1517

Si-tin1 1181

Sv-tin1 AAATATATTTTCATAGTATATCTATTCGGTGCCATAAACTTTTGTGATTTTCTCTATAATTTTAGTCAAACATAAAAATG 1597

Si-tin1 1181

Sv-tin1 TTTGACTCTCCAAGAAAGTTGGAATGACTTATAATTTGGAACGGAGGGAGTATTATATATATTTTTAATATCAACATGCT 1677

Si-tin1 1181

Sv-tin1 GATGATTCTTGGCTGCCGCATGCCCCATCTCCATTTTTTTTTATCCTTGCTTGTTGTTCTCCTCCATCATTTCTTCTAAA 1757

Si-tin1 1181

Sv-tin1 CAAAATTCGGCAAATTAAAGCTCTTCTATCATCGGTTTAAAAGAAGAACACGGTACACATTCATGCATGCGTGAACGCAC 1837

****************** ****

Si-tin1 GTGTGCATGCAATAGTAGGACATC 1205

Sv-tin1 GTGTGCATGCAATAGTAGTGCATCATTTCTCTTAGCTTGGGATGTATTCAACATTTGCCTTGGACAAAGAAAAATGAATT 1917

Si-tin1 1205

Sv-tin1 CTAATACTAATGGCATATGCTGCTGCACTGCAAGTAGAGGAGAGATGGAAGAGTTGCAGTTAGCTTAGGCAGCACAGCGG 1997

Si-tin1 1205

Sv-tin1 TATCACCTGTTGCTTCAAGTGGATGCCAGTAGCTAGGAGCTGAAATCATCATGGCTCCACTCCACACTCCCACTGATAGC 2077

Si-tin1 1205

Sv-tin1 TGCTGCTGACAATGCCGCGCTGACTCTTCCAAGCTTACTAGTAGCTTTATACTTACACAAGACCTGGTATGACTTTTCCA 2157

Si-tin1 1205

Sv-tin1 AGGTACCCAACCAGGCCCAGGCTGGGGAACCGGACAAGAAGCGCGTCCACTTCCGGGGAGGATGGTGTCACGTGGGCACG 2237

Si-tin1 1205

Sv-tin1 CACAAGCACGACGCAACGCAGATGGGTGCGTGCGAGCGTGACAGCGGCTGCAGCTAGCCAAGGGGACCAGATCGACGCAG 2317

Si-tin1 1205

Sv-tin1 CGATCCACAGTGTGCCTGCCTTTGGATTCCTGAATCCTGATGACGATGATGCACTACGGGAAACCGTAAATTTACCGAGT 2397

Si-tin1 1205

Sv-tin1 ATTTTTTCTTTGCTGAGTGTTTTTTTTCGAACACTCGGCAAACAAGCTCTTTACCGAGTGCCGAGCTAAAAATACTCGGC 2477

Si-tin1 1205

Sv-tin1 AAAGAAAAAACACTCGGCAAAGAGGAGCTTTGCCGAGTGTCAAAAATAAAATACTCGGCAAAGCCTATTTACCGAGTGTT 2557

Si-tin1 1205

Sv-tin1 TTATTTTTGACACTCGGCAAATTTTTTTTTGAAAAAAAATCTTTGCCGAGTGTCCAGATCGGGACACTCGGCAAATGTCA 2637

Si-tin1 1205

Sv-tin1 AAAATATATCTCTTCCCCAGCCCCCTACCCAGCACCCCACCCGCCCGGTGCGCCCCTCCTTAGCTTCCTCCGCGCAACAC 2717

Sv-tin1 CGCCCGACGCCCCCCTCCTCTTCCTCCGCGCAACACCGGCCCCCCACCTCCCTCTCCCGCCGCCGGCTAGCCGCCGCTGC 2797

Si-tin1 1205

Sv-tin1 CGCCGCCCCTTCCCTCCCTCCCTCCCTCTCCCTCTCCTACCGGCACCCCTCCCCCTCCTCTCCCCTCCTCCCTCCCTCGC 2877

Si-tin1 1205

Sv-tin1 GCGGCCGGACCCTCTCTCCGGCGGCGTCTCCGGCTGCCGCCCCGCCCCTCTCCCCAGATCCGACCGGATCCGGCCGCCGC 2957

Si-tin1 1205

Sv-tin1 ATCGCCCCTCTTCCCCGGATCCGGCGGGGACTCGTGTTGGGGGCCCTGGGAGCGGCGGCGTGGAGATGTGGTGGTGGTGG 3037

Si-tin1 1205

Sv-tin1 TGGCCGGGAGGAGGCAGCTCGTGGTGGCCGGTGGGAGGCAGCTCGTGGTGGTCGGCGGGAGGCAGCTCGTGGTGGTCGGC 3117

Si-tin1 1205

Sv-tin1 GGGCTGTGGTGGTGGTGGTGGTCGGCTGTGGTGGTGGCAGCGGCGGTGGCGGTGTGGATGGTGGCGGCGGGCGTGGATGG 3197

Si-tin1 1205

Sv-tin1 TGGCCGGCTGTGGTTGTGGCGGCGGCGGCGGCGACGTGGATGGTGGCGACGTGCTGGTGGTGCGGCCGACGGTGTCGGCG 3277

Si-tin1 1205

Sv-tin1 TGCCTTTTTTTATTTTTTTCGAAAAAATGGTTTGCCGAGTGTTTTCTAGGCACTCGGCGAAAGCTTTGCCGAGTGCCCGA 3357

Si-tin1 1205

Sv-tin1 CCGAAAACACTCGGCGAATGCTACTTTGCCAACTCAAATTTTGCCGAGTGCCAGATGCCGAGTGTAACACTCGGCGAATT 3437

Si-tin1 1205

Sv-tin1 TGTTGCCGAGAGTATTTTGGATTTCGCCGAGTGCCCCTGGCACTCGGCATAAAACCTGTGTCCGGTAGTGATGTCTCAAG 3517

Si-tin1 1205

Sv-tin1 AACCACACGTACGTCTAGACAAACGGCCTTCTATTTGTTCCATGGTCTAGACTACCATGCCTACAATGTGCCAAGGCTAA 3597

Si-tin1 1205

Sv-tin1 TCCCAACCCATTACTCTATATGCTATTCCGTCCGTAAATAAATGATAGACGCATTGTCATCAGAAAAAAAAGTCAAGCTT 3677

Si-tin1 1205

Sv-tin1 TATAAATTTTAACCAATAATAATTAGTTAAATTATAAGCACATTTAGTGTATAACTTTTCTCCGGTTCAAATATGCTTAT 3757

Si-tin1 1205

Sv-tin1 TATTCCTAGGAAGTATTATGCTCTCTAAAAATACCATGCCATTTTTTCAGTATAACGTGTCAATTTATCACTGTCTAGAC 3837

Si-tin1 1205

Sv-tin1 CACTGACTGTACGTGACAATGGCCCCAGACATACCTGCGGACCAGTACGTGGTTTTCGAAAGTTACCTTTCATGCTCAAG 3917

Si-tin1 1205

Sv-tin1 CTGTGCCTTCAGCAAAAAATGTCCTAATGTAAGTGACAATATGGCACCTAAGTAATGTCTATGATTTAATCATTAAGATT 3997

Si-tin1 1205

Sv-tin1 AGTCTAAGCAGGCAGCACTAGTGTACCCTACCTAGCTATCGGTGTGGTCCACTAGCGTGGAGTGTGTCTAGCACTACCCA 4077

Sv-tin1 CCGCAGAGGTTGGCACCTCGTACGGGCACAACGGAGATCGGAGAAGAGCTGCTTCCCCGTTGTACCAAATGCAAATTTTA 4157

Si-tin1 1205

Sv-tin1 ATGTACAGTTTCATTGTACTATTACTAACATTGAAAATTTCGTAACTGTGCATGCTAGGTGTCATAGGGATGAGACTCCT 4237

Si-tin1 1205

Sv-tin1 CTCATAATGACCTTGCCAAGTCAGCAAGTTTGCTGATGTGGCAACAAATTTAATGTTCATAAAACTCCTATAAAACCTCC 4317

Si-tin1 1205

Sv-tin1 ATTGAGACTGGCCTAAACGCGAGGCGGCAGATGCCCTAAGGAGTAACACAACGGGTCAACGGGCTGTCTCTCTGAGCGAG 4397

Si-tin1 1205

Sv-tin1 GGAGAGAGATCCTCCTGTAGTCCTGTGTCAGTTGACGCTGTGCTACTGTTGCTGTGGGCCAGCCCCACCAACATCATGTC 4477

Si-tin1 1205

Sv-tin1 CCATTCCTATTTTTTTGGTAGTAGGCCGGATTTTCCCGTTCCATAGTATCAATGATGGTATGCTGAGATCATTGGATAAA 4557

Si-tin1 1205

Sv-tin1 TATTGAGCTGCTTCGTACCATTGTTGTTCCTTGCCTCACCTTGCTCAGCAAGGATTTAAAGGTTTGGTTGATGTGCTGGC 4637

Si-tin1 1205

Sv-tin1 AAGGAATTTCCTTTCTATCATGAGAGAGCCAATCCAGCTCTCCTAAGCCACCAAGGCAAGGCTTGCTCTCGCCGACAAGG 4717

Si-tin1 1205

Sv-tin1 CGAGGCAAAGCTTGCCTGAGAACCAAGCTGACCCATAGGTGCAATATATATACACATGGATGTGAGTTCCGGTATTGCTG 4797

Si-tin1 1205

Sv-tin1 ATCCTGTATCATAGCTCCTAACTATACTTATCTTTTTCCCCTAAATTTGGAAGGGAAAAATCCTATTAATATTTCACCCT 4877

Si-tin1 1205

Sv-tin1 TCAACACTATTGTAAAAATCTAATTTTCAATCTACAACTATAAAACCGGATAGCAAGGGTCATCCAACTATCAAAACTAA 4957

Si-tin1 1205

Sv-tin1 GCAAATTTGACCCTTTGGGTGGTTTCAAGGGGGGTTTTGCATTTCCTAGAAATTTAAAAGTTTTGATTTAATCTAATAAT 5037

Si-tin1 1205

Sv-tin1 TCATAAGTAATTCATTTCGAAAAAATAAAACAGTACCAAAATATTTGCTGAAAATGTAATCTATCTGTTGGTGCTCTATT 5117

Si-tin1 1205

Sv-tin1 TGGTATTATTTGATTATTTGTTCTTATTGCTTGTATTTTATTAAAAGCAATAAAACACTAGCTAATAATAGTAACAAGTA 5197

Si-tin1 1205

Sv-tin1 AGATGTAAATAACTTCAAATCGAGCATCAACAAATAGCCTACATTTCTAGGAAAAATTACTCAATAGCCTACATTTCCTT 5277

Si-tin1 1205

Sv-tin1 GTATTTCTAAAAAGTTTACAATAGTTTACAAATATTACTTGACATGAGCATAGATATGGGAAAAAGGAAAAGAAGAAGAA 5357

Si-tin1 1205

Sv-tin1 ATTACTCAGCACTTTGTCAAATTTACATATATCTCGCAACAAAGTGAGTTTTCAAAACTGTAAACAAGTAACTTTTGCTT 5437

Sv-tin1 TGGCTGAAAAGTCACGGGACTACCTATACATGCATGTACTTGTACTGTGAATGGTGGGGTGCTTAATTAATTCTCCGCGT 5517

Si-tin1 1205

Sv-tin1 ATATTAGAGGAGATGAACAACAGGAATATAATTCGGGGTGTACTACATCGTTGCGAGTCCATATCAAAACTGTGAGTAAT 5597

Si-tin1 1205

Sv-tin1 CCTTATTAGCGTGAGCATAAAAGAGAGGCTAGAGACTGGCAATTTCTTGTGGAAATGGACTACGGTGCATCGAAACAACG 5677

Si-tin1 1205

Sv-tin1 GACTCATGCCTGTTTCAGTGAGACATTTGGACTGAATTTTCTCCGGTTTACTCAGGGTCTAGAGGAAATCAGAAAAAGGA 5757

Si-tin1 1205

Sv-tin1 ATTGCAGATGCGTGGCTCAACCAAGCTATGTGTGCAGTAGACATGCACCCATCATGCATAGACTCAAGTTACGCTCGAAA 5837

Si-tin1 1205

Sv-tin1 TAATTGAGAAAAATGAAAGATAATAAGCAATCACTGTTTGCGCCTGCCAGTTCCTGATGCATGCTACCTCCAGTCGATGT 5917

Si-tin1 1205

Sv-tin1 AGTGCTACCAATTATGGCCGTGAAGGCACGTGTCGCGACGCCATTGGAGCAATCCAGCTGTCCCATCGCCAGTGAGAATC 5997

Si-tin1 1205

Sv-tin1 TTGGCCTAAGAACCGGCCAATAGAGAAAAGCTTCTCGACATCTTGATTTGGCCTGCAAAAACTTTCATGGATCGATGATT 6077

Si-tin1 1205

Sv-tin1 ACACTTTTTGTTTTTGATGTTGTGTGCAGCCGAGCCCTTGCTGGTTGCATTGGTTCCTTCCGTACGTGCGCTTGTCGTTT 6157

Si-tin1 1205

Sv-tin1 TGCTTATGCAATCAATTTTATTGGGGGAAAATCGCAAAAATCTACCTATACAACCAACTTTCAAATAACGGTCTAACCCC 6237

Si-tin1 1205

Sv-tin1 TATGAAAGCTTTTATGCGGCTGTTGTTAGGTGCATGCTCTATGTATAAATGATTATGCCAAGCCTCATGTGCGAAGGGCG 6317

Si-tin1 1205

Sv-tin1 ATGCAAATCTAAGCATAATTTGCACTCCCGAGTTAATTCGAAAGATTAGTGCCTGATTAGTGATCACCAACGGCCGGCGA 6397

Si-tin1 1205

Sv-tin1 TCCTTGTCATGGAAAGGTGGTTGTCGCGATTGCGAGCTGAGCCGGCAGAGTCTAACATACTTCAGGTGATGTTCAGGTGA 6477

Si-tin1 1205

Sv-tin1 TGTAGTATTGTAGTTCAGGTAAAAATGTGCGAATTATACTGTGCTGCTCCAACTAGCATATATTAGTGTTTCTTGGATGC 6557

Si-tin1 1205

Sv-tin1 TAGCTAGTGCACTTTGCATATATGTCAAGGTAGTGGGGGCATCTCTTCTCCGAGCAAATCAAGCAACACAGAGTATATTT 6637

Si-tin1 1205

Sv-tin1 CCATGTACTGCTTTTAAGAAAGATTTGCTTGTTTGGTACACGAACATAAGGCTCTCAACAATTGTGACAAAATCACTATC 6717

Si-tin1 1205

Sv-tin1 AGTACTACAGAAATGGCTAGCTCCCAACCCTACATCACCGCGGGTTTGGGGTCCGGCGGATAAAGGTTGGCAGTTTGGGG 6797

Sv-tin1 CCCTCAACCCTGGATCACCGCCGGATAATAAACCAGCGGTGAAGCTTATTAGAGGAAAAAAAAAACCACATGTGCCCATT 6877

Si-tin1 1205

Sv-tin1 GTTATCCTAAACGCTAAACGGTAAATAGTCGGTCACTCTTTGTTTAGTGTTTAGATGGTTTAAATGGTGTTTTGACGGAA 6957

Si-tin1 1205

Sv-tin1 TTGAACGGTCTCAACAGTTTTGCACTTTATCACTAAATTATACATAATCATGCTCATAAAAGATCAAGTATTCTAGAAAG 7037

Si-tin1 1205

Sv-tin1 TATATATATTTATGCATATAAAAGACCAAGTACATATATTCATGCGTGCAACAAACCAAATATAAATATTATCTATGGAA 7117

Si-tin1 1205

Sv-tin1 AAACTTAAGTATATATATTTATGGAAGTAAGAATTCAAGTGGAATTGACAGTGTTCACCTAAACAGGATGTTAAATGGCC 7197

Si-tin1 1205

Sv-tin1 GTTTAGCCTGTATAATGATGTTTAATTCAGTTTTGTTTAGGCCATTTAGCCCGTTTAATCTTTTTTGACCGTTTAAACCC 7277

Si-tin1 1205

Sv-tin1 ATTTCCATTTAATGGACCGTTTATAGCCTAAACAAAACAGTTGGTCTCCGTTCACTGTTTAGACGCCGTTTAAATGGCCG 7357

Si-tin1 1205

Sv-tin1 TCCGTTTAGGCGAGCACTGCCTGTGCTGCTAGGGTGCACCGCCCGCCGCCCGTCGAAGTCCCATCACACGCCGCTGCACC 7437

Si-tin1 1205

Sv-tin1 TCATCCTCGCCGCGGCCGCCGCACCTTGTCCCTGAGCCTCGAGCTTGCCCCACGACCGCTACCGCACTTTGCCCACATGG 7517

Si-tin1 1205

Sv-tin1 CCCGCCATGCCTCGCCCCGTCGGATGCCGCTGCTGCAGCTCCTTGCCCCACCGACCGTCGTCGCTCCACATCTCTCCCAC 7597

Si-tin1 1205

Sv-tin1 CACATCTGCCACACATCGTCCCTGCGAATTCCCCCACTGGACGCCGACATGCCGCATCTCCTCTCGCTGCGGCTGCCGCA 7677

Si-tin1 1205

Sv-tin1 CTTGACCCACACGGACCGCCACGCCGCGCCTCGGCTATAGCCACCGAGCCTCCCACTGCGGCCATCGCACCTTGCCCTAC 7757

Si-tin1 1205

Sv-tin1 GGCCACCACCGCACTTTGCTCACGCGGGCCGCCGAGCCTCGCCCCGTCGGGCAACGCCGCGGAAGCTCCTCAACCCGCCG 7837

Si-tin1 1205

Sv-tin1 GCCGCTGCCACCGCTGCTCCTTGCCCTACTGCTGCTCATCACTACAGGCGTGAGCACCAGCTGCTCTACGCCAGTGAGGG 7917

Si-tin1 1205

Sv-tin1 GCCAGCGGAGGGGGGAGGAGAGGGGCAGGTAGCCGGCAAGAGTGAATGATTTTTTGGAGAGGGTGAGAAGTGGACAGAGC 7997

Si-tin1 1205

Sv-tin1 AGAGAGGGGATGCGGAGGATAGAGAGAGAGGGGGGAGGCTATCTGTGGATAAGATAGAGATGGATGGGAGATGTGGATGG 8077

Si-tin1 1205

Sv-tin1 ACTTGAAGGACTGGTATCAACTTTTTTTTATAAAGATTTCGGGTCTGCATACCCTTTCACCGTCGGATGGAAGTACAAAC 8157

Sv-tin1 TGGTATAAACGCCGCTCCATATTACAAACCGGTGGTGGTAGTCTTTTTCACTGCCAGTTTCAATAGCTAACAACCCCATC 8237

Si-tin1 1205

Sv-tin1 ATCTTTTGAACTGGTGGGGATACACTGTCACCGTCGGCTCACATATAACCGGCGGTGATAGTCCGGTGGTGATGGGCGGT 8317

Si-tin1 1205

Sv-tin1 TCTGTAGTGATATCTTTCATGGTTCTATAACAAATTGTCGGAGAACATATAGTACAAGTTTTTTAAGGTCTCTTGGTCTT 8397

Si-tin1 1205

Sv-tin1 AACTCCAAGATAGTTGATTCGAATGGATTGTTCAAAAGATCACAAAGACCAACAAGAAACAACAATGATTCATAATAACT 8477

Si-tin1 1205

Sv-tin1 GATGTTTTTTCATTCACTGCATAATATAGAAAAAAACACATTATTGCAATATAGTGATATGGATGCTCACACACAAAAAA 8557

Si-tin1 1205

Sv-tin1 ATCCGGATGAAAAATGTTGCTTCATCCAAGCACCAGTGGCCAACCATGAACCATGCACAAATGAACCATCTATAGACCGT 8637

Si-tin1 1205

Sv-tin1 ACATAAAATCTTGCTATCCAGGTCTTCCACCAAAGATATTTGGTGCCACTACTAGGCCTATCTATATAATTTACCTAATA 8717

Si-tin1 1205

Sv-tin1 TTGGATTCAATCTACATCACTTATTGACACCACCAATGTAGATGCCGAAACATGCTTTGAAGGTAGCAGGCACGTTTCCA 8797

Si-tin1 1205

Sv-tin1 TCTGCCCATCTGTCATTTTTCTTTTCGTTGTTGTCTACCTCCATGCCTGACCATGAAACATTTACCAAGCTCTAGGTTCG 8877

Si-tin1 1205

Sv-tin1 CACAAGCCCCAGTGCAACATGCCCTCCTTCGCATATGCTCCATCTGTTATGGGCGCCGCCACTTAATATCATCCATGCTT 8957

Si-tin1 1205

Sv-tin1 GTTGGCATTGACCTCCACAAGTGGGTCATCCAAAATGACGAGCATACCTCGCACCATCTCACCCACCTCCACTGGAGCCT 9037

Si-tin1 1205

Sv-tin1 TCACCTTCGTGAACTCTATCTCTAGAAATCTGCACCAAAACTCAGCAACAAATTTCCCAAGCAGCCAAAATCCACAGGTA 9117

Si-tin1 1205

Sv-tin1 GTTCTTTTGGACGAATTGTTGGTATTAGTACATACCTTCATGAAGTGGCAAATTCGTTATTGAAAGTCCTCGATGTCACC 9197

Si-tin1 1205

Sv-tin1 CTTCGACTTGTCAGGTGCCTGATCCTACTAGATTTTGTTGTTGTTGGCTCTACCGTGGTCGACACTAGGTGATGGTGGTG 9277

Si-tin1 1205

Sv-tin1 TGATGGCTTTGGATCTATAGGAAGAGAAACACTACCAAATACCACAGAAACTATTTGTCGAAGCACCTATTTTTTTCAGG 9357

Si-tin1 1205

Sv-tin1 AACAAATGAAAAGTTCATCCACCCCTACTCATTTCGAGGGGCGGTACAATTTTCAGGGACGGACAAAAAATGAACTGCCC 9437

Si-tin1 1205

Sv-tin1 TTGGAAATCAGATTTTCAGGGGCAGGCTGAAATATGGACCACACCAAAAATTCAATATCAAAATTTTAGCCACCGCGCGC 9517

Sv-tin1 CCTAGAAATTGAAAAGGCTACATAGGTGGTGGGACTGTGCCCTCTATTCTCTCTCTCATCTCTTCCTTTTCTCTCACTAA 9597

Si-tin1 1205

Sv-tin1 TCTCTTCCATCTCACTCACTTATCTCTTCCCCTCTCTCTTCTCTACCTGCCGAGCTCTCACTAATCTCTTCCATCTCACT 9677

Si-tin1 1205

Sv-tin1 CACTTATCTCTTCCCCTCTCTCTTCTCTACCTGCCGAGCGTCCCTGCTCATCCTCTCCATGGGGCATTGCAGGGCAGCAA 9757

Si-tin1 1205

Sv-tin1 GGAGGTCGACTCAACAGGGGTGATTCCTGTTTGATTAATTACACTTAATAGTCGGCAATATGCTCATTGACAACGAGTTT 9837

Si-tin1 1205

Sv-tin1 TCTATGATGTCTTCCTCAATCAGCCTTAGATATCTAGCTTTTTATTTTCAATTATATTTCTAGGAAAATGTACTTCTAAT 9917

Si-tin1 1205

Sv-tin1 TACATTTCTTGAAAAAGCAGTACTCCTAATTTATGACTTTGTCCTTGAAGAATCCTTGTGGTTATGATCAAACAAATGAT 9997

Si-tin1 1205

Sv-tin1 TGGTTGTCTCGTATGCATGTCCCCATATCTAGCTTGTTTGTGTGAGGAAACATGCCCACCTGAACGGTTGGACGATCCAT 10077

Si-tin1 1205

Sv-tin1 CCAGTGTTTCATGCTCTGATCGATTGATCCCTACACAAATATTATTCAAAAACCAGTCCAAATATCGCATGGTGTGGTCC 10157

Si-tin1 1205

Sv-tin1 TCCTTTATCCACGTCAAATTCGATGGTATTTGTAAGCAATTAGTCGCAAGCTTACAAACAACCTGGTTATCTCTGGACGC 10237

Si-tin1 1205

Sv-tin1 TTATATATCCAGGCCGACTTCTGGTTGTGCCAGTTAGAACATTGTTCAATTACTTCCAGAAGGCCTTGTGCAATGGAGTG 10317

Si-tin1 1205

Sv-tin1 CTCATCACCTAGTGCTCCACCACGAGCGGTGGCGGCGGACCTGTCCCTGTCGTTGGCATCGGTGACAGGCAGCGCCGCCG 10397

Si-tin1 1205

Sv-tin1 CCGGGGACGTGGGCGGCAAGAACGTGCGGCTGTACCCCTGCCTTTTCTGCGACAAGATTTTCCTCAAGTCGCAGGCGCTC 10477

Si-tin1 1205

Sv-tin1 GGCGGGCACCAGAACGCGCACAAGAAGGAGCGAAGCGCCTGCTGGAACCCCTACCTCTACGACGGCCACGCCGCCGCCGC 10557

Si-tin1 1205

Sv-tin1 TGTCACCGTCGCCGAGCCCTTCTCGGTCGCCGGCAGCGCCGCCACCATGTCCATCCCCACGCAGTCTCACGGTGGCCACG 10637

Si-tin1 1205

Sv-tin1 GCGAGGACGGCGCCGACGACGCCCCGATCTATCGGGCGCAAATGCAGCGACGGCGCGCCGTGCTTTTTGCGCCGGTGGGC 10717

Si-tin1 1205

Sv-tin1 GCCGCCGGGGCTGATGGCACGGCTGCGAGCTGCGATGGCACGATCGACATGCTCAACTGGGTCAGGGCCAGCAGCGCGGC 10797

Si-tin1 1205

Sv-tin1 GGCCGCCATGGCACTCTCATCTGGCGCCGGCGAGGATCTCGACCTCGAGCTGAGACTCTAGACGTACGACTGCATCAATG 10877

Si-tin1 1205

Sv-tin1 TGGCTCCGTTGGCATTAGGTATATTGTCGTCAACCCTGTATACATGCTAGCTAAGTACCTATAGCTGAAGCCTCAACCAC 11037

Si-tin1 1205

Sv-tin1 AGTTTTTTTATGAGCTGTTGCAGGTTTTTTCAGTTGTATAATGTGTTTCAAGAAATCTAAAATAAACACCCTGGTTTGCA 11117

Si-tin1 1205

Sv-tin1 ATACAATAAGAATAGTATGCACACACGCGTTTAACTGATTTTCGTATCTATAGTTAAATTTATGTTTAAAATAGGTGAGG 11197

Si-tin1 1205

Sv-tin1 ATATTTATGGGAGAATTTATGCACAAAACAAGATTATCATATCAATTTACACTAACAGTCGAGCCAAGTTTCTAAAGCGG 11277

Si-tin1 1205

Sv-tin1 GATTGTCTATAGGTTTATGATGGGTGGTTCATTCTCTAAACTCTAAATAAGAGAGAATGTTTATGGGATATACACAAAGC 11357

Si-tin1 1205

Sv-tin1 AAGATTATCATATCACGCTGGTGATTAATTGCGGGGCTAAATATTTTAAAATACAAATTGCACTTGCTTTTCATTTTCAA 11437

Si-tin1 1205

Sv-tin1 AATATCAATATTTCTGATAACTCTTCTAGGAATTTTTTCATTAATCGGAGTTTCTAGATCTCCTCTTTTCATGATATTGT 11517

Si-tin1 1205

Sv-tin1 GCAGATTTGTAGTTCTCAAAATCTTGAAAGATTCGTAAACTCCTTTTTTTTTCCCAAAACAAGACGGGACTCCCTTACCG 11597

Si-tin1 1205

Sv-tin1 ATCTGATTTTATATATTATACAGAAGGAAAACTGTGCAAACTAATTACAAAGAGAAAAGAAAAAATAGAAGGAAGGAAGG 11677

Si-tin1 1205

Sv-tin1 AAGGAAGGAAAAAGGAATAAAAAGAAAGAGCTTGCTAGGTGAGTGAAGCCAGTCGTGAAGAAGAGGTTTCTACAATATAG 11757

Si-tin1 1205

Sv-tin1 TGTTACATGCTGTGTATACTTATTTAAATTAGAGTATCTAGCTATTTTTTATCTTTCAATTGCCTTTCTCAACCATGATA 11837

Si-tin1 1205

Sv-tin1 TCAACGTCCACATGTGCTGTTTCCTTTCACGAGTTGTGTGGTGAAATAAGAATAAACTTTCAGTGATTGGATTTTAGTGA 11917

Si-tin1 1205

Sv-tin1 AGATTTTAAGAAAAGAATTTATACTAACACGAAATTACCACATATATAGGATCGCCAGTACTTGTTTATCAAACACATGA 11997

Si-tin1 1205

Sv-tin1 CCATGAATAGAATTTCTTCTACAGATGGCAACACACTAGCTGTGTGCGATAATATACGTAGGCACCATTCTCGAGGAGGA 12077

Si-tin1 1205

Sv-tin1 ATATACACTACTAATTAATCTGGATGTGATGGCCTTAGATCAAATCTTATAGACCATATTTAATCGGGCTAGTCTCAATG 12157

Si-tin1 1205

Sv-tin1 CACAGTTTCATTGTACTATTACAAAGACTGCAAATTTGGTAATTGTGCCTTATGAGTATTATGGGGATGAAACTCGTTCA 12237

Sv-tin1 TTTCATAAAACTCCGTCCTCTCTCTCTACTAAGTCTCAAAATTCTGATGTGGCATAAAAATTAATGTTGATGAAACTCTC 12317

Si-tin1 1205

Sv-tin1 TATGAAACTCCAACTGAGACTTGGTTCCACGTACGAGTCGTCAGTGCTTAAGTCATTAAGTGTTCTCTCCGTGCCGTGCA 12397

Si-tin1 1205

Sv-tin1 ACTTCCCGCTAGTTCGCCACTGCCCCCTCATCAATCCACTCCTAAGAAAACTCCGGTTACCATCCTAGCTGCACTGTTTC 12477

Si-tin1 1205

Sv-tin1 GATCTGCATTCGATTCTCTATTGCTACTGCACTCACCTAGCTATCTAGCTAGCATCCTGGCGTCCCCAGCTAGCTCTCTG 12557

Si-tin1 1205

Sv-tin1 TTGCCATGGACTCCTTTCAACATAGTCATGAGTTTTTGGATGAGGTGGAGATAGAGTCTGAGGATGACTTGTTCTCCAAC 12637

Si-tin1 1205

Sv-tin1 TTGGAAGCGGAAGAAGATAAGGACTTTGACAGTGCATTGTATGTGGATATACATTACATGTGTTAATAACCTGATTGTAG 12717

Si-tin1 1205

Sv-tin1 GTTGATTTATTAGTTACTCCTTTGGCATCCTTGGTCTTTGTGTATGATATGGATATATATATCATAGATTCGTAGTGCAG 12797

Si-tin1 1205

Sv-tin1 CCAAAGTGCAAGTCATTATTTATATTTGTTTAGTTTAATTTTCAAGTCGTCAAAGTTGATCAAATTTTGTTTGCTTTCAA 12877

Si-tin1 1205

Sv-tin1 TTAATTTTCTGAAGTAACTCGACTCATCTATAAAATACAATATAGACGCAACACTCACATACACGGGATAGGATTGATAT 12957

Si-tin1 1205

Sv-tin1 ACCTGGGTATTGAACTCACCATGCAAAAGTGTCTCACTTTGTTGATGGACATGTCGGCCACCACTAAAAAAATAACGATG 13037

Si-tin1 1205

Sv-tin1 TTAATTCCTAAAACGAATACAGAAAAATATGTGCACTCGCGCTAAGTCTAAGACTTGAATTTGGCTGCTCAGGTTTCACT 13117

Si-tin1 1205

Sv-tin1 GTAAGAAACCTAACTATAACTTATTGTAGCTTAGTTCACGGTGCATTCATAGGTCTTGATTTTGATTTCCGATGACAGCA 13197

Si-tin1 1205

Sv-tin1 TTTTCCGCACCACTCACAACTCCAGTCAATTAAAAAATATGAAAGGTAAGACCTTGTTTAGGACAGCTCCCGGTCGGGAG 13277

Si-tin1 1205

Sv-tin1 GCGGAGCAGGGAGAGCTACTTCTTTCGGCTCCACCGGCTCCTCCACTCAGCTAGTTGATTGTAGCGCCTTGGATTGTGTT 13357

Si-tin1 1205

Sv-tin1 TTGGTTTCTTGCAACTGTGCTGCACAGTGTCACTACAGTATCAGTGCGGATGAAACTGGGCCTTGTTTAGTTCCGAAAGT 13437

Si-tin1 1205

Sv-tin1 TTGGAGGTGCCAAATTACTGTTACATCACTGTAGCACACTATAGCGTTTCGTTTGTATTTATGAATTATTGTCTAAACAT 13517

Si-tin1 1205

Sv-tin1 TGACTAATTAGGCTCAAAAGATTCGTCTCGCAAAGTACAACAAAACTGTACAATTAGTTTTTAATTTCATCTACATTTAG 13597

Sv-tin1 TACTTCATGCATGTACCGTAAGTTTGATATGATGGGGAATCTTCTTTTTGCATAGTGCCAAAATTGGGAGTTGGGGATGA 13677

Si-tin1 1205

Sv-tin1 ACTAAACAAGGGCTACGGGGAGCTCTCCCAAACACAGCCTAATTACTGTTATTTGGCATTGTGTGGGACCTTTTTGCGCT 13757

Si-tin1 1205

Sv-tin1 TCCTACTTGGCTACGAGGACGTCCCCACCTGCATGGGCATATAAAAGCATAAGATTTCTAGAAGGAGTCATTGTGCCGTG 13837

Si-tin1 1205

Sv-tin1 CTTTAAAAGCATAAGATTTCTAGAAGGAGTCATTGTGCCGTGCTTTCGAGTAACCAATCAACAATGATAACACACAGACT 13917

Si-tin1 1205

Sv-tin1 GGTTGTACTATATTTTTTCCCTAGGGGAAATTTCTTGTTCTTTCTTTTTTAACGATTCGGATAGTGCGTGTTTTATTAAT 13997

Si-tin1 1205

Sv-tin1 ATAGCAGAAAAATATAAGACTACAGTCCTGGGAGGCCATAGGCGGGAAGGTCATAGGCAGGAAAAGGAAAAGAAAAAAAA 14077

Si-tin1 1205

Sv-tin1 AACTAAACAGACTCGTCCAGTTGGATTGGGACATCAACGCGAGTAGCCACGTAACTCAAAACTGCCACACCCGACCGGTT 14157

Si-tin1 1205

Sv-tin1 GGATTGGGATGTCAACACGGCCTCATCATCCACTCGCCACAGCTGCACCCACCAACATTCACGCTCATGTATTCGTCGAA 14237

Si-tin1 1205

Sv-tin1 ACCACCAAGCGTGGCCCTGCGTCGATGGGAATCGATAGAAGCAGATTGCACCGCACTGAGAAGGCCATCGCCATGCGGGA 14317

Si-tin1 1205

Sv-tin1 CCCTCCGCTGTAGTGCCGCTGCAACACCATACTCCACCTGACAGAGTGATACCACCGAATCCGGACCTCTTGCAGACTGC 14397

Si-tin1 1205

Sv-tin1 CTCGCAATCGTCACACGATAACACAACGCGCGGGGCCTCGCCACATCCGCCGTTGGACTCCACCTCGCTCTCGTCACCTC 14477

Si-tin1 1205

Sv-tin1 GAAGAACACCACACGCAGGGCCTCGCCACGCCCGCCACAGTACTCCATGTCACGGATCCATTTCTTTTGTCCCTGTCAGA 14557

Si-tin1 1205

Sv-tin1 GTGGTGAGCAATGTTGCCCCGCTCCACAAGATCTCCATCGATCAGCCAAGCCGTCGCCATAGGTTGACTTCGCCTCATTG 14637

Si-tin1 1205

Sv-tin1 CTTCACCCACGCAAAAGCATCCGCCGCCATGTCAGCTCTCCAAAGAAGTTGCTTGCGCCGTCTTCCGATGATGTACCGCA 14717

Si-tin1 1205

Sv-tin1 CCGTGTAGCCCAGCCAAGCTGAGCAACCCGCCGCGGTCCGCTGCAAGCCTAGTTGTCCCACGATGTCGTTGCCGCAAGCG 14797

Si-tin1 1205

Sv-tin1 TCGTCCTCCGACGCAATCTCACGCCGCACTGACAGTTGCCAAGCAACGCCAGCCGCCACGGTCCGCTGCAAGCAGCCAAA 14877

Si-tin1 1205

Sv-tin1 ACCGACAACCATGCGATAATCCCTAGCCAGCCACCACGGTCCACGTGGCCTCAGCAGCACCCGTCCAGCTTGCCACGTGG 14957

Sv-tin1 CGGAATTGTCGCCACCAGCTACGGCCGGCCATGACTAATGGACCAGCAATGCCATAGACCGAGATGTATCTTTAGAGACG 15037

Si-tin1 1205

Sv-tin1 ATGCCTGCAAGGAGGACACGGTGCTAATGGCGCCGCCATCGCTCGTCCAGAATCTAGACAGGGTTTTCACCCAGAGGACC 15117

Si-tin1 1205

Sv-tin1 TCGTGCAGTAGGGTTGTAGCTAAGACATGACACCCCTAACGGGGAGAACGACTCCCTAGGGAGCCGCCGTCATCTTCACC 15197

Si-tin1 1205

Sv-tin1 GCAAGAACACCGGTGAGAGCTTTTGCCCGGAGCATCCCAACCCCTGCACGCTCACGGCTCGGCACTCCCGGCCACAGTCA 15277

Si-tin1 1205

Sv-tin1 TGGCAACCCATCCGGGGCGGCGTCGCCACCGTGCCTTCTCGCACCACGCCGTCGTACCTCCACCTCCGCTCGCTCACCGG 15357

Si-tin1 1205

Sv-tin1 CAGTTATCCTCCTCATGCCGCGCCCTACACCGCTCCGCGTGTAGCTTGCGCGCAGCGCCTCCTCCGCGCCAGCACCATCT 15437

Si-tin1 1205

Sv-tin1 CCACGCCGCTGTCCTCCGCGCTGGCGTCACCTCCGCGCTGCACCTTGCGCGCAGTGCCTCCCCGGGCGCCACGGCCGCCG 15517

Si-tin1 1205

Sv-tin1 GCGGCCACCACGCCCCCTCCGCGCCGCCAGCCGCTGCGCCTCCTCTGGCGCATCCTGGCGCCTCGCAGCGAGGCCGCGCC 15597

Si-tin1 1205

Sv-tin1 TGCCGCAGGTGACCACGTGGCCCTACCGCTGCCGGTCGCCGCCGCCACCACTCGCCCGTCGCCGGCCGTCGTCCCCCGCG 15677

Si-tin1 1205

Sv-tin1 CCTAGGAGCCTCCTATCGGTGGATCCGGCCTCGGGGACACCAGATCCGCATCTGTCGGTGCCGGATCCGACAGCCCTCAC 15757

Si-tin1 1205

Sv-tin1 CAGTGCCACCACAGCCGCTCGTCCTCCGACGAGGCGGTCGCGCCCGCTGTATACGAAGGAGGAGGAAAAGACCGCCCCGC 15837

Si-tin1 1205

Sv-tin1 CGCCGCCGTCATTGTGGGCCGCTTGGACTTCCAGCAGCCAGCTCGAGTGGCGGTGCGGCGAAGGGAAGGGAGGAGGGAGG 15917

Si-tin1 1205

Sv-tin1 GGTGGAAGGCGGCGGCAGCGTTCGCCTGCCGGTGTCTTAAATACGCTTTCTCATAAAGCAGGGCGCCATTGTTCTACGGT 15997

Si-tin1 1205

Sv-tin1 CTAAGGGTTTTTTCCCTTGCGGTCCCATTTTCTTTAATCTTTTACTTTTAATACGACCCACCGTACGTATTTAGATACGG 16077

Si-tin1 1205

Sv-tin1 TGTATCTCCGTGGTACGTAGGTACTTGTGCTCAGGCACATATACGTGTCGGCATACATACGTACCTCTCTCGATTGGGCA 16157

Si-tin1 1205

Sv-tin1 ACCCCCTTCTCTCTGGTCATCTGTCAATTATTGGATGTTTTCCTGTCACAGTTACTGTTTGTGTCCTTGACGAATCATAT 16237

Si-tin1 1205

Sv-tin1 GTCGCTGTAATGGATAGTAGACATCAGATCAGTGGGCATATATTATTGGTTGTTTTCTGCTGATCTGCGCCGCCTTTCGT 16317

Sv-tin1 ACGTTGGATGGATAATCCCTTTTTCAGTGGACACAGTTAGCACTACTTGAGGACAAATCATGCATGGGTGCCTCTCTTGG 16397

Si-tin1 1205

Sv-tin1 CCACTGTACCTTCCGTCTTCTTCTCCCCTCTCTTTTCACTCCCAACCTAACCCCAAAATTTCCTTCTTGGCCATCTTGAT 16477

Si-tin1 1205

Sv-tin1 GTGAGCGTTCCTCGGTGATGACCCCGGAACGCCGTCCCGCCCCGCGAAAACAAACTGGGCTCTAGTATGGACACACAGGA 16557

Si-tin1 1205

Sv-tin1 TGAACATACTCGACTGATCACAGCTCGCTGGCTGCTCGATCGCATGGAAGGGCAGCCAGCTACAACAGCCGCATCCAAGC 16637

Si-tin1 1205

Sv-tin1 CTGCAGGATATATTGTTTTGTCCCCAGCGGGTAAGTAAAAGCCATCGATGTATAGAACAATGTCTAATCTAATTAGGACA 16717

Si-tin1 1205

Sv-tin1 AAACTGTACTATATGTCTACATGCCTAATCTTAATCTAATTAGGACACCACTGCACGCACCAAGAGCATTCTCCATTTTG 16797

Si-tin1 1205

Sv-tin1 TTTTACACATGGAGAATCCACTGAACTTTCTACGACATAGACAATGGTACCTAACTATTTTCTAAAACTAGAAATATTTC 16877

Si-tin1 1205

Sv-tin1 TTCTTGTCCCGTGTACTAAGAAAAACACAGGCGATAAGATCATTCTCAATGGGGTTTAATCCTCATTAAATTATGTGCCC 16957

Si-tin1 1205

Sv-tin1 ATCGGCAAATATGATGATGATGACATGACAAACAAGATATTTATGTGGATGGAGAAAAGATGAATTCATGAGGATGAAAC 17037

Si-tin1 1205

Sv-tin1 TAAACGTACGCTGTTTTCGAGACATTAAATATATTGAAACCCGCGTTTGAGAGTTATAGGGTTTCATTTCATCTCATTTT 17117

Si-tin1 1205

Sv-tin1 GTCCAATCCAGTGCATCGTAATTAATGTCTATGACTTGCCTATATGTAACAGCATTGGAGGTATGATTTCATCAATTAAC 17197

Si-tin1 1205

Sv-tin1 AAAGAATTATTTTGGATGGTGTGGCTACCTTGAAAACTATCCCGTAACACTTGCCATTCAGAATGGCCTAAAATGCTACA 17277

Si-tin1 1205

Sv-tin1 TCTCAAGGATCGAAGTTATCACTGTCAGATAGTACGGCTACATCCTCAATAACTAAAAGTTACATCATTAGACAATAAAG 17357

Si-tin1 1205

Sv-tin1 TTACTCAATGTAATAACTTTTATACACCAATAATGATGCATACAATTTAACTTATTATTGTTAAAATTTATAGGCTTTGA 17437

Si-tin1 1205

Sv-tin1 CCTTTAAAATCCTAATACAGAGCTTAGAAACGAACAAAGAGTAAAGATGGACGAGTCGCTGCTGGACCGGCCAGTGGCCT 17517

Si-tin1 1205

Sv-tin1 CCCAGTCACTGAAGGACAATTGAGGGAGGGCTCACGGAGGTTCATCATGGAGCGCTGCCCAGTGCGCTCGAGTTCTCGAC 17597

Si-tin1 1205

Sv-tin1 GTCTTCGTCGTCGTTGCTGTTCGCGGCTTCGCGCTAGCTAGCGCAGTGACGATTTGGAGCTTTACCTTTTAGCACCTTAT 17677

Sv-tin1 ACTTACAAAATACAACGTTAAATATGTCAGCCAACAAAGGTATAGCATAATAGTTACTCCCTCCGTTCCAAATTATAGCT 17757

Si-tin1 1205

Sv-tin1 CGTTTTAGCCTTCTTAGGTTCATGGATATTATTATGCATTTAGATATAATGTATGTCTAAATGCATAATAATATCTATGA 17837

Si-tin1 1205

Sv-tin1 ATCTAGAAAAATTAAAACGACCTATAATTTGGTTGGAACGGAGGGAGTAATAGATAACATGTGTAACGGATAAACTATAT 17917

Si-tin1 1205

Sv-tin1 ATATAATCACGATTTTAAAGTTAATATTGGTCAATGGATGTTATAGCTTTAATAAAGCTGTATATGTGTTGAGAGACCAA 17997

Si-tin1 1205

Sv-tin1 CTACTAATCATCTTTAATTTAACCTCTCCTGAATAACTTTCCATGTCACATAAGGAAAATGGAGAAAGAAGCGCCACACC 18077

Si-tin1 1205

Sv-tin1 CTTTAGTTGGTACGGTTGGAGATGCCCTAACTCTAATGAATCGAAGGGAGTAAATGAAGAGAATAATATCAAGGTACAGG 18157

Si-tin1 1205

Sv-tin1 GCCAAATCGATCCAAATGCAAGCTCACCTAATTGATCGCAACCAGTCATTAACCTCCGATCAAGAACATCCATAGATCAG 18237

Si-tin1 1205

Sv-tin1 GAATCATCACCATGTGCAAATATAGTGTCTCTGTCTGCAATTCTGCATTGTTCTCTCGCTGATAGATGGACCCTCTAGCT 18317

Si-tin1 1205

Sv-tin1 TTTTGCCGTGCGAGAGTAGCCATCAGCCCATCAGCGGTCCACAATGCATCTTTCGAGGCCCCGTACGACGTGCTGTGCGC 18397

Si-tin1 1205

Sv-tin1 AAACAGGATGGGTGCCACAGTGCAATCAATGTAAAAGCGTGAACCGGGCCCAGCATCAAATTTCAAATGCCCTTCCTCTT 18477

Si-tin1 1205

Sv-tin1 GATTCCAAATGACACACCAGTTGATTCAAATTATAACAATGAGACGACCTAAGTTAAGAGAAATCAATTCAAGCACTTTA 18557

Si-tin1 1205

Sv-tin1 GTTTTGAATGGCTAGAACTTTTCTTACTTATCTCTGACCAAGTTTTTAGGAATAATCACCAATATTTACGACATTCAATT 18637

Si-tin1 1205

Sv-tin1 TGTTTCATGAAACCTGCCATGAAATATGTTTTGATATGGTGTTATTTGGTATTACAGATGTTAATGTATATTTTAAAAAA 18717

Si-tin1 1205

Sv-tin1 ATATATAGTTAAAGTTAAAGCAATTTGACTTAAGGGCAAAACTAAATTGAACTACAATTTTGAACAGTGGGGTGTTTTTT 18797

Si-tin1 1205

Sv-tin1 CTATTAAGAAGAAAAGATATGTTTGAGTTGAGAAGACCTGAACCCTGAGCTGGTGAGGAAGTAGCACCCAACCATGTACA 18877

Si-tin1 1205

Sv-tin1 CCATCCCTCCCCACCAACCAAGGAAGGCTTGGTTTCTTGGACCATTCTTAGTTGGAGTTTCATCCTCGTTAAAGGAGATA 18957

Si-tin1 1205

Sv-tin1 ACACACAAAACTGTTGATATGGAAGAGAAGTTACGAGAAGATAGAGTAGATGAGTTCCATCTAGATATACCCGGTGCACA 19037

Sv-tin1 CTATTTCCATTGCATTGGAAATCAAATGAAACTCCATGAATGCATTTCGTTTCATCCCCACGCGTTTGGAAACCAAGTGA 19117

*********** *************** ******** ************* **********

Si-tin1 -----------------ATTTCACAACACCATACAACACTTTAA--ATGCTATGATAAGCCTATTTAATCGTGAAATGAA 1266

Sv-tin1 AATTCTACTAAGGTTTTATTTCACAACAGCATACAACACTTTAAATATGCTATGCTAAGCCTATTTAACTGTGAAATGAA 19197

****************** ************************** *** **** ** **********************

Si-tin1 ACCTTTGTATTATAGTGGTTATTTCATCCATAGTTATATTTCACTGTAACTGATATTGCTGCCTTGGAAACAATTAAATG 1346

Sv-tin1 ACCTTTGTATTATAGTGGCTATTTCATCCATAGTTATATTTCACT-TAAATGATGTTCCTGCCTTGGAAACAATTAAATG 19276

***** *** ***************** ************** ************************************

Si-tin1 AAACTTATCACTGAGAATGGCCTTAGTGTTTCAAACTAATAGACGGAATAAGGAGCTAAGTTTAATTCAGTCTCTCAGCT 1426

Sv-tin1 AAACTGGTCATTGAGAATGGCCTTAGTGGTTCAAACTAATAGAGGGAATAAGGAGCTAAGTTTAATTCAGTCTCTCAGCT 19356

***************************** ******************* **************** **** ******

Si-tin1 CTAGGAAATTAGGTGAGATATAGGCACTGAAAAGTGTCCTATTCAAAACAAAAGAGTCATAAACACAAAGAATTAACTTT 1506

Sv-tin1 CTAGGAAATTAGGTGAGATATAGGCACTGGAAAGTGTCCTATTCAAAAC--AAGAGTCATAAACACATAGAAGCAACTTT 19434

****************** ** **** ****** *************** *************** ******* *****

Si-tin1 GGCATGACTCTAGTTCCTAAAGTGGCTATGGCTCTTGCTTGTAATGATGGAGAATCAGCTTTTTAAATGGAGCTAATGTC 1586

Sv-tin1 GGCATGACTCTAGTTCCTGAAATGGCGATGGCTACTGCTTGTAATGATGGTGAATCAGCTTTTTAAGTGGAGCTGATGTC 19514

********** *********** ****** ***** ******************* ********** *************

Si-tin1 CTTGGAAATATGTTTGGCAAAACAGCTTTCCAGTGGCAATTTGGGTAATCTTAGACACAATTAGCATTTGTGGAAGGAGC 1666

Sv-tin1 CTTGGAAATACGTTTGGCAAAATAGCTTTTCAGTGACAATTTGGGTAATCTTAGAGACAATTAGCACTTGTGGAAGGAGC 19594

******* ** ******** ************* ***

Si-tin1 GGAAGCCAATGAAACCACTTTTTAGGGCTCCTTTCTT------------------------------------------- 1703

Sv-tin1 GGAAGCC-ATAAAACCACTCTTTAGGGCTCCTTCCTTTAGGGGGTGTTTGGATACCGCTTGCTAAACTTTAGCAGCTAAA 19673

Si-tin1 -------------------------------------------------------------------------------- 1703

Sv-tin1 GAACACCCTTGCTAAAAGTTGCTAAAGTTGAGTTGCTAAAGTTTAGCACTTTAGCAAGTTTTTGGTTGCTAAAACTTGCT 19753

Si-tin1 -------------------------------------------------------------------------------- 1703

Sv-tin1 AAAAGGTGGGGTGGACACCCTTTGCCCCTCATTATTGCTTGTCTGGTTAGCATTCAGTAAGGGCAAACAGGTCTTTATCC 19833

Si-tin1 -------------------------------------------------------------------------------- 1703

Sv-tin1 ACCTCATTAATGATGCTGTTTAGTAAGGATATCCAAACAGCCTTAACTAAACTTTAGCAACTAAAGTTTAGCAACTTTTA 19913

************************************* ***

Si-tin1 ------------------------------------TTAGTATAAGTCATTTTGAGGCTTTCAGGGGGCTTCTTGGGTGA 1747

Sv-tin1 GCTGCTAAAGTTTAGCAAAGTTATCCAAACACCCCCTTAGTATAAGTCATTTTGAGGCTTTCAGGGGGCTTCT----TGA 19989

****************** ****** *** *********** *** ***** ******* *************

Si-tin1 AGCTATTTTCTTTTTACCTGTTTGGCAGGACTTCACAAATACGTACCTAGTGGAGAAGCTGGTGGAGAAACCCT------ 1821

Sv-tin1 AGCTATTTTCTTTTTACCCGTTTGGTAGGGCTTCACAAATAAGTAGTTAGTGAAGAAGCTAGTGGAGAAACCCTACCAAA 20069

******** *********** **** ******************** **************** *******

Si-tin1 -ACCAAACAGGCCTTAAATAGCAGTGC----TCTCTGCCCCTATAAATATG----TACTTGCTAACCCTTG--TAGCCTT 1890

Sv-tin1 CACCAAACACGCCTTAAATAGTGGTGCTCGATCTCTGCCCCTATAAATATGTACTTACTTGCTAACCCTTGCATAGCCTT 20149

**** ******************** ******** *********************************************

Si-tin1 AATTAGGGAGTTCTTTTCTGTTGTTATTTGTGCTGCTTGCTATTGCTAGGAGCAAAAGGACTGCATGTCCAATTGCCCAT 1970

Sv-tin1 AATTTGGGAGTTCTTTTCTGTTGTTGTTTGTGCTACTTGCTATTGCTAGGAGCAAAAGGACTGCATGTCCAATTGCCCAT 20229

***************************************************************************** **

Si-tin1 CACCTAGCTGAAGTAATAGTACTACACACACATCTCACCGCTAGCAAAGCGTCTTGCTCTCCCATTCGTCCTAGCTCTTC 2050

Sv-tin1 CACCTAGCTGAAGTAATAGTACTACACACACATCTCACCGCTAGCAAAGCGTCTTGCTCTCCCATTCGTCCTAGCTCCTC 20309

* ********** * * * *************************************************

Si-tin1 C---TTCTGGACAA-------CTG-----AGCTCCAGTTTTAGCAAAGCCAACAAGCTAGATTGGCCGGGGTCTGACAAC 2115

Sv-tin1 CCATTTCTGGACAAAGAATCGCCGCCACCATCTCCAGTTTTAGCAAAGCCAACAAGCTAGATTGGCCGGGGTCTGACAAC 20389

********************************************************************************

Si-tin1 TGAGCTGACTCAACAATTTATTTGCCAGTACAACTAAAACTACTCTACACACAGCCCAGCTTGTTGGCTGTGGTACTCTT 2195

Sv-tin1 TGAGCTGACTCAACAATTTATTTGCCAGTACAACTAAAACTACTCTACACACAGCCCAGCTTGTTGGCTGTGGTACTCTT 20469

*******************************************************************************

Si-tin1 CCTGGAACAGTTTGCACGGCGACTGGCTGCTTTTGCATGGAGCGGACCTTGTCGCCGCCATCGCCAGCTGCAGCGGCGCA 2275

Sv-tin1 ACTGGAACAGTTTGCACGGCGACTGGCTGCTTTTGCATGGAGCGGACCTTGTCGCCGCCATCGCCAGCTGCAGCGGCGCA 20549

********************************************************************************

Si-tin1 CCTGATGCTGTCCCTGGCGCCGGCGCCCGGACGCCGCGAGGAGTTGGAGTTGGACGACGAAGTGGCGGCGCCAGCGGCGT 2355

Sv-tin1 CCTGATGCTGTCCCTGGCGCCGGCGCCCGGACGCCGCGAGGAGTTGGAGTTGGACGACGAAGTGGCGGCGCCAGCGGCGT 20629

********************************************************************************

Si-tin1 ACGTCGTCGCCGGGAAGGAGGTGCGGATGTTCCCTTGCCTCCTCTGCAACAAGAAGTTCCTCAAGTCGCAGGCGCTCGGG 2435

Sv-tin1 ACGTCGTCGCCGGGAAGGAGGTGCGGATGTTCCCTTGCCTCCTCTGCAACAAGAAGTTCCTCAAGTCGCAGGCGCTCGGG 20709

********************************************************************************

Si-tin1 GGCCACCAGAATGCGCACAAGAAGGAGCGCGCCGCCGGCTGCTGGAACCCCTACGTCTACGCCGCCCCCGGCATCGGGAT 2515

Sv-tin1 GGCCACCAGAATGCGCACAAGAAGGAGCGCGCCGCCGGCTGCTGGAACCCCTACGTCTACGCCGCCCCCGGCATCGGGAT 20789

******************************* *********************************** ************

Si-tin1 CGCCGCCGCCGCCGCTATGTCCCTCCCCCCCAGCCTCGCACAGCGGCACTGCCGCGGAGCCCCTCGCTGGCGTCAAGCTC 2595

Sv-tin1 CGCCGCCGCCGCCGCTATGTCCCTCCCCCCCCGCCTCGCACAGCGGCACTGCCGCGGAGCCCCTCGCCGGCGTCAAGCTC 20869

************** *****************************************************************

Si-tin1 GAGAGGCCGGACACTTTGGCCATGATCAACTGGAGAAGGATCTCATGCGCCTCTGCTCCGCCGGAGAGCGCGCCAACGCC 2675

Sv-tin1 GAGAGGCCGGACACGTTGGCCATGATCAACTGGAGAAGGATCTCATGCGCCTCTGCTCCGCCGGAGAGCGCGCCAACGCC 20949

********************************************************************************

Si-tin1 AACACCGCGGCCTCCGGCGCCGTCGAGGAGCTGGAGCTGGACCTCGAGCTGCGACTCTAGATCGGCCGAGGATGCATGTC 2755

Sv-tin1 AACACCGCGGCCTCCGGCGCCGTCGAGGAGCTGGAGCTGGACCTCGAGCTGCGACTCTAGATCGGCCGAGGATGCATGTC 21029

********************************************************************************

Si-tin1 CTCCTTCCTTCCTTGACACCACGCAGGGGGCATCAACAACGAATTATCACTCTCTTCTTGGACCAGTAAGGCAGAAAATT 2835

Sv-tin1 CTCCTTCCTTCCTTGACACCACGCAGGGGGCATCAACAACGAATTATCACTCTCTTCTTGGACCAGTAAGGCAGAAAATT 21109

***************** ************************** ******* ***************************

Si-tin1 AAAGCTTGAAATGGATCCCTCTAATTACCTAGCCTGCTCTCTGTTAATGGATCCATGTGCAGTGCCCCAGTGCGGCAGTG 2915

Sv-tin1 AAAGCTTGAAATGGATCTCTCTAATTACCTAGCCTGCTCTCTGTGAATGGATGCATGTGCAGTGCCCCAGTGCGGCAGTG 21189

********************************************************************************

Si-tin1 CCCATCAGGTGTTGTTGGCGGCGGTGTATCAGTTGTCAGCGTGAGTCTACAATGCTGCAATATAATGTTCATATATATAT 2995

Sv-tin1 CCCATCAGGTGTTGTTGGCGGCGGTGTATCAGTTGTCAGCGTGAGTCTACAATGCTGCAATATAATGTTCATATATATAT 21269

********************************************************************************

Si-tin1 GAAATATGAAACTTTGGTACTGAAAATAAATTTGTATTTTTATGCATAGGAAAAGACTGTCAGTTGTTAGTTGCCCCCGG 3075

Sv-tin1 GAAATATGAAACTTTGGTACTGAAAATAAATTTGTATTTTTATGCATAGGAAAAGACTGTCAGTTGTTAGTTGCCCCCGG 21349

*********************************** ***************************

Si-tin1 GTTCAGTGGAGCTCATATTTTATGTTGCTCATATTGCCTGTTCCCTAGAAATAAAGGAAATGA 3138

Sv-tin1 GTTCAGTGGAGCTCATATTTTATGTTGCTCATATTTCCTGTTCCCTAGAAATAAAGGAAATGA 21412
