# Supplementary material for: The tin1 gene retains the function of promoting tillering in maize
Source: Nat Commun. 2019 Dec 6;10:5608. doi: 10.1038/s41467-019-13425-6 (PMC6898233; doi:10.1038/s41467-019-13425-6)
Supplement: Supplementary file 2 — Peer Review File [file 41467_2019_13425_MOESM2_ESM.pdf]

Reviewers' comments:

Reviewer #1 (Remarks to the Author):

The maize tin1 gene recovers tillering against domestication

Review

The paper reports on the discovery of a gene in popcorn and sweet corn varieties of maize that confers limited tillering, in seeming contradiction to the general trend of reduction in branching accompanying domestication. Transcripts derived from the different alleles of this gene persist for differing times and thus differentially affect downstream gene expression, leading to differences in bud elongation in field and sweet corn. The authors also investigate synteny of the gene region amongst cereal domestications and examine whether tin1 is under selection in domesticated species. The methods used by the authors are in general adequate. However, I have real issues with the intellectual framing of the study, and the lack of attempt to put this study in the context of what we know about domestication and improvement in maize. I organize my comments by section below.

Title
The title of this ms is confusing, as it implies that domestication occurred first, and then selection on the tin1 gene recovered some of the branching. However, the ms makes no attempt to examine the order of genetic events, there is no phylogeny to show how popcorn, sweet corn and field corn are related, and no reference to any of the excellent work that has been done on this subject.

Abstract
The Abstract also contains the assumption that sweet corn and popcorn are derived with the corn lineage, whereas popcorn is in fact one of the oldest domesticated forms of maize.

Introduction
The Introduction is exceedingly short, and describes the domestication story of maize and compares it with that of other cereal crops. However, the Introduction makes no attempt to describe what we already know, makes no mention of tb1, gt1, and tru1, well-known maize domestication genes that affect branching, and also no mention of sugary1, known to affect tiller number in sweet corn (Kebrom TH, Brutnell TP. Tillering in the sugary1 sweet corn is maintained by overriding the teosinte branched1 repressive signal. Plant Signal Behav. 2015;10(12):e1078954. doi:10.1080/15592324.2015.1078954). These are essential to the story of how tin1 may act to affect tillering, and their absence from the Introduction is a problem. Tb1, gt1, and tru1 are introduced in the results, but they should be also described in the introduction.

Results
- Please provide more information about creating NILs – number of generations etc.
- The authors mention that the NILs were generated from a residual heterozygous line, that was heterozygous at tin1 and mostly homozygous elsewhere. But was it homozygous in the regions on the chromosomes 4 and 5 that were associated with tillering? Or indeed homozygous for tb1, gt1, tru1, and sugary1?
- Page 6, line 122 "The tin1 gene contains two exons and one intron in its 5'untranslated region" – what does this mean?
- The TIN1-GFP fusion protein figure is indistinct, I can't be sure where it is localized, partly because the image is unclear.
- In Supp Fig. 10, it would be good to have antisense and sense in-situs together so that one can see the difference in the buds – I actually can't see any buds in the sense section.
- Page 9, line 180 – this is the first mention of tb1, the most well-studied gene for tillering in any grass species, and especially in maize. Reference to tb1 and some of its relevant literature needs to be in the Introduction!

- Laba1 – this gene controls awns production in rice, why was it examined in the context of tillering in maize – there is no mention of why this gene was chosen, and no justification.
- Yeast 2-hybrid assay is not explained well
- Tin1 selection assay – there is no explanation for why the authors conclude that tin1 has been under parallel selection in opposite directions across different cereals. This would be an important finding if well-supported.

Discussion
- I agree with the authors that they have evidence for a role for tin1 in repressing bud dormancy and for stimulating growth
- It is really not clear to me that the authors have evidence for a 'de-domestication' of maize with the tin1 mutation, regardless of the percentages of tin1 in the different types of maize. The stacking of mutants could have gone the other way, especially because we know that the popcorns were the earliest domesticated.
- It is surprising that the foxtail millet results and indeed all comparative syntenic analyses that were presented in the Results are absent from the Discussion.
- In many ways the Discussion seems very thin, both in terms of discussing their own results and in drawing conclusions in relation to other known genes and gene pathways.
- Again, what is the rationale for the choice of genes they discuss (LABA1 and the topless genes) and those that they don't (sugary1, and any in-depth discussion of tb1/gt1/tru1).

Summary
The tin1 gene appears novel, and to act as a enhancer of tiller growth in maize. It is a valuable addition to our knowledge on branching in grasses. However, the authors fail to put their work in context or to adequately discuss tb1/gt1/tru1, or discuss at all relevant literature on other genes isolated from sweet corn such as sugary1. The idea of a de-domestication of maize, while novel, is not convincingly supported, or even well-tested, by their analyses. In its present form, it is unlikely to influence thinking in the field of branching regulation.

Reviewer #2 (Remarks to the Author):

In this manuscript, Zhang et al analyze a tillering trait associated with sweet and popcorn maize. By QTL analysis and use of RILs they identified a single C2H2 zinc finger transcription factor, TIN1, as a likely candidate. By overexpression they showed that they can increase tillering in B73 maize. The molecular cause is narrowed down by association analysis to a single SNP in the 5'UTR that produces a splice variant in intron1. This variant is shown to increase stability of the transcript, so the model goes that increased TIN1 transcript stability promotes tillering in sweet and popcorn maize. The TIN1 TF has a repressive EAR motif at the C-terminus and they show that TIN1 interacts with maize TPL proteins, likely functioning as a transcriptional repressor. By transient luciferase assay and RNA-seq analysis they identified possible direct targets of TIN1 repression, among which is GT1, a domestication tillering gene in maize. Given the importance of tillering in grass domestication, by broadening the analysis of TIN1 to other grass species they found association between TIN1 and tillering in foxtail millet and sorghum and evidence of selection in maize, sorghum and foxtail millet.

Tillering is an important agronomic trait in several crop species and understanding its regulation is crucial for the modification of plant architecture. This manuscript presents a large amount of data and work, and it presents very interesting results. However, there are several concerns that need to be addressed.

Main concerns:
1) The logic for the overexpression experiment is missing. It makes more sense if presented after

presenting the splice variant analysis, not before.
2) The authors present transformation lines in the maize inbred background B73 but do not provide any details of how this transformation was obtained. B73 is notoriously recalcitrant to transformation and this is by no mean a simple task. Details on the transformation protocol must be provided.
3) The analysis of TIN1 transcripts in B73 should be provided. The expectation is that in B73 the relative contribution of the tin1.1 spliced variant should be lower than in P51.
4) The in situ results in Fig4 are not convincing. Arguably, this signal could be simply background noise.
5) Lines 228-239: The authors re-named incorrectly all TPL/TPR proteins of maize. Use the nomenclature previously published (see Liu et al Plant Phys 2019). Your nomenclature does not match the phylogeny classification (i.e. TPL2/REL2; Arabidopsis does not have a clear REL2 ortholog). Also, there are four TPL-like genes in maize called REL2/RELK1-3, all expressed ubiquitously (not three). It would be very surprising that one of the 4 members is not expressed in buds (line 231).
6) Line 300: "Maize tin1 responded to light stimuli.." No evidence is provided that this is the case.

Other concerns and suggestions:
7) Modify Figure 3C, add entire gene model. As presented it looks like the intron retention is occurring in the coding sequence, not 5'UTR and it is confusing to the reader.
8) Be consistent with nomenclature between text and figures, tin1.1 or tin1-1, do not switch between the two.
9) Line 231: change "proteins" in "genes"
10) Move lines 334-339 into Result section
11) Line 115: correct "self-crossed twice"
12) Provide details on the inbred line used for the protoplast assay.
13) Lines 295, 342: delete "Our"
14) Add labels to Figure 1 plants (i.e. sweet corn etc.)

Reviewer #3 (Remarks to the Author):

This manuscript describes the positional cloning of a QTL that increases tiller number in sweet corn. QTL and fine mapping identified a single gene, tin1, encoding a C2H2 zinc finger protein that likely acts as a transcriptional repressor. This gene is an ortholog of the previously identified rice QTL prog1 which promotes an upright growth habit. A single nucleotide polymorphism at a 5' splice site of tin1 was found to inhibit splicing of a 5' non-coding intron, resulting in a decreased level of RNA decay for the altered transcript. Increased expression of tin1 leads to a decrease in the known tillering regulator gt1, but has no effect on the other major tillering domestication locus in maize tb1. Tin1 interacts physically with maize orthologs of TOPLESS, via the conserved EAR domain. Transcript profiling of tin1 shows that multiple hormone pathways are targeted, as well as photosynthetic machinery. The authors show that tin1 has been selected in parallel in setaria and sorghum as both have QTL for tillering that map to the tin1 ortholog. The setaria QTL has a deletion that includes 2/5 copies of a tandem duplication. Finally, they argue that tin1 was selected for increased tillering in sweet and popcorn maize lines by introgression from standing diversity in teosinte.

The positional cloning and partial characterization of this maize tillering QTL represents an impressive amount of work, and the authors present a convincing case that they have identified the correct gene. The case for the splice site mutation as causative is interesting, but not yet completely convincing (see below). Even without an airtight case that they have identified the causative polymorphism, this work is likely to be of broad interest as it identifies a new gene regulating tillering in maize, and likely in other closely related panicoid cereals. Beyond that, tin1 is a QTL regulating a distinct domestication phenotype in rice and thus provides another example of how variation at a small subset of genes has been selected in parallel, sometimes for similar and sometimes for divergent phenotypes.

While I am generally excited about this work, I have several concerns about the characterization as it currently stands. I detail my major concerns below followed by other minor comments.

Major concerns:

1. The splice site mutation is an intriguing possibility for the cause of the phenotype. However, at this point, other polymorphisms in the promoter (in LD with the splice mutation) could also contribute. The primary evidence against the promoter mutations as causative is a luciferase assay of the promoters from the parents tested in maize protoplasts. While this assay shows no significant difference in expression, that does not mean the promoter polymorphisms are insufficient for differential expression in a different tissue context. Promoters are highly context dependent, and that is likely to be the case for tin1, as shown in S9. Indeed, the expression results presented in S9 provide additional reasons to be skeptical that differential splicing causes overexpression of the P51 allele. It is clear from S9 that overexpression of the P51 allele is not global, and both alleles are equally expressed in leaf and root, while the differential expression is unique to the tiller bud and SAM. Why wouldn't this splice mutation lead to reduced RNA decay in all tested tissues? A simple confirmation that the splice site is indeed altered would be to sequence the alternate transcripts. A follow up would be to test the relative abundance of each transcript in the various tissues.
Sequence analysis of the alternate transcripts would still not confirm the SNP as causative as that would require ruling out the promoter SNPs by recombination or a comparison of transgenic lines that differ only presence of the SNP. I understand that this is a significant amount of additional work, and I am not arguing that it is needed for publication, only needed to make a strong argument that you can rule out the promoter polymorphisms.

2. The final section of the Discussion introduces novel data not presented in the results arguing that the sweet corn tin1 allele originated from introgression from teosinte. At least I think that is the argument. However, a much stronger population genetic analysis would be needed to argue this. It is clear that the splice site mutation is present in both maize and teosinte, but that is true for most alleles in maize. Furthermore, the splice site allele is not fixed in either sweet corn or popcorn, and is often present in other maize lines (although more common in sweet corn). This does not seem to me like the kind of pattern you would expect for strong selection of tin1 after introgression from teosinte. Indeed, the reduced polymorphism seen for the splice-site allele (fig 6e) is exactly what you would expect if you select just one allele. I'm not an expert in tests for selection, but this doesn't seem to include the proper comparisons to make the case that selection is involved. It shows only that polymorphisms in a region including the promoter and first intron/exon of tin1 are in LD, that could be due to selection, or to other factors (e.g. reduced recombination).

3. The in situs in figure 4I are indistinguishable from background. These should be removed entirely or some convincing localization should be presented.

Minor comments

1. The English is hard to follow in places and the entire manuscript would benefit from careful editing by a native English speaker.

2. Pg 3. Line 58-64, also pg. 16 line 343-345. Tillering is increased in sweet and popcorn, but this study only looks at one line of sweet corn. It is not clear that tin1 is important for tillering in popcorn, or even for all sweet corn lines as it is not fixed in sweet corn.

3. Pg 5 line 96. The transformation presented in this section is not a "complementation analysis" as no LOF phenotype was complemented by introduction of a WT copy. Rather this tests if overexpression of tin1 is sufficient to cause a tillering phenotype, which it is.

4. Pg 8 line 166-9. TIN1-YFP shows localization to both nucleus and cytoplasm, I was not convinced

that it was “mainly” in the nucleus. Expression in the nucleus is not sufficient reason to conclude it is a TF, but in combination with the other sequence features is consistent with that conclusion.

5. pg. 9 line 183 to 185. I’m not sure that you can make a statement that tin1 acts in parallel to tb1 with the data at hand. A strong case for parallel action would require an analysis of a tin1-lof tb1-lof double mutant. The data right now only indicates that tb1 does not regulate transcription of tin1 and tin1 does not regulate transcription of tb1. There could be genetic or physical interactions between tin1 and tb1 beyond a direct transcriptional interaction, but these were not tested.

6. Pg 12 line 261. There are only 6 copies of tin1 in setaria, and there is not SiTin1-6 in the phylogeny.

7. Pg 13 line 289. Cannot say that tin1 corresponds to the tillering QTL in sorghum, only that it is linked.

8. pg 14 line 298-301. While gt1 has been shown to respond to R:FR ratio during tillering, there is no evidence that tin1 is in the SAR pathway. Rather tin1 regulates gt1, i.e. it regulates a downstream effector of the SAR. There is no data presented that tin1 responds to light stimuli.

9. pg 15 line 315-326. The argument that tin1 was selected to increase the ears and thus yield of sweet corn and popcorn doesn’t make much sense. If tin1 was selected for increased yield in these lines, why was it not selected for a similar yield increase in non-sweet or non-popcorn lines? Why would it only increase yield in these specialized lines?

10. Is there only one tin1 copy in maize, while other cereals all have multiple tandem duplicates? If so that seems strange, and worthy of some discussion.

Dear Editor,

We thank you for your consideration and time. We also thank the three anonymous reviewers for their critical review of the manuscript and detailed comments. All comments, major or minor, were given careful consideration before we proceeded to revise the manuscript accordingly.

Please see our point-to-point response to these comments.

Sincerely,

Zhongwei Lin
==

Reviewers' comments:

Reviewer #1 (Remarks to the Author):

The maize tin1 gene recovers tillering against domestication

Review

The paper reports on the discovery of a gene in popcorn and sweet corn varieties of maize that confers limited tillering, in seeming contradiction to the general trend of reduction in branching accompanying domestication. Transcripts derived from the different alleles of this gene persist for differing times and thus differentially affect downstream gene expression, leading to differences in bud elongation in field and sweet corn. The authors also investigate synteny of the gene region amongst cereal domestications and examine whether tin1 is under selection in domesticated species. The methods used by the authors are in general adequate. However, I have real issues with the intellectual framing of the study, and the lack of attempt to put this study in the context of what we know about domestication and improvement in maize. I organize my comments by section below.

Question 1(Q1):
Title
The title of this ms is confusing, as it implies that domestication occurred first, and then selection on the tin1 gene recovered some of the branching. However, the ms makes no attempt to examine the order of genetic events, there is no phylogeny to show how popcorn, sweet corn and field corn are related, and no reference to any of the excellent work that has been done on this subject.
Answer 1 (A1):
Phylogenetic tree based on whole-genome SNPs (Supplementary Figure 18b) from maize HapMap III revealed that sweet corn and popcorn were closer to wild progenitor teosinte than field corn. This result suggested that both sweet corn and popcorn was the oldest domesticated forms of maize, which was mentioned by Reviewer#1. Based on these descriptions, we changed the title with " The tin1 gene retains tillering in maize". We added a sentence to show the phylogenetic tree (P19L 414-416):

Phylogenetic tree based on genome-wide SNPs revealed that sweet maize and popcorn seem to be early domesticated types (Supplementary Fig. 18b).

Q2:
Abstract
The Abstract also contains the assumption that sweet corn and popcorn are derived with the corn lineage, whereas popcorn is in fact one of the oldest domesticated forms of maize.
A2:
We agree with Reviewer#1 that popcorn and sweet corn are the oldest domesticated forms of maize. So we rephrased several sentences in the abstract and deleted “against domestication” in the manuscript:

...however, sweet maize and popcorn retained a tillering growth habit during maize diversification. The molecular genetic mechanisms underlying how maize retains tillering remain unknown. Here we showed that the retention of maize tillering is controlled by a major quantitative trait locus (QTL), tin1...

Our results supported that maize tin1, derived from a standing variation in wild progenitor teosinte population, retains tillering and thus might increase ear number and yield in sweet maize and popcorn.

Q3:
Introduction
The Introduction is exceedingly short, and describes the domestication story of maize and compares it with that of other cereal crops. However, the Introduction makes no attempt to describe what we already know, makes no mention of tb1, gt1, and tru1, well-known maize domestication genes that affect branching, and also no mention of sugary1, known to affect tiller number in sweet corn (Kebrom TH, Brutnell TP. Tillering in the sugary1 sweet corn is maintained by overriding the teosinte branched1 repressive signal. Plant Signal Behav. 2015;10(12):e1078954. doi:10.1080/15592324.2015.1078954). These are essential to the story of how tin1 may act to affect tillering, and their absence from the Introduction is a problem. Tb1, gt1, and tru1 are introduced in the results, but they should be also described in the introduction.
A3:
Based on Reviewer#1's suggestion, we added a paragraph to introduce the cloned genes for tiller development in maize. As for suguary1, we also identified this gene in our RIL population derived from a sweet corn and a maize elite inbred line. The gene suguary1 was located on the short arm on chromosome 4 and the gene is far away from the QTL for tiller number on chromosome 4 (160cM-175cM). Thus sugary 1 is not responsible for tiller number in sweet corn (Supplementary Figure 1 e and f). This paragraph was shown in the following (P3-4 L 64-74):

The development of maize tiller is controlled by a complex gene regulatory network. A critical gene, teosinte branched1 (tb1), encoding a TCP domain, was responsible for the key change from the teosinte plant with multiple braches to maize with a single stalk (Studer et al., 2011). The gene grassy tillers1 (gt1) with a homeodomain protein can response to shade, which depends on tb1. The gt1 gene functions as a tiller repressor, and high gt1 expression suppresses the outgrowth of tiller buds (Whipple et

al., 2011). tassels replace upper ears1 (tru1), encoding an ankyrin-repeat domain and directly targeted by tb1, also plays as a tiller repressor. High tru1 transcription suppresses the outgrowth of maize axillary bud (Dong et al., 2017). In sweet maize, *sugary 1* was supposed to change the carbohydrate metabolism balance in favor of tiller bud outgrowth (Kebrom et al., 2015). However, whether other genes contribute to maize tiller development remains little understood.

Q4:
Results
- Please provide more information about creating NILs – number of generations etc.
- The authors mention that the NILs were generated from a residual heterozygous line, that was heterozygous at tin1 and mostly homozygous elsewhere. But was it homozygous in the regions on the chromosomes 4 and 5 that were associated with tillering? Or indeed homozygous for tb1, gt1, tru1, and sugary1?

A4:
We applied a residual heterozygous line (RHL, F6) to generate NILs. To check whether the six loci of tb1, gt1, tru1, sugary1 and two additional QTL of tiller number on chromosome 4 and 5 are homozygous, we rerun genome-wide alignment to identify SNPs between the two NILs based on RNA-seq, which was performed between the two NILs derived from this residual heterozygous line (F6) for the gene network of tin1. The genotypes from these two NILs were finally combined to generate the genotype of this RHL (F6). All six loci of tb1, gt1, tru1, sugary1 and two additional QTL of tiller number on chromosome 4 and 5 are homozygous. These data was shown in the Supplementary Figure 1e and f.

Based on these descriptions, we added a sentence in Methods:

The two NILs were derived from the self-pollination of a RHL (F6), harboring a heterozygous genomic fragment at tin1 and homozygous genotypes at most other loci (Supplementary Fig. 1e and f).

Q5:
- Page 6, line 122 “The tin1 gene contains two exons and one intron in its 5’ untranslated region” – what does this mean?

A5:
This sentence means that tin1 gene has two exons and one intron, and this intron is located in its 5' untranslated region. To describe this clearly, we rephrased this sentence in the following (P6 L118-119):

The tin1 gene contains two exons and one intron. The intron is located in its 5' untranslated region (5' UTR).

Q6:
- The TIN1-GFP fusion protein figure is indistinct, I can’t be sure where it is localized, partly because the image is unclear.

A6:
We introduced the TIN1-GFP fusion protein into maize leaf protoplasts again. We can see the signals of this fusion protein were present in both nucleus and cytoplasm. We then replaced Fig. 4K with new picture in the revised MS.

Q7:

- In Supp Fig. 10, it would be good to have antisense and sense in-situs together so that one can see the difference in the buds – I actually can't see any buds in the sense section.

A7:

In situ hybridization, real-time qPCR and RNA-seq revealed that the tin1 gene has a very low expression level in tiller bud. We also conducted in situ hybridization for tin1 again. The signals of tin1 still can not be clearly distinguished from background. Thus we removed in situ hybridization in the revised MS.

Q8:

- Page 9, line 180 – this is the first mention of tb1, the most well-studied gene for tillering in any grass species, and especially in maize. Reference to tb1 and some of its relevant literature needs to be in the Introduction!

A8:

Thank you for the suggestion. In Introduction, we added a paragraph to introduce these tiller genes in maize.

Q9:

- Laba1 – this gene controls awns production in rice, why was it examined in the context of tillering in maize – there is no mention of why this gene was chosen, and no justification.

A9:

In rice, Laba1 (also named An-2) controls awn, tiller number and yield. In An-2 paper, An-2 was shown to be responsible for tiller number.

To describe this point clearly, we added a sentence:

In rice, Laba1[20] (also named as An-2[21]) controls awn, tiller number and yield.

20. Hua, L. *et al.* LABA1, a Domestication Gene Associated with Long, Barbed Awns in Wild Rice. *Plant Cell* **27**, 1875-88 (2015).
21. Gu, B.G. *et al.* An-2 Encodes a Cytokinin Synthesis Enzyme that Regulates Awn Length and Grain Production in Rice. *Molecular Plant* **8**, 1635-1650 (2015).

Q10:

- Yeast 2-hybrid assay is not explained well

A10:

We add some sentences to explain yeast two-hybrid assay in the flowing (P12 L258-263):

All the truncated N-terminal TIN1 protein interacted with the TPL/TPR/REL proteins, while all the truncated C-terminal TIN1 protein without the EAR motif did not interact with the TPL/TPR/REL proteins (Fig. 5j, see Materials and Methods). This result suggested that TIN1 directly interacts with these three TPL/TPR/REL proteins through the C-terminal EAR motif during maize tiller development.

Q11:

- Tin1 selection assay – there is no explanation for why the authors conclude that tin1 has been under parallel selection in opposite directions across different cereals. This would be an important finding if well-supported.

A11:
Wild progenitors of the cereals generally have more tiller number than domesticated cereals, the selection of tin1 led to repressing tiller number during rice, foxtail millet and sorghum domestications. While the selection of tin1 resulted in increasing tiller number in sweet corn and popcorn. Thus the tin1 has been under parallel selection in opposite directions across different cereals. To explain this point clearly, we added some explanations in the revised manuscript (P 15 L312-315):

The selection of tin1 resulted in the decreased tiller number during rice, foxtail millet and sorghum domestications, while the selection of tin1 led to the increased tiller number in sweet corn and popcorn during maize diversification. These results supported that tin1 was under parallel selection in opposite directions across different cereals.

Q12:
Discussion
- I agree with the authors that they have evidence for a role for tin1 in repressing bud dormancy and for stimulating growth
A12:
Thank you.

Q13:
- It is really not clear to me that the authors have evidence for a 'de-domestication' of maize with the tin1 mutation, regardless of the percentages of tin1 in the different types of maize. The stacking of mutants could have gone the other way, especially because we know that the popcorns were the earliest domesticated.
A13:
We agree with Reviewer#1 that sweet corn and popcorn are the early domesticated types. The selection of tin1 from teosinte led to accumulating the tin1 allele with more tiller number in sweet corn and popcorn. This process is not a " de-domestication ". Thus we removed all the words related to "de-domestication".

Q14:
- It is surprising that the foxtail millet results and indeed all comparative syntenic analyses that were presented in the Results are absent from the Discussion.
A14:
We added a paragraph to discuss the *tin1* loci across different species based on Reviewer#1's comment.

Parallel phenotypic changes during domestication and diversification might share similar genetic basis
Comparative genomic analysis of tin1 revealed that a syntenic block harboring four chromosomal fragments on maize chromosome 7, rice chromosome 7, foxtail millet chromosome 2 and sorghum chromosome 2. The tin1 locus contained eight, six and six tin1 duplicated copies in rice, foxtail millet and sorghum, respectively. In maize, only one tin1 copy was identified based on the domesticated maize genome. Whether wild progenitor of maize, teosinte, has multiple *tin1* duplicated copies similar to other grass still needs to be explored.

A parallel transition during domestication is from the loose plant architecture with more tiller number in wild progenitor to the compact plant architecture with less tiller

number in domesticated cereal. In this study, we combined QTL mapping, association mapping and comparative mapping to identify that the *tin1* is responsible for this parallel phenotypic change during domestication. Our result provided a good case that parallel phenotypic changes across different cereals during domestication or diversification might share similar genetic basis. A large deletion with one and a half *tin1* copies was present during domestication in foxtail millet. Such large deletion was also present in rice *tin1* locus.

Q15:
- In many ways the Discussion seems very thin, both in terms of discussing their own results and in drawing conclusions in relation to other known genes and gene pathways.
A15:
Based on the suggestions of Reviewer#1, we added some discussions about tb1, gt1, tru1 and sugary 1 in the following (P16-17 L353-370):

The tin1 gene is another key gene for plant architecture in maize

The key gene, tb1, mainly contributed to maize single stalk during domestication. The expression of tb1 was evaluated to repress the outgrowth of tiller bud in the domesticated maize. tb1 positively regulate the transcriptions of gt1 and tru1. In sweet corn, tiller bud can generally grow out under high expression of tb1 (Fig. 5a). Transcript analysis revealed that tin1 works independently of tb1 (Fig. 5a and b). RNA-seq analysis showed that tin1 repressed the expression of gt1 and did not change the expression of tru1. Further gene network revealed that tin1 was involved in multiple pathways including cellulose synthesis, photosynthesis, and hormone responses. Although some pathways including gt1 might be shared between tin1 and tb1, other specific pathways downstream the tin1 gene might overcome the effect derived from tb1 and control tiller buds to grow out despite high expression of tb1. Our QTL analysis showed that a QTL for tiller number was placed in the end of the long arm of chromosome 4. While sugary 1 was located on the short arm on chromosome 4, which is far away from this tiller number QTL (Supplementary Fig. 1e and f). This result suggested that sugary 1 was not involved in the development of tiller. All these results suggested that tin1 is a key gene for plant architecture in maize.

Q16:
- Again, what is the rationale for the choice of genes they discuss (LABA1 and the topless genes) and those that they don’t (sugary1, and any in-depth discussion of tb1/gt1/tru1).
A16:
We selected Laba1 and the topless genes because these genes were related with tiller development in rice. Laba1, also named as An-2, controls tiller number, awn and yield. In An-2 paper, An-2 was responsible for tiller number. And the topless genes have been also showed to be associated with the development of tiller bud in maize (Liu et al, Plant Physiology, 2019). We added an intensive discussion of the three genes (tb1/gt1/tru1) related to tiller and sugary 1 in maize in the revised MS. Please check the discussion in **A15**.

Liu, X., Galli, M., Camehl, I. & Gallavotti, A. RAMOSA1 ENHANCER LOCUS2-Mediated Transcriptional Repression Regulates Vegetative and Reproductive

Architecture. Plant Physiology 179, 348-363 (2019).

Summary

The tin1 gene appears novel, and to act as a enhancer of tiller growth in maize. It is a valuable addition to our knowledge on branching in grasses. However, the authors fail to put their work in context or to adequately discuss tb1/gt1/tru1, or discuss at all relevant literature on other genes isolated from sweet corn such as sugary1. The idea of a de-domestication of maize, while novel, is not convincingly supported, or even well-tested, by their analyses. In its present form, it is unlikely to influence thinking in the field of branching regulation.

Reviewer #2 (Remarks to the Author):

In this manuscript, Zhang et al analyze a tillering trait associated with sweet and popcorn maize. By QTL analysis and use of RILs they identified a single C2H2 zinc finger transcription factor, TIN1, as a likely candidate. By overexpression they showed that they can increase tillering in B73 maize. The molecular cause is narrowed down by association analysis to a single SNP in the 5'UTR that produces a splice variant in intron1. This variant is shown to increase stability of the transcript, so the model goes that increased TIN1 transcript stability promotes tillering in sweet and popcorn maize. The TIN1 TF has a repressive EAR motif at the C-terminus and they show that TIN1 interacts with maize TPL proteins, likely functioning as a transcriptional repressor. By transient luciferase assay and RNA-seq analysis they identified possible direct targets of TIN1 repression, among which is GT1, a domestication tillering gene in maize. Given the importance of tillering in grass domestication, by broadening the analysis of TIN1 to other grass species they found association between TIN1 and tillering in foxtail millet and sorghum and evidence of selection in maize, sorghum and foxtail millet.

Tillering is an important agronomic trait in several crop species and understanding its regulation is crucial for the modification of plant architecture. This manuscript presents a large amount of data and work, and it presents very interesting results. However, there are several concerns that need to be addressed.

Main concerns:

Q17:

1) The logic for the overexpression experiment is missing. It makes more sense if presented after presenting the splice variant analysis, not before.

A17:

OK. We combined fine mapping of tin1 with the splice variant analysis into a section, and then put overexpression experiment after this section in the revised manuscript. Please see P5-7 L107.

Q18:

2) The authors present transformation lines in the maize inbred background B73 but do not provide any details of how this transformation was obtained. B73 is notoriously recalcitrant to transformation and this is by no mean a simple task. Details on the transformation protocol must be provided.

A18:
We performed transformation in maize inbred line B73 using a normal protocol, which was generally conducted in Hi II. It is really difficult to perform transformation in B73, we spent one year and only obtained four events. We then added a reference for transformation:

Vega, J.M., Yu, W.C., Kennon, A.R., Chen, X.L. & Zhang, Z.Y.J. Improvement of Agrobacterium-mediated
transformation in Hi-II maize (Zea mays) using standard binary vectors. *Plant Cell Reports* **27**, 297-305 (2008).

Q19:
3) The analysis of TIN1 transcripts in B73 should be provided. The expectation is that in B73 the relative contribution of the tin1.1 spliced variant should be lower than in P51.
A19:
We firstly introduced tin1 overexpression vector into maize inbred line B73. The T_0 transgenic plants were then back-crossed with the parental line B37 (NOT B73) for one time, and next the back-crossed plants were self-pollinated for two generations (see Methods).

We performed real-time qPCRs from B73 and P51 in tiller bud. The expression level of tin1 is clearly lower in B73 than that in P51. This result was shown in Supplementary Figure 3.

Q20:
4) The in situ results in Fig4 are not convincing. Arguably, this signal could be simply background noise.
A20:
In situ hybridization, real-time qPCR and RNA-seq revealed that the tin1 gene has a very low expression level in tiller bud. We also conducted in situ hybridization for tin1 again. The signals of tin1 still can not be clearly distinguished from background. Thus we removed in situ hybridization in the revised MS.

Q21:
5) Lines 228-239: The authors re-named incorrectly all TPL/TPR proteins of maize. Use the nomenclature previously published (see Liu et al Plant Phys 2019). Your nomenclature does not match the phylogeny classification (i.e. TPL2/REL2; Arabidopsis does not have a clear REL2 ortholog). Also, there are four TPL-like genes in maize called REL2/RELK1-3, all expressed ubiquitously (not three). It would be very surprising that one of the 4 members is not expressed in buds (line 231).
A21:
Based on this comment, we then checked the paper published on Plant Physiology. Maize contained four TPL-like genes: RELK1, RELK2, RELK3 and REL2. We re-analyzed the expressions of these four genes in maize tiller bud based on RNA-seq. All these four genes can express in maize tiller buds. However, the expression of RELK1 was very low. Based on these descriptions, we then cite this paper published on Plant Physiology, and put the expressions of these four TPL-like genes in supplemental Figure 14, and rephrased several sentences in the following:

There are four TPL/TPR/REL genes expressed in maize tiller bud, namely RELK1 (Zm00001d040279), RELK2 (Zm00001d028481), RELK3 (Zm00001d047897) and REL2 (RAMOSA1 ENHANCER LOCUS223, Zm00001d024523)[23] (Supplementary Fig. 14). RELK1 showed low expression level in maize tiller bud.

Q22:
6) Line 300: "Maize tin1 responded to light stimuli.." No evidence is provided that this is the case.
A22:
We rephrased this sentence as " Maize tin1 might respond to light stimuli…"

Other concerns and suggestions:
Q23:
7) Modify Figure 3C, add entire gene model. As presented it looks like the intron retention is occurring in the coding sequence, not 5'UTR and it is confusing to the reader.
A23:
Thank you for the suggestion. We added a stop codon (*) in the gene model.

Q24:
8) Be consistent with nomenclature between text and figures, tin1.1 or tin1-1, do not switch between the two.
A24:
Thank you. tin1.1 and tin1.2 had been replaced with tin1-1 and tin1-2 in the figures.

Q25:
9) Line 231: change "proteins" in "genes"
A25:
OK. Thank you.

Q26:
10) Move lines 334-339 into Result section
A26:
OK. We moved this part to Result in the revised MS.

Q27:
11) Line 115: correct "self-crossed twice"
A27:
We firstly introduced tin1 overexpression vector into maize inbred line B73. The T_0 transgenic plant was then back-crossed with the parental line B37 (NOT B73) for one time, and next the back-crossed plants were self-pollinated for two generations (see Methods). So we changed these words with "self-pollinated for two generations".

Q28:
12) Provide details on the inbred line used for the protoplast assay.
A28 :
We used B73 and NIL-P51 leaf protoplast. We added this information in Methods.

Q29:
13) Lines 295, 342: delete "Our"

A29:
OK.

Q30:
14) Add labels to Figure 1 plants (i.e. sweet corn etc.)
A30:
OK. Thank you.

Reviewer #3 (Remarks to the Author):

This manuscript describes the positional cloning of a QTL that increases tiller number in sweet corn. QTL and fine mapping identified a single gene, tin1, encoding a C2H2 zinc finger protein that likely acts as a transcriptional repressor. This gene is an ortholog of the previously identified rice QTL prog1 which promotes an upright growth habit. A single nucleotide polymorphism at a 5’ splice site of tin1 was found to inhibit splicing of a 5’ non-coding intron, resulting in a decreased level of RNA decay for the altered transcript. Increased expression of tin1 leads to a decrease in the known tillering regulator gt1, but has no effect on the other major tillering domestication locus in maize tb1. Tin1 interacts physically with maize orthologs of TOPLESS, via the conserved EAR domain. Transcript profiling of tin1 shows that multiple hormone pathways are targeted, as well as photosynthetic machinery. The authors show that tin1 has been selected in parallel in setaria and sorghum as both have QTL for tillering that map to the tin1 ortholog. The setaria QTL has a deletion that includes 2/5 copies of a tandem duplication. Finally, they argue that tin1 was selected for increased tillering in sweet and popcorn maize lines by introgression from standing diversity in teosinte.

The positional cloning and partial characterization of this maize tillering QTL represents an impressive amount of work, and the authors present a convincing case that they have identified the correct gene. The case for the splice site mutation as causative is interesting, but not yet completely convincing (see below). Even without an airtight case that they have identified the causative polymorphism, this work is likely to be of broad interest as it identifies a new gene regulating tillering in maize, and likely in other closely related panicoid cereals. Beyond that, tin1 is a QTL regulating a distinct domestication phenotype in rice and thus provides another example of how variation at a small subset of genes has been selected in parallel, sometimes for similar and sometimes for divergent phenotypes.

While I am generally excited about this work, I have several concerns about the characterization as it currently stands. I detail my major concerns below followed by other minor comments.

Major concerns:
Q31:
1. The splice site mutation is an intriguing possibility for the cause of the phenotype. However, at this point, other polymorphisms in the promoter (in LD with the splice mutation) could also contribute. The primary evidence against the promoter mutations as causative is a luciferase assay of the promoters from the parents tested in maize protoplasts. While this assay shows no significant difference in expression, that does

not mean the promoter polymorphisms are insufficient for differential expression in a different tissue context. Promoters are highly context dependent, and that is likely to be the case for tin1, as shown in S9. Indeed, the expression results presented in S9 provide additional reasons to be skeptical that differential splicing causes overexpression of the P51 allele. It is clear from S9 that overexpression of the P51 allele is not global, and both alleles are equally expressed in leaf and root, while the differential expression is unique to the tiller bud and SAM. Why wouldn't this splice mutation lead to reduced RNA decay in all tested tissues? A simple confirmation that the splice site is indeed altered would be to sequence the alternate transcripts. A follow up would be to test the relative abundance of each transcript in the various tissues.

Sequence analysis of the alternate transcripts would still not confirm the SNP as causative as that would require ruling out the promoter SNPs by recombination or a comparison of transgenic lines that differ only presence of the SNP. I understand that this is a significant amount of additional work, and I am not arguing that it is needed for publication, only needed to make a strong argument that you can rule out the promoter polymorphisms.

A31:

We thank you for this comment. Based on the suggestion, we performed RT-PCRs to amplify the transcripts of tin1 in leaf and root. Only tin1-1 transcript with the intron was present, and tin1-2 transcript without the intron was absent in leaf and root. This result suggested that alternative splicing of tin1 was only present in tiller bud and SAM, that is, alternative splicing of tin1 is tissue dependent. Therefore the splicing site variant from "G/GT" to "C/GT" did not change the expression level in leaf and root between the two NILs. We still can not completely rule out that the variants in the promoter may contribute to expression. We added this in Results:

(P7 L140-144)

Although the luciferase transient expression of promoter did not detect the changed signals in leaf between the two parental lines B37 and P51. The variants in the promoter with high LD with the splice-site variant might also contribute to the expression levels of *tin1* in tiller bud, because the effect of the promoter of *tin1* might be tissue dependent.

(P9 L193-199)

...However, the expression of tin1 was equal between NIL-P51 and NIL-B37 in leaf and root. RT-PCR then revealed that only the tin1-1 transcript with the intron was present and the tin1-2 transcript without the intron was absent in leaf and root (Supplementary Fig. 10). This result suggested that alternative splicing is tissue dependent. Therefore the splicing-site variant from "G/GT" to "C/GT" did not change the expression levels of tin1 between the two NILs in leaf and root.

Q32:

2. The final section of the Discussion introduces novel data not presented in the results arguing that the sweet corn tin1 allele originated from introgression from teosinte. At least I think that is the argument. However, a much stronger population genetic analysis would be needed to argue this. It is clear that the splice site mutation is present in both maize and teosinte, but that is true for most alleles in maize. Furthermore, the splice site allele is not fixed in either sweet corn or popcorn, and is often present in other maize lines (although more common in sweet corn). This does

not seem to me like the kind of pattern you would expect for strong selection of tin1 after introgression from teosinte. Indeed, the reduced polymorphism seen for the splice-site allele (fig 6e) is exactly what you would expect if you select just one allele. I'm not an expert in tests for selection, but this doesn't seem to include the proper comparisons to make the case that selection is involved. It shows only that polymorphisms in a region including the promoter and first intron/exon of tin1 are in LD, that could be due to selection, or to other factors (e.g. reduced recombination).

A32:

Our new phylogenetic tree (Supplemental Figure 18b) based on whole genome sequences (HapMap III) signified that both sweet corn and popcorn were more closer to teosinte than field corn in phylogenetic tree. This fact signified that sweet corn and popcorn are two maize early-domesticated types. Thus the tin1 gene in sweet corn and popcorn should be derived from teosinte, not picked up from maize (field corn). To check whether this tin1 gene is under selection, we then performed selection test for sweet corn and popcorn with the splice-site variant of "C/GT". The sequences of tin1 are completely the same in the promoter and CDS in sweet maize and popcorn (fig. 6e). We performed the HKA test instead of Tajima's test because Tajima's D can not be calculated for the sequences without diversity. Our selection test showed that the tin1 gene was under strong selection in popcorn and sweet corn ($P=8.3\times10^{-27}$). The allele frequency of "C/GT" was 47.1% in sweet maize and popcorn in comparison to teosinte with 18.4%. Selection on tin1 has greatly increased the allele frequency of "C/GT" in sweet corn and popcorn.

Based on these descriptions, we moved the novel data to Results (P15 L319-330). We rephrased Results and Discussion in the following:

(P14 L306-310)

The sequences of sweet maize and popcorn with the "C/GT" allele were completely the same in the promoter, 5' UTR and CDS of the tin1 gene (Fig. 6e). The HKA test significantly ($P=8.3\times10^{-27}$) rejected the neutral null model for the maize *tin1* gene (Fig. 6e, and see Methods), signifying that the *tin1* gene suffered strong selection in sweet maize and popcorn.

(P19 L414-416)

Phylogenetic tree based on genome-wide SNPs revealed that sweet maize and popcorn seemed to be early domesticated types (Supplementary Fig. 18b). This result indicated that the splice-site variant of tin1 might be picked up after tb1 became fixed in maize during diversification. Ancient human may have selected the standing splice-site variant of tin1 from teosinte. Strong selection promptly elevated the allele frequency of tin1 splice-site variant so that sweet corn and popcorn retained tillering.

Q33:

3. The in situs in figure 4I are indistinguishable from background. These should be removed entirely or some convincing localization should be presented.

A33:

Thank you for the suggestion. In situ hybridization, real-time qPCR and RNA-seq revealed that the tin1 gene has a very low expression level in tiller bud. We also conducted in situ hybridization for tin1 again. The signals of tin1 still can not be clearly distinguished from background. Thus we removed in situ hybridization in the revised MS.

Minor comments

Q34:

1. The English is hard to follow in places and the entire manuscript would benefit from careful editing by a native English speaker.

A34:

Thank you for the suggestion. This manuscript has been intensively edited by a senior Editor from Plant Editors (https://planteditors.com/). We then carefully edited the revised manuscript for several times.

Q35:

2. Pg 3. Line 58-64, also pg. 16 line 343-345. Tillering is increased in sweet and popcorn, but this study only looks at one line of sweet corn. It is not clear that tin1 is important for tillering in popcorn, or even for all sweet corn lines as it is not fixed in sweet corn.

A35:

We investigated tillering in maize association panel with 263 maize inbred lines. So we measured tiller number in both sweet corn and popcorn. Sweet corn and popcorn generally contain 2-4 tillers, and about half popcorn has the tin1 allele with "C/GT". This signified tin1 also plays an important role in tillering in popcorn.

Q36:

3. Pg 5 line 96. The transformation presented in this section is not a “complementation analysis” as no LOF phenotype was complemented by introduction of a WT copy. Rather this tests if overexpression of tin1 is sufficient to cause a tillering phenotype, which it is.

A36:

Thank you. We replaced "complementation analysis" with " Overexpression of tin1 through transformation increased tiller number in maize "

Q37:

4. Pg 8 line 166-9. TIN1-YFP shows localization to both nucleus and cytoplasm, I was not convinced that it was “mainly” in the nucleus. Expression in the nucleus is not sufficient reason to conclude it is a TF, but in combination with the other sequence features is consistent with that conclusion.

A37:

We performed TIN1-GFP subcellular localization again, and the signals were present in both nucleus and cytoplasm. We then rephrased the sentence in the following :

TIN1-GFP fusion protein was localized in the nucleus and cytoplasm in both onion epidermal cells and maize protoplasts (Fig. 4k and Supplementary Fig. 8).

Q38:

5. pg. 9 line 183 to 185. I’m not sure that you can make a statement that tin1 acts in parallel to tb1 with the data at hand. A strong case for parallel action would require an analysis of a tin1-lof tb1-lof double mutant. The data right now only indicates that tb1 does not regulate transcription of tin1 and tin1 does not regulate transcription of tb1. There could be genetic or physical interactions between tin1 and tb1 beyond a direct transcriptional interaction, but these were not tested.

A38:

We changed " in parallel to tb1 "with " independently of tb1" in the revised MS.

Q39:
6. Pg 12 line 261. There are only 6 copies of tin1 in setaria, and there is not SiTin1-6 in the phylogeny.
A39:
Thank you. We corrected this phylogenetic tree in the revised MS.

Q40:
7. Pg 13 line 289. Cannot say that tin1 corresponds to the tillering QTL in sorghum, only that it is linked.
A40:
We changed "corresponding to" with "linked with".

Q41:
8. pg 14 line 298-301. While gt1 has been shown to respond to R:FR ratio during tillering, there is no evidence that tin1 is in the SAR pathway. Rather tin1 regulates gt1, i.e. it regulates a downstream effector of the SAR. There is no data presented that tin1 responds to light stimuli.
A41:
Thank you. We changed this with" Maize tin1 might respond to light stimuli ".

Q42:
9. pg 15 line 315-326. The argument that tin1 was selected to increase the ears and thus yield of sweet corn and popcorn doesn't make much sense. If tin1 was selected for increased yield in these lines, why was it not selected for a similar yield increase in non-sweet or non-popcorn lines? Why would it only increase yield in these specialized lines?
A42:

In some previous studies, the removal of tiller decreased the production of sweet maize. Sweet corn hybrid generally has several tillers. The carbohydrate might move from tiller without ear to the ear on maize main stalk. Or the removal of tiller with ear directly decreases ear number. Thus the yield will be decreased in sweet corn if the tiller is removed. In this study, we overexpressed *tin1* in B73, both tiller number and ear number were significantly increased in the transgenic plants compared with nontransgenic plants. The tillers on some field corn do not generally produce ear, whether tillering can improve yield in field corn remains unknown. And it is just an argument, still need to be explored in the future. One study can not resolve all the questions. We added the references and a sentence in the revised MS:

In some previous studies, the removal of tiller decreased the production of sweet maize[35, 36].

35. Hanna HaS, R. Yield of supper sweet corn as affected by N application timing, plant density, tiller removal, and insecticides. Proceedings of the Florida State Horticultural Society 105, 343-344 (1992).

36. Akman Z. Effect of tiller removing and plant density on ear yield of sweet

corn (Zea mays saccharata Sturt). Pakistan Journal of Biological Science 5(9), 906-909 (2002).

Q43:

10. Is there only one tin1 copy in maize, while other cereals all have multiple tandem duplicates? If so that seems strange, and worthy of some discussion.

A43:

We added a discussion (P17-18 L375-381):

Comparative genomic analysis of tin1 revealed that a syntenic block harboring four chromosomal fragments on maize chromosome 7, rice chromosome 7, foxtail millet chromosome 2 and sorghum chromosome 2. The tin1 locus contained eight, six and six tin1 duplicated copies in rice, foxtail millet and sorghum, respectively. In maize, only one tin1 copy was identified based on domesticated maize genome. Whether wild progenitor of maize, teosinte, has multiple *tin1* duplicated copies similar to other grass still needs to be explored.

Reviewers' comments:

Reviewer #1 (Remarks to the Author):

I will assess the extent that the authors answered my previous comments and questions and then make some further remarks.
Q1. The authors suggestion to change the wording of the title to "The tin1 gene retains tillering in maize" is OK, although changing retains to promotes might be better.
Q2. I appreciate that the authors have now provided a more accurate view of the place of sweet corn and popcorn in the phylogeny of maize.
Q3. Shortness of introduction. I appreciate the extra paragraph on other tillering genes, although I do not know why the authors remain dubious about sugary 1, saying "sugary 1 was supposed to change the carbohydrate balance". I am not aware that the data on that gene is less surely based than those of other genes.
Q4. Results. These have been improved although there are still some unanswered questions and anomalies, as follows:
• Line 99, Supp Fig. 1f is cited as evidence for homozygosity of the genome, but actually shows the genome position of the su1 locus.
• Lines 140-141. The sentence is incomplete.
• Lines 294-299. This paragraph and Supp Fig 17 describes results from foxtail millet and sorghum but presents no data or references. For example, the statement is made that deletion of one and a half tin1 copies in foxtail millet leads to a reduction of 4.75 tillers, yet no data is shown to back up this statement. The comparative genetic argument is an important one, but the data needs to be shown.
• Line 284. The 18 kB fragment is only absent in some of the domesticated foxtail millet lines, yet the phenotype is for all the lines. Do the lines differ in branching in ways not described in the text that would account for this anomaly?
• Line 309. Is there selection for tin1 in common maize, was this checked?

Reviewer #2 (Remarks to the Author):

I have reviewed a previous version of this manuscript.

In general the manuscript should be heavily edited for English i.e. line 65 "…encoding a TCP domain"; line 67 "…with a homeodomain protein response to shade…"; line 70 "…encoding an ankyrin-repeat domain …"; line 72: …"sugary1 was supposed to change the…"??

Specific comments:

1. The splicing analysis (the entire paragraph where the enhanced expression and stability of tin1-1 is presented) should be moved before the overexpression section, especially given that the promoter:luciferase experiment did not show any expression difference…why create overexpression lines?? The logic of this experiment is missing. However, the overexpression lines show that increasing levels of TIN1 promote tillering and this is independent of the coding sequence, hence presumably is due to the stability of the transcripts.

2. In Supplementary Fig 1a, are there actually 2 plants in P51 with 1 tiller each? Tillers normally have feminized tassels.

3. Line 94: Figure 3 is mentioned in the text before Figure 2.

4. Where is Supplementary Fig3 in the text?

5. Line 128: what is a "natural maize inbred population from around the world"?

6. Lines 140-144: this paragraph does not make sense.

7. line 285: " ….Supplementary file and Supplementary Fig. 16..”?

8. lines 337-338: " gt1 was directly targeted by tin1..” add "in transient assays, suggesting that…”

9. line 349-351: this sentence does not make sense…if it interacts with TPL related proteins why would TIN1 promote expression of downstream targets

10. Fig 5h. if the control effector construct is empty as specified in Methods, the diagram should be shortened otherwise it looks like a random sequence was inserted

11. The in situ hybridization section should be removed from the Methods section, not only as a Figure.

12. No information is given regarding how they genotyped their transformant apart from "…the BAR gene was amplified …” provide sequences of primers used; actually, provide all primer sequences used for qRT-PCR.

Reviewer #3 (Remarks to the Author):

This revised manuscript addresses my main concerns with the inclusion of some new data, including RT-PCR analysis of alternate transcripts from multiple tissues, and a new phylogenetic tree. The other main concern about in situ quality was addressed by removing that data. At this point, I don't think that any more data is needed. However, I still don't feel that the text makes clear where the data is ambiguous. In addition, I remain concerned about the data used to conclude that this gene was under selection in sweet and/or pop-corn.

1. I appreciate the additional data, especially the expression analysis which shows that indeed overexpression of the transcript with intron retention is tissue specific. This new data (in addition to the other data cited in my original review) significantly complicates the simple story presented about a splice site mutation leading to intron retention. While the polymorphism at this splice site could be important, it has not been demonstrated to be causative, and there is plenty of reason to think that something more complex is going on which may or may not include this candidate polymorphism. While a sentence has been added to this effect, the rest of the paper still reads as if the splice site mutation is indeed causative. It would be good to revise this section (and other parts of the paper where the splice site variant enhanced intron retention is assumed to be causative) fully to make it clear that the proposed mechanism does not match all the data. I don't think it is necessary to work out the mechanistic details at this point, only to be upfront where the data does and does not fit the simple alternative splicing mechanism.

2. I am still not convinced that tin1 was under selection in sweet and popcorn. The details of the HKA test for selection are very scant, making it hard to follow what exactly was done here. Again,

population genetics and tests for selection are not my primary expertise, but I have enough questions, that I think this needs to be looked at more carefully and or explained to address the following conerns: This allele is present in teosinte (18.4%), non sweet/pop corn (16.3%) and sweet/popcorn (47.1%). While there is an increase in frequency in sweet/popcorn, it is a long way from fixation in these lines and my intuition says that this increased frequency could be consistent with neutral evolution. In order to do a standard HKA test, you would usually include multiple loci, including neutral loci. What other loci were tested? Also, did you select only those sweet/popcorn lines with this polymorphism to test for selection, or were all alleles present in sweet corn included? If the former, then this bias would of course indicate selection, but it is not meaningful. Since sweet corn/popcorn are not a monophyletic clade, all sweet corn should be compared to an outgroup, and independently all pop-corn should be compared to an outgroup.

Related to this concern, the paper is written as if tin1 is the major tillering gene in sweet/pop corn. However, this cannot be the case since more than half of the sweet and popcorn lines do not have this variant. I assume that these are also tillered, and thus other tillering alleles are more important in these lines. Rather than being the critical allele selected in sweet corn to generate tillers and increase yield (which I am not convinced of by the current data), this could simply be a variant that increases tillering in some sweet and popcorn lines, including the line which was used to create the mapping population. If a QTL was done with a different tillering sweet corn line that doesn't have this variant, an entirely different locus may explain the majority of the variance. If true, this does not diminish the importance of this study to me. The authors have found an important new locus of tillering in maize. I just would like to see more circumspection when talking about the importance of this allele in sweet corn evolution.

Other comments:

1. Paragraph beginning line 140 does not reference any data or figure.

2. line 164-165. The use of tin1-1 and tin1-2 to refer to alternate transcripts is confusing. This nomenclature would usually be used for alleles, not transcripts of the same allele.

3. line 204 the change from "parallel" to "works independently" doesn't really add a new distinction. My original point was that tb1 and tin1 may or may not be functionally independent and the appropriate genetic tests have not been done. All that you know now is that they do not regulate the expression of each other, but that does not rule out other functional interactions that could have real phenotypic consequences.

4. line 328 "ascended to one teosinte line" is not a terminology I am familiar with. You could say that TEO86 is sister to a monophyletic clade that includes maize and teosinte alleles of this gene. However, there are no nodal support values indicated on this tree, and I would be hesitant to make too much out of this. The fact that the allele is present in teosinte suggests that it was standing variation in both maize and teosinte, and does not at all suggest introgression from teosinte to sweet corn.

Reviewers' comments:

Reviewer #1 (Remarks to the Author):

I will assess the extent that the authors answered my previous comments and questions and then make some further remarks.

Question1 (Q1):
Q1. The authors suggestion to change the wording of the title to "The tin1 gene retains tillering in maize" is OK, although changing retains to promotes might be better.
Answer1 (A1):
OK. We changed the title with "The tin1 gene retains the promotion of tillering in maize".

Q2:
Q2. I appreciate that the authors have now provided a more accurate view of the place of sweet corn and popcorn in the phylogeny of maize.
A2:
Thank you.

Q3:
Q3. Shortness of introduction. I appreciate the extra paragraph on other tillering genes, although I do not know why the authors remain dubious about sugary 1, saying "sugary 1 was supposed to change the carbohydrate balance". I am not aware that the data on that gene is less surely based than those of other genes.
A3:
OK. We rephrased the sentence as the following:
In sweet maize, *sugary 1* changes the carbohydrate metabolism balance and may promote tiller bud outgrowth.

Q4. Results. These have been improved although there are still some unanswered questions and anomalies, as follows:
Q4:
• Line 99, Supp Fig. 1f is cited as evidence for homozygosity of the genome, but actually shows the genome position of the su1 locus.
A4:
Supplementary Figure 1e supported homozygosity of the genome. So we removed Supplementary Figure 1f.

Q5:
• Lines 140-141. The sentence is incomplete.
A5:
We rephrased this sentence in the following:
Although the luciferase transient expression of promoter did not detect the changed signals

in leaf between the two parental lines B37 and P51, the variants in the promoter with high LD with the splice-site variant might also contribute to the expression levels of tin1 in tiller bud, because the effect of the promoter of tin1 might be tissue dependent.

Q6:

• Lines 294-299. This paragraph and Supp Fig 17 describes results from foxtail millet and sorghum but presents no data or references. For example, the statement is made that deletion of one and a half tin1 copies in foxtail millet leads to a reduction of 4.75 tillers, yet no data is shown to back up this statement. The comparative genetic argument is an important one, but the data needs to be shown.

A6:

OK. We added phenotype data in supplementary table S7 and a bar chart in Supplementary Figure 17 to support this.

Q7:

• Line 284. The 18 kB fragment is only absent in some of the domesticated foxtail millet lines, yet the phenotype is for all the lines. Do the lines differ in branching in ways not described in the text that would account for this anomaly?

A7:

QTL mapping between wild and domesticated foxtail millet revealed many QTLs for tillering (Doust et al, Genetic control of branching in foxtail millet, PNAS, 2004). And these domesticated lines differ in tiller number (Supplementary table S7), domesticated foxtail millet lines generally bear some small tillers. Some domesticated lines might have more QTLs of tiller number, some lines might have less QTLs. So it is normal that this 18-kb fragment was not present in all domesticated lines.

Q8:

• Line 309. Is there selection for tin1 in common maize, was this checked?

A8:

Tajima's D test did not detect significant selection signal for *tin1* in common maize.

Reviewer #2 (Remarks to the Author):

I have reviewed a previous version of this manuscript.

Q9:

In general the manuscript should be heavily edited for English i.e. line 65 "…encoding a TCP domain"; line 67 "…with a homeodomain protein response to shade…"; line 70 "…encoding an ankyrin-repeat domain …"; line 72: …"sugary1 was supposed to change the…"??

A9:

We rephrased these sentences as the followings:

"…encoding a TCP protein", "…encoding a homeodomain protein response to shade…", "…encoding a protein with an ankyrin-repeat domain …", "sugary1 changes the…"

Specific comments:

Q10:

1. The splicing analysis (the entire paragraph where the enhanced expression and stability of tin1-1 is presented) should be moved before the overexpression section, especially given that the promoter:luciferase experiment did not show any expression difference…why create overexpression lines?? The logic of this experiment is missing. However, the overexpression lines show that increasing levels of TIN1 promote tillering and this is independent of the coding sequence, hence presumably is due to the stability of the transcripts.

A10:

Thank you. We moved this section before overexpression section.

Q11:

2. In Supplementary Fig 1a, are there actually 2 plants in P51 with 1 tiller each? Tillers normally have feminized tassels.

A11:

No, just one P51 plant. P51 generally has several tillers with ear. We do not observe feminized tassels from tillers of P51 in both Beijing (high latitude) and Hainan (low latitude).

Q12:

3. Line 94: Figure 3 is mentioned in the text before Figure 2.

A12:

We changed it with Supplementary Fig. 1d.

Q13:

4. Where is Supplementary Fig3 in the text?

A13:

Supplementary Fig.3 was in main text (P8 L172) in the overexpression section.

Q14:

5. Line 128: what is a “natural maize inbred population from around the world”?

A14:

We rephrased this sentence as “diverse maize inbred lines from around the world”.

Q15:

6. Lines 140-144: this paragraph does not make sense.

A15:

Based on Reviewer#3’s comment, we added this paragraph.

Q16:

7. line 285: “ ....Supplementary file and Supplementary Fig. 16..”?

A16:

We changed as “Supplementary Data and Supplementary Fig. 16”. Supplementary Data showed the sequences of tin1 from foxtail millet.

Q17:
8. lines 337-338: " gt1 was directly targeted by tin1.." add "in transient assays, suggesting that…"
A17:
OK. Thank you.

Q18:
9. line 349-351: this sentence does not make sense…if it interacts with TPL related proteins why would TIN1 promote expression of downstream targets
A18:
We rephrased this sentence as" and then controlled the downstream targets "

Q19:
10. Fig 5h. if the control effector construct is empty as specified in Methods, the diagram should be shortened otherwise it looks like a random sequence was inserted
A19:
OK. We changed the bar length in Figure 5h.

Q20:
11. The in situ hybridization section should be removed from the Methods section, not only as a Figure.
A20:
We removed this section from Method.

Q21:
12. No information is given regarding how they genotyped their transformant apart from "…the BAR gene was amplified …" provide sequences of primers used; actually, provide all primer sequences used for qRT-PCR.
A21:
We added all the primers in Supplementary Table S8.

Reviewer #3 (Remarks to the Author):

This revised manuscript addresses my main concerns with the inclusion of some new data, including RT-PCR analysis of alternate transcripts from multiple tissues, and a new phylogenetic tree. The other main concern about in situ quality was addressed by removing that data. At this point, I don't think that any more data is needed. However, I still don't feel that the text makes clear where the data is ambiguous. In addition, I remain concerned about the data used to conclude that this gene was under selection in sweet and/or pop-corn.
Q22:
1. I appreciate the additional data, especially the expression analysis which shows that indeed overexpression of the transcript with intron retention is tissue specific. This new data (in addition to the other data cited in my original review) significantly complicates the

simple story presented about a splice site mutation leading to intron retention. While the polymorphism at this splice site could be important, it has not been demonstrated to be causative, and there is plenty of reason to think that something more complex is going on which may or may not include this candidate polymorphism. While a sentence has been added to this effect, the rest of the paper still reads as if the splice site mutation is indeed causative. It would be good to revise this section (and other parts of the paper where the splice site variant enhanced intron retention is assumed to be causative) fully to make it clear that the proposed mechanism does not match all the data. I don't think it is necessary to work out the mechanistic details at this point, only to be upfront where the data does and does not fit the simple alternative splicing mechanism.

A22:

The splice site variant was identified as causative variant based on three facts: 1) The strongest association signal was present at this site. 2) The transcription level of tin1-T2 with an intron was stronger than that of tin1-T1 without this intron. 3) RNA of tin1-T2 was proved to be more stable than tin1-T1 RNA in tiller bud. Although we do not know why the alternative splicing of tin1 was only present in tiller bud, the splice site variant could be the causative to maize tin1. WE THEN LOWERED THE TONE AND MADE SOME CHANGES IN THE ABSTRACT AND MAIN TEXT. We added the word "might" in several places.

Q23:

2. I am still not convinced that tin1 was under selection in sweet and popcorn. The details of the HKA test for selection are very scant, making it hard to follow what exactly was done here. Again, population genetics and tests for selection are not my primary expertise, but I have enough questions, that I think this needs to be looked at more carefully and or explained to address the following conerns: This allele is present in teosinte (18.4%), non sweet/pop corn (16.3%) and sweet/popcorn (47.1%). While there is an increase in frequency in sweet/popcorn, it is a long way from fixation in these lines and my intuition says that this increased frequency could be consistent with neutral evolution. In order to do a standard HKA test, you would usually include multiple loci, including neutral loci. What other loci were tested? Also, did you select only those sweet/popcorn lines with this polymorphism to test for selection, or were all alleles present in sweet corn included? If the former, then this bias would of course indicate selection, but it is not meaningful. Since sweet corn/popcorn are not a monophyletic clade, all sweet corn should be compared to an outgroup, and independently all pop-corn should be compared to an outgroup.

A23:

Reviewer#3 thought that an allele must get fixed when an allele is under selection. This is not true. Only two maize genes under human selection including tb1 for tiller number and tga1 for naked grain have been proved to be fixed in domesticated maize. Teosinte has long lateral branches that bear multiple small ears at their nodes and tassels at their tips. Maize has much shorter lateral branches that are tipped by a single large ear with no additional ears at the branch nodes. The difference in prolificacy was controlled by several QTLs including a major QTL prol1.1 (Wills et al, From Many, One: Genetic Control of Prolificacy during Maize Domestication, Plos genetics, e1003604, 2013). The allele frequencies of prol1.1 with maize distinct haplotype were 71% in domesticated maize and 13% in teosinte

(Table 2, Wills et al, Plos genetics, e1003604, 2013), respectively. This allele in prol1.1 is far away from fixation in maize, however, this locus has been under selection during domestication. Other QTLs may be selected for the rest 29 % of domesticated maize line. Even for the key trait nonshattering in maize domestication, we did not find that two major QTLs for nonshattering got fixed during domestication (Figure 5, Lin et al, Parallel domestication of the shattering 1 genes in cereals, Nature Genetics, 44:720-724, 2012). However these two nonshattering loci were clearly under strong selection. In our tin1 case, the allele frequency research 47.1% in sweet corn and popcorn, much larger than the frequencies in teosinte (18.4%) or common maize (16.3%). Our HKA test suggested that tin1 was under selection in sweet corn and popcorn with the splicing variant, which was consistent with the completely same sequences of tin1 in sweet corn and popcorn with the splicing variant. Reviewer#3 also argued that the increase allele frequency could be due to neutral evolution, however, tillering is not a neutral trait, and random genetic drift in neutral evolution will quickly increase the allele frequency and get fixed soon. Our phylogenetic tree revealed that sweet corn and popcorn were two old domesticated maize types, which should be domesticated thousands of years ago. The allele frequency of tin1 would be soon fixed in sweet corn and popcorn if random genetic drift works in this long time. Therefore, if tin1 was not under selection, we really do not know which force can keep these sequences to remain the same.

As for HKA test, we applied Zea diploperennis as an outgroup and six neutral loci as control loci from the study of tb1 (Studer et al, Nature Genetics, 2011), which we already cited. We calculated HKA for sweet porn and popcorn with the splicing variant because only these lines have the potential of selection. And the signals of selection will be diluted and may not be detected if all sweet corn and popcorn were chose for HKA test. Reviewer#3 argued that HKA test should be calculated separately for sweet corn and popcorn because sweet corn and popcorn were not a monophyletic clade. However, all maize (Zea mays) including sweet corn and popcorn certainly split into a single clade in comparison to Zea diploperennis. HKA tests were also conducted in the study of tb1 (Studer et al, Nature Genetics, 2011) using Zea diploperennis as an outgroup. Base on the logic of Reviewer#3, maize lines in the study of tb1 should be separated into different subgroups and then perform HKA test. No one will perform HKA test like that.

Based on these descriptions, we added several sentences for HKA test in Materials and Methods in the following (P27-28 L596-599):

HKA tests used Zea diploperennis as an outgroup and six neutral loci as control loci, the overall P-value for the HKA test was calculated based on the overall $\chi 2$ value obtained from the sum of the individual $\chi 2$ values for six individual neutral maize loci.

Q24:

Related to this concern, the paper is written as if tin1 is the major tillering gene in sweet/pop corn. However, this cannot be the case since more than half of the sweet and popcorn lines do not have this variant. I assume that these are also tillered, and thus other tillering alleles are more important in these lines. Rather than being the critical allele selected in sweet corn to generate tillers and increase yield (which I am not convinced of by

the current data), this could simply be a variant that increases tillering in some sweet and popcorn lines, including the line which was used to create the mapping population. If a QTL was done with a different tillering sweet corn line that doesn't have this variant, an entirely different locus may explain the majority of the variance. If true, this does not diminish the importance of this study to me. The authors have found an important new locus of tillering in maize. I just would like to see more circumspection when talking about the importance of this allele in sweet corn evolution.

A24:

It is clear that tillering was controlled by some QTLs based on our QTL mapping of tiller number from a sweet/field corn RIL population. The tin 1 is the major QTL for tillering in this RIL population. The allele frequency of the splicing variant reached 47.1% in sweet corn and popcorn from maize association panel. And tin1 can explained 48.5% of total phenotypic variation of tillering in these sweet corn and popcorn lines. Especially for tillering, environment factors account for a large phenotypic variation, that means other genetic factors except for tin1 can seldom explain a large phenotypic variation. Therefore, we believe that tin1 is a key gene for tillering in sweet corn and popcorn.

Other comments:

Q25:

1. Paragraph beginning line 140 does not reference any data or figure.

A25:

We added Supplementary Fig.9 and 10, Fig. 3e.

Q26:

2. line 164-165. The use of tin1-1 and tin1-2 to refer to alternate transcripts is confusing. This nomenclature would usually be used for alleles, not transcripts of the same allele.

A26:

We replaced tin1-1 and tin1-2 with tin1-T1 and tin1-T2.

Q27:

3. line 204 the change from "parallel" to "works independently" doesn't really add a new distinction. My original point was that tb1 and tin1 may or may not be functionally independent and the appropriate genetic tests have not been done. All that you know now is that they do not regulate the expression of each other, but that does not rule out other functional interactions that could have real phenotypic consequences.

A27:

We changed this with "might work independently".

Q28:

4. line 328 "ascended to one teosinte line" is not a terminology I am familiar with. You could say that TEO86 is sister to a monophyletic clade that includes maize and teosinte alleles of this gene. However, there are no nodal support values indicated on this tree, and I would be hesitant to make too much out of this. The fact that the allele is present in teosinte suggests that it was standing variation in both maize and teosinte, and does not at all suggest

introgression from teosinte to sweet corn.
A28:
OK, we changed the words with "were grouped together with one teosinte line".

Reviewers' comments:

Reviewer #1 (Remarks to the Author):

I feel the authors have done an OK job with responding to my concerns. However, I agree with the third reviewer that that evidence for selection is scant and the methodology vague. I also agree that the tone of the paper is still written as if the tin1 gene is the most important factor in tillering in sweet and poopcorns, where this is clearly not the case (given the allelic variation in tin1 in these groups).

Reviewer #3 (Remarks to the Author):

In this revision, the authors have softened the language about the splice site mutation being causative, as suggested. However, the remaining concerns about evidence for selection, and the importance of the tin1 allele in sweet corn tillering broadly were largely ignored, with a series of justifications. I was not convinced by these justifications, particularly in regards to the evidence for selection in sweet corn.

The authors have provided additional details about the HKA test as I requested. I was concerned that the HKA test was not properly applied. With the additional information it is now clear that they only included sweet corn and popcorn lines that contain the same allele in their analysis. As I stated, with this approach it is a foregone conclusion that the tin1 allele will show evidence of selection. Almost any allele you chose (regardless of whether or not it has a phenotype) will appear as if selected by HKA if you ignore all the other alleles in the group of interest. The authors suggest that including the other sweet corn lines would dilute the evidence of selection. That is exactly my point. They should include all the sweet corn lines in the analysis. You can only claim that an allele is under selection in a group if you include all the members of that group.

I am also confused by the reasoning in several of their responses to my concerns. I point out two below where apparent misconceptions about evolution has led them to dismiss what I still hold are valid concerns and were not addressed in the revision.

I'm not sure why the authors insist on keeping these misleading statements and biased analyses in their manuscript. If it is to increase the potential impact of their findings about tin1, I believe that their very convincing work that shows the identity of tin1, with the additional characterization in sorghum and setaria is already enough. There is no need to oversell the importance of tin1, especially when the evidence does not support the claims.

Problematic author responses:
1. " Our phylogenetic tree revealed that sweet corn and popcorn were two old domesticated maize types, which should be domesticated thousands of years ago. The allele frequency of tin1 would be soon fixed in sweet corn and popcorn if random genetic drift works in this long time."

In the previous section the authors argue that selection does not always lead to fixation (which I agree with, incidentally), while here they argue that random drift would? None of the simulations necessary to make such a claim have been done, and it also doesn't follow from the basic expectation that drift only slowly leads to fixation (usually much slower than selection). If drift led to fixation over a few thousand years since domestication, then there would be no standing allelic variation in maize that originated in teosinte, that is clearly not the case!

2. "Base on the logic of Reviewer#3, maize lines in the study of tb1 should be separated into different subgroups and then perform HKA test. No one will perform HKA test like that."

The cited analysis of tb1 appropriately applied the HKA test as they used all domesticated maize (a

monophyletic group from which they did not remove any tb1 alleles or maize lineages) compared to an outgroup. The HKA test generally assumes that you compare a monophyletic group to an outgroup, which Studer et al. have done. However, in this study, the phylogeny makes it clear that sweet corn and popcorn are woefully paraphyletic. This concern, however, is moot given the fact that only lines with the allele in question were included, which invalidates the entire analysis.

Reviewer #4 (Remarks to the Author):

This is a great paper and overall nicely done. I was asked to comment on the HKA and popgen aspects. I agree with a previous reviewer that these need some extra work. See attached for specifics.

Dear Editor and Reviewers,

Thank you for your considerations and time. We applied a better selection test suggested by Reviewer#4. The selection was really present in tin1 in sweet/popcorn.

Sincerely,

Zhongwei Lin

==

Reviewers' comments:

Reviewer #1 (Remarks to the Author):

Comment 1:

I feel the authors have done an OK job with responding to my concerns. However, I agree with the third reviewer that that evidence for selection is scant and the methodology vague. I also agree that the tone of the paper is still written as if the tin1 gene is the most important factor in tillering in sweet and poopcorns, where this is clearly not the case (given the allelic variation in tin1 in these groups).

Response 1:

Thank you for the comment. We applied a better selection test suggested by Reviewer#4 who was expertized in maize population genetics in all the sweet/popcorn lines. Selection was really present in tin1 from all these sweet/popcorn with all haplotypes. We also lowered the tune of this tin1 in sweet/popcorn. We replaced “The tin1 gene is another key gene for plant architecture in maize” with “The tin1 gene is an important gene for plant architecture in maize” in Discussion.

Comment 2:

Reviewer #3 (Remarks to the Author):

In this revision, the authors have softened the language about the splice site mutation being causative, as suggested. However, the remaining concerns about evidence for selection, and the importance of the tin1 allele in sweet corn tillering broadly were largely ignored, with a series of justifications. I was not convinced by these justifications, particularly in regards to the evidence for selection in sweet corn.

The authors have provided additional details about the HKA test as I requested. I was concerned that the HKA test was not properly applied. With the additional information it is now clear that they only included sweet corn and popcorn lines that contain the same allele in their analysis. As I stated, with this approach it is a foregone conclusion that the tin1 allele will show evidence of selection. Almost any allele you chose (regardless of whether or not it has a phenotype) will appear as if selected by HKA if you ignore all the other alleles in the group of interest. The authors suggest that including the other sweet corn lines would dilute the evidence of selection. That

is exactly my point. They should include all the sweet corn lines in the analysis. You can only claim that an allele is under selection in a group if you include all the members of that group.

I am also confused by the reasoning in several of their responses to my concerns. I point out two below where apparent misconceptions about evolution has led them to dismiss what I still hold are valid concerns and were not addressed in the revision.

I'm not sure why the authors insist on keeping these misleading statements and biased analyses in their manuscript. If it is to increase the potential impact of their findings about tin1, I believe that their very convincing work that shows the identity of tin1, with the additional characterization in sorghum and setaria is already enough. There is no need to oversell the importance of tin1, especially when the evidence does not support the claims.

Problematic author responses:

1. " Our phylogenetic tree revealed that sweet corn and popcorn were two old domesticated maize types, which should be domesticated thousands of years ago. The allele frequency of tin1 would be soon fixed in sweet corn and popcorn if random genetic drift works in this long time."

In the previous section the authors argue that selection does not always lead to fixation (which I agree with, incidentally), while here they argue that random drift would? None of the simulations necessary to make such a claim have been done, and it also doesn't follow from the basic expectation that drift only slowly leads to fixation (usually much slower than selection). If drift led to fixation over a few thousand years since domestication, then there would be no standing allelic variation in maize that originated in teosinte, that is clearly not the case!

2. "Base on the logic of Reviewer#3, maize lines in the study of tb1 should be separated into different subgroups and then perform HKA test. No one will perform HKA test like that."

The cited analysis of tb1 appropriately applied the HKA test as they used all domesticated maize (a monophyletic group from which they did not remove any tb1 alleles or maize lineages) compared to an outgroup. The HKA test generally assumes that you compare a monophyletic group to an outgroup, which Studer et al. have done. However, in this study, the phylogeny makes it clear that sweet corn and popcorn are woefully paraphyletic. This concern, however, is moot given the fact that only lines with the allele in question were included, which invalidates the entire analysis.

Response 2:

Thank you for the comments. We then performed a better test based on Fst, suggested by Reviewer#4 who is expertized in maize population genetics, in all these sweet/popcorn with all haplotypes. The selection was detected in the tin1 in all these sweet/popcorn. We also lowered the tune of this tin1 in sweet/popcorn. We replaced "The tin1 gene is another KEY gene for plant architecture in maize" with "The tin1 gene is AN IMPORTANT gene for plant architecture in maize" in Discussion. We added a sentence in MS (P15 L312-314):

The *tin1* gene suffered significant selection (Fst=0.19 between teosinte and

sweet/popcorn, $P<10^{-5}$) in sweet maize and popcorn.

Reviewer #4 (Remarks to the Author):
This is a great paper and overall nicely done. I was asked to comment on the HKA and popgen aspects. I agree with a previous reviewer that these need some extra work. See attached for specifics.

Comment 3:
HKA
Reviewer 3 is correct. The appropriate way to run HKA is to take all individuals in the population where you think selection occurred and test diversity (across all haplotypes). Ascertaining a single haplotype is not correct (and the authors' point that they see no selection at the GGT haplotype does not fix this). Moreover, using 6 small Sanger sequenced loci in2019 seems a bit underwhelming. It's also not clear to me if the authors sequenced these 6 regions in the same set of samples as they sequenced tin1. This is required for the HKA test to be done properly.

There are better tests to use. For example, look at Fst between pop/sweet and other varieties in the regions around tin1.

Other tests
Tajima's D is not a very good test for selection. Which samples were used for Tajima's D? All, or just the foxtail millet- samples? To be convincing, Tajima's D results should be compared to some background expectation (from a demographic model or from genome-wide data, etc.) While Tajima's D can't be analyzed when diversity is zero, there are a number of other approachces, from simulation approaches in gt1 to genome-wide scans.

Response3:
Thank you for the comment. Following your suggestion, we performed selection test based on Fst to detect signals for teosinte and sweet/popcorn, and wild and domestication foxtail millet. Fsts between teosinte and sweet/popcorn with all haplotypes, and wild and domestication foxtail millet with all haplotypes, were 0.19 ($P<10^{-5}$) and 0.34 ($P<10^{-5}$). The results suggested that tin1 was under selection in sweet/popcorn and foxtail millet. Fst was calculated through Arlequin using 10,000 permutations. So we rephrased the sentences in the following (P14L308-310 and P15L312-314):

Significant selection was present (Fst=0.34 between wild and domesticated foxtail millet, $P<10^{-5}$) in the 1.2-kb region flanking the large deletion of 18 kb in foxtail millet (Fig. 6d).

The *tin1* gene suffered significant selection (Fst=0.19 between teosinte and sweet/popcorn, $P<10^{-5}$) in sweet maize and popcorn.

Reviewers' comments:

Reviewer #3 (Remarks to the Author):

I appreciate the willingness of the authors to investigate alternative evidence for selection as suggested by Reviewer #4. While I'm not expert in these analyses, I am still worried that by choosing a teosinte vs. sweet/pop-corn comparison the authors have chosen the wrong comparison group. Tillered sweet and popcorn would have been selected from primarily non-tillered domesticated maize, and thus the comparison should be sweet/popcorn vs all other domesticated maize.

However, since this is mostly outside my research domain, I will defer to the expertise of reviewer #4 if the current analysis is justified.

Reviewer #4 (Remarks to the Author):

While I'm glad to see the authors are trying Fst, it is not implemented correctly. The significant Fst just tells you Fst is not 0. That doesn't say anything about selection. You need a genome-wide comparison of Fst to show that this region shows higher Fst than one would expect. Data to do this are already publicly available. It's also not clear what samples were used for the Fst analysis and why one would compare teosinte to sweet/pop. Why not compare sweet/pop to other maize? The authors need to clearly state what the hypothesis is (selection in what population? compared to what? what's the null they are comparing with -- presumably genome-wide Fst?)

I should also add that I concur (again) with reviewer #3 in all of their statements about the authors' analyses and reasoning.

Comment 1:
As I mentioned in our earlier communications, I would suggest seeking help from a population geneticist who understands how to test for selection. We hope the remaining problems could be solved in this round of revision.
Response1:
Thank you for the comment. We consulted with Dr. Hua Chen in Beijing Institute of Genomics(http://sourcedb.big.cas.cn/yw/pl/fs/BIG_GPM/201610/t20161014_4676049.html), who developed genome-wide XP-CLR test for selection. We then performed XP-CLR selection test for maize tin1. No selection signals were detected in maize tin1 based on maize HapMap 3 SNP data (Supplemental Fig. 19).

Reviewers' comments:

Reviewer #3 (Remarks to the Author):
Comment 2:
I appreciate the willingness of the authors to investigate alternative evidence for selection as suggested by Reviewer #4. While I'm not expert in these analyses, I am still worried that by choosing a teosinte vs. sweet/pop-corn comparison the authors have chosen the wrong comparison group. Tillered sweet and popcorn would have been selected from primarily non-tillered domesticated maize, and thus the comparison should be sweet/popcorn vs all other domesticated maize.

However, since this is mostly outside my research domain, I will defer to the expertise of reviewer #4 if the current analysis is justified.
Response 2:
Thank you for the comment. We applied genome-wide XP-CLR analysis for selection tests to compare sweet/popcorn with teosinte or common maize excluding sweet/popcorn. No selection signals were present between sweet/popcorn and teosinte or common maize. So we rephrased some sentences about the selection of maize tin1. Please check these changes in **Response 3**.

Reviewer #4 (Remarks to the Author):

Comment 3:
While I'm glad to see the authors are trying Fst, it is not implemented correctly. The significant Fst just tells you Fst is not 0. That doesn't say anything about selection. You need a genome-wide comparison of Fst to show that this region shows higher Fst than one would expect. Data to do this are already publicly available. It's also not clear what samples were used for the Fst analysis and why one would compare teosinte to sweet/pop. Why not compare sweet/pop to other maize? The authors need to clearly state what the hypothesis is (selection in what population? compared to what? what's the null they are comparing with -- presumably genome-wide Fst?)

I should also add that I concur (again) with reviewer #3 in all of their statements about the authors' analyses and reasoning.
Response 3:
Thank you for the comment. Following your suggestions, we performed genome-wide composite likelihood ratio (XP-CLR) analysis to detect the selection signals between sweet/popcorn and teosite or common maize excluding sweet/popcorn. We used SNP

data from HapMap 3. The maize contained a subset of maize accessions for HapMap 2, which included 60 common maize, 15 sweet/popcorn and 19 teosinte accessions. Fst and the ratio of diversity (π teosinte / π sweet/popcorn or π maize / π sweet/popcorn) were firstly calculated in the whole maize genome. Then genome-wide XP-CLR analyses for selective tests were performed between sweet/popcorn and teosinte or common maize. No the π ratio, Fst and XP-CLR values in maize tin1 were in the top 5% genome-wide distribution (Supplementary Fig. 19). This result suggested that no selection was present in sweet/popcorn in comparison to teosinte or common maize. Based on these descriptions, we rephrased several sentences about the selection of maize tin1 and added the details of genome-wide selection test in Methods:

P15 L315-318:

Genome-wide composite likelihood ratio (XP-CLR) analysis did not detect any selection signals in maize tin1 (see Methods and Supplementary Fig. 19) between sweet/popcorn and teosinte or common maize.

P19L319-321:

The high allele frequency of this causal variant in sweet/popcorn might be due to random genetic drift because no human selection was present in maize tin1.

P28L601-615:

For genome-wide selection tests for maize tin1, SNP data from maize haplotype version 3 (HapMap 3)[42] were used. The SNPs with a missing data ratio of >10% were filtered. The maize contained a subset of maize accessions for HapMap 2[48], which included 60 common maize, 15 sweet/popcorn and 19 teosinte accessions. Genetic diversity was firstly compared between teosinte and sweet/popcorn, and common maize and sweet/popcorn, using the previous described methods with minor modification[49, 50]. Briefly, Fst and π values were calculated with a 10-kb adjacent non-overlapping window through a 500-bp step using VCFtools[51]. XP-CLR[30] analyses for selective tests between teosinte and sweet/popcorn, and common maize and sweet/popcorn, were then calculated using a non-overlapping window size of 10 kb with a 500-bp step. The average genetic distances for Chromosomes 1 to 10 were estimated to be 0.69, 0.68, 0.70, 0.63, 0.72, 0.66, 0.78, 0.78, 0.84, and 0.75cM/Mb, respectively, using the information from previous studies[52]. For each analysis, the regions with π ratio, Fst and XP-CLR values in the top 5% genome-wide distribution were determined as selective sweeps.

REVIEWERS' COMMENTS:

Reviewer #4 (Remarks to the Author):

I suspect the XP-CLR test as performed is a bit underpowered to pick up soft sweeps etc, but I'm OK with the analyses as run. This a at least a formal test for selection and a more appropriate analysis.

-Jeffrey Ross-Ibarra

REVIEWERS' COMMENTS:

Reviewer #4 (Remarks to the Author):

I suspect the XP-CLR test as performed is a bit underpowered to pick up soft sweeps etc, but I'm OK with the analyses as run. This a at least a formal test for selection and a more appropriate analysis.

-Jeffrey Ross-Ibarra

Response:
Thank you for the comment.
